# Phenazinoates A‒E, five pairs of phenazine conjugates from a mangrove soil-derived *Streptomyces* strain OUCMDZ-4923

**DOI:** 10.1007/s13659-026-00597-0

**Published:** 2026-03-04

**Authors:** Dongyang Wang, Peipei Liu, Yukang Gao, Linmeng Chen, Liping Wang, Ning Li, Weiming Zhu

**Affiliations:** 1 Key Laboratory of Marine Drugs, School of Medicine and Pharmacy, Ministry of Education of China, Ocean University of China, Qingdao, 266003 China; 2 Laboratory for Marine Drugs and Bioproducts, Qingdao Marine Science and Technology Center, Qingdao, 266237 China; 3 Natural Product Research Center of Guizhou Province, Guiyang, 550014 China; 4 State Key Laboratory of Discovery and Utilization of Functional Components in Traditional Chinese Medicine, Guizhou Medical University, Guiyang, 550014 China; 5 Biology Institute, Engineering Research Center of Zebrafish Models for Human Diseases and Drug Screening of Shandong Province, Qilu University of Technology (Shandong Academy of Sciences), Jinan, 250103 China

**Keywords:** Mangrove actinobacteria, *Streptomyces* sp., Phenazine conjugates, Structure elucidation, Antibacterial activity

## Abstract

Phenazinoates A–E (**1**–**5**), comprising five pairs of methyl saphenate conjugates with genistein, *o*-aminophenol, *p*-acetaminophenol and glycerol, were isolated from the fermentation broth of mangrove soil-derived *Streptomyces* sp. OUCMDZ-4923. Their structures were determined through comprehensive one-dimensional and two-dimensional nuclear magnetic resonance spectroscopy, coupled with high-resolution electrospray ionization mass spectrometry. The absolute configurations of each isomer were established by comparing experimental electronic circular dichroism spectra with calculated counterparts. Based on the biosynthetic pathway analysis, compounds **1**‒**3** were semi-synthesized from the reactions of methyl (*R*)-saphenate with genistein, *o*-aminophenol, and *o*-formamidophenol, utilizing microwave-assisted solid acid catalysis. The compounds were resolved as enantiomerically pure forms and subsequently tested for antibacterial efficacy against six pathogenic bacteria. Phenazinoates A (**1**) and B (**2**) demonstrated bioactivity against four Gram-positive bacterial strains, with minimum inhibitory concentration values ranging from 0.78 to 3.13 μg/mL.

**Graphical Abstract:**

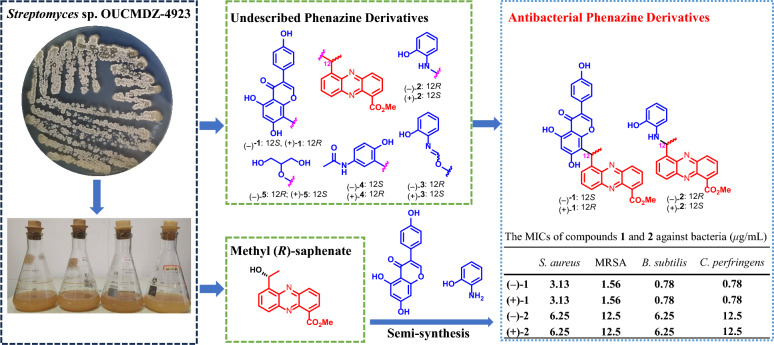

**Supplementary Information:**

The online version contains supplementary material available at 10.1007/s13659-026-00597-0.

## Introduction

**Fig. 1:**
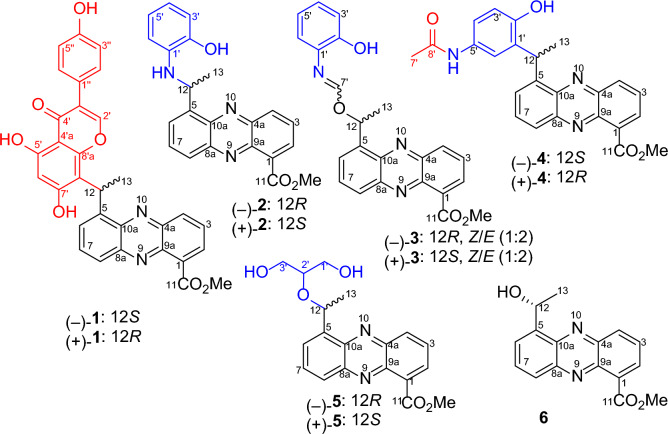
The chemical structures of compounds **1**‒**6**

Natural phenazine derivatives, primarily synthesized by species of *Pseudomonas* and *Streptomyces* [1–3], have been shown to exhibit a broad spectrum of biological activities. These include antibacterial [4, 5], antitumor [6, 7], anti-inflammatory [8, 9], and neuroprotective effects [10, 11]. Our previous work also discovered six phenazine derivatives that demonstrated notable cytotoxicity against tumor cells, potent antifungal activity against *Aspergillus fumigatus*, and activity against the H1N1 virus. These compounds were derived from the marine sponge-associated strain *Nocardiopsis dassonvillei* OUCMDZ-4534 [12]. In our ongoing exploration of bioactive phenazine derivatives of microbial origin, we have been particularly drawn to the untapped metabolic potential of soil-dwelling microorganisms, especially endophytes and their rhizosphere counterparts [13]. Within this context, we focused on a mangrove soil-derived actinobacterial strain, *Streptomyces* sp. OUCMDZ-4923, isolated from mangrove sediment closely associated with the roots of *Kandelia candel*. Bioinformatics analysis confirmed the presence of a phenazine biosynthetic gene cluster (*pnz*BGC) in *Streptomyces* sp. OUCMDZ-4923 (GenBank accession No. CP170384), underscoring its potential to produce phenazine and its derivatives [14–16]. To pursue these bioactive compounds, a large-scale 60-L fermentation of *Streptomyces* sp. OUCMDZ-4923 was carried out. Chemical isolation of the ethyl acetate (EtOAc) extract resulted in the identification of sixteen enantiomerically pure phenazine dimers [16]. Further isolation efforts led to the discovery of five pairs of novel phenazine conjugates consisting of methyl saphenate linked with genistein, *o*-aminophenol, *p*-acetaminophenol and glycerol. These compounds were designated as phenazinoates A–E (**1**–**5**), along with the previously reported methyl (*R*)-saphenate (**6**) (Fig. 1) [16, 17]. Among the newly discovered compounds, phenazinoates A (**1**) and B (**2**), which are conjugates of genistein and *o*-aminophenol with methyl saphenate, demonstrated notable antibacterial activity against four strains of Gram-positive pathogenic bacteria, exhibiting minimum inhibitory concentration (MIC) values ranging from 0.78 to 3.13 μg/mL.

## Results and discussion

### Structural elucidation of phenazinoates A–E (1–5)

**Fig. 2:**
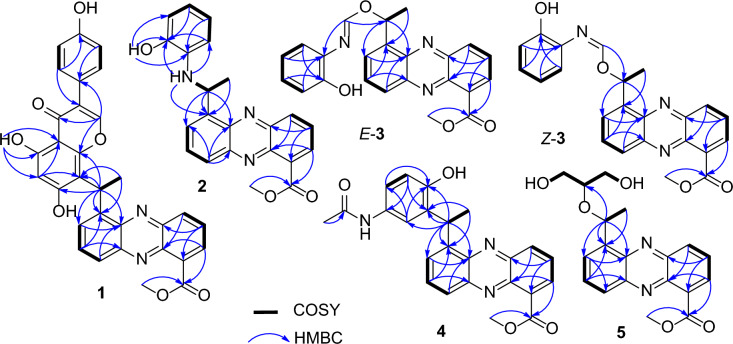
Key COSY and HMBC correlations for the structural assignment of compounds **1**‒**5**

Phenazinoate A (**1**) was isolated as a racemic mixture in the form of a yellow powder. The molecular formula was established as C_31_H_22_O_7_N_2_ based on the high-resolution electrospray ionization mass spectrometry (HRESIMS) at *m*/*z* 535.1502 for the [M + H]^+^ ion (calcd for C_31_H_23_O_7_N_2_^+^, 535.1500). The UV–Visible absorption spectrum of the compound closely resembled those of known phenazine analogs, suggesting that the compound is indeed derivative of the phenazine family. The elucidation of the structure of compound **1** was further supported by the correlation spectroscopy (COSY) and heteronuclear multiple bond correlation (HMBC) experiments, as shown in Fig. 2. The COSY correlations revealed interactions from H-2 (*δ*_H_ 8.18) to H-4 (*δ*_H_ 8.23) through H-3 (*δ*_H_ 7.95), H-6 (*δ*_H_ 7.97) to H-8 (*δ*_H_ 8.06) through H-7 (*δ*_H_ 7.96), and between H-12 (*δ*_H_ 5.97) and H_3_-13 (*δ*_H_ 1.90), indicating the presence of 5-ethylidenephenazine-1-yl moiety. Key HMBC correlations provided further structural insights. The coupling of H_3_-13 to C-5 (*δ*_C_ 143.4), of H-12 to C-5, C-6 (*δ*_C_ 129.1), and C-10a (*δ*_C_ 141.6), as well as the interaction of the methoxy protons (*δ*_H_ 3.98) and H-2 with C-11 (*δ*_C_ 166.8), were indicative of a methyl 5-ethylidenephenazine-1-carboxylate moiety, akin to that found in methyl saphenate [17, 18]. Additionally, the ^1^H and ^13^C-NMR spectra hinted at the presence of an 8'-substituted genistein moiety within compound **1**. This was further corroborated by crucial HMBC correlations, including interactions from proton H-2' (*δ*_H_ 8.44) to C-4' (*δ*_C_ 180.3), C-8'a (*δ*_C_ 155.4), and C-1'' (*δ*_C_ 121.8). Moreover, the proton of the 5'-OH group (*δ*_H_ 13.02) exhibited correlations to C-4'a (*δ*_C_ 104.1), C-5' (*δ*_C_ 159.9), and C-6' (*δ*_C_ 99.4). H-6' (*δ*_H_ 6.20) also correlated with C-4'a and C-8' (*δ*_C_ 110.1). The observed ^1^H-^1^H COSY correlations between H-3''/5'' (*δ*_H_ 6.82) and H-2''/6'' (*δ*_H_ 7.39) further supported the structure of the genistein moiety. The attachment of the genistein structure to the phenazine core was confirmed through HMBC correlations from H_3_-13 to C-8', and from H-12 to C-8', C-8'a, and C-7' (*δ*_C_ 164.4), indicating a single carbon–carbon bond connection between C-12 and C-8'. These NMR data and correlations provide compelling evidence for the structural integration of an 8'-substituted genistein moiety within the molecular architecture of the compound **1**, specifically methyl 5-(1-(genistein-8-yl)ethyl)-phenazine-1-carboxylate.

Phenazinoate B (**2**) was isolated as a racemic mixture and presented as a yellow powder. The molecular formula was determined to be C_22_H_19_O_3_N_3_, as indicated by the HRESIMS at *m*/*z* 374.1505 for the [M + H]^+^ ion. Analysis of one-dimensional (1D) and two-dimensional (2D) NMR spectra, as detailed in Table 1 and Fig. 2, revealed that the structure includes a methyl 12-deoxysaphenate unit, similar to the structural component identified in previously discussed compound **1**. The spin system delineated by the chemical shifts from H-3' (*δ*_H_ 6.64) to H-6' (*δ*_H_ 6.17), passing through H-4' (*δ*_H_ 6.31) and H-5' (*δ*_H_ 6.33) in sequence, indicated the presence of an *ortho*-disubstituted phenyl group within the molecular structure. Critical HMBC interactions were observed, notably NH-12 (*δ*_H_ 5.38) showed correlations to C-5 (*δ*_C_ 144.1), C-2' (*δ*_C_ 144.3), and C-6' (*δ*_C_ 110.9). Additionally, the hydroxy proton HO-2' (*δ*_H_ 9.40) showed correlations with C-1' (*δ*_C_ 136.1), C-2', and C-3' (*δ*_C_ 113.7). These HMBC correlations confirmed the presence of an *o*-aminophenol moiety, which was connected to the C-12 atom via the amino nitrogen. Consequently, the molecular structure of phenazinoate B (**2**) was elucidated as methyl 5-(1-(2-hydroxyphenylamino)ethyl)phenazine-1-carboxylate.

**Table 1:** ^1^H (500 MHz) and ^13^C (125 MHz) NMR data for **1** and** 2** in DMSO-*d*_6_

No.	**1**		**2**	
	*δ*_H_, mult. (*J* in Hz)	*δ*_C_, type	*δ*_H_, mult. (*J* in Hz)	*δ*_C_, type
1		131.2, C		131.5, C
2	8.18, dd (1.4, 6.9)	131.7, CH	8.25, d (6.9)	131.9, CH
3	7.95, dd (6.9, 8.7)	129.6, CH	8.03, dd (6.9, 8.8)	130.0, CH
4	8.23, dd (1.4, 8.7)	132.5, CH	8.50, d (8.8)	132.9, CH
4a		140.8, C		141.1, C
5		143.4, C		144.1, C
6	7.97, dd (2.2, 7.0)	129.1, CH	7.93, dd (3.2, 6.9)	127.3, CH
7	7.96, dd (7.0, 7.8)	131.7, CH	7.92, t (6.9)	131.9, CH
8	8.06, dd (2.2, 7.8)	127.3, CH	8.08, dd, (3.2, 6.9)	128.0, CH
8a		142.9, C		143.1, C
9a		139.3, C		139.8, C
10a		141.6, C		141.0, C
11		166.8, C		166.9, C
12	5.97, q (7.3)	29.2, CH	5.80, dq (6.7, 7.9)	47.6, CH
13	1.90, d (7.3)	18.5, CH_3_	1.68, d (6.7)	23.7, CH_3_
11-OCH_3_	3.98, s	52.5, CH_3_	4.00, s	52.7, CH_3_
12-NH			5.38, d (7.9)	
1'				136.1, C
2'	8.44, s	153.5, CH		144.3, C
3'		121.5, C	6.64, dd (2.1, 7.1)	113.7, C
4'		180.3, C	6.31, ddd (2.1, 7.1, 8.5)	116.2, CH
4'a		104.1, C		
5'		159.9, C	6.33, ddd (2.1, 7.1, 8.5)	119.6, CH
6'	6.20, s	99.4, C	6.17, dd (2.1, 7.1)	110.9, CH
7'		164.4^a^, C		
8'		110.1, C		
8'a		155.4, C		
2'-OH			9.40, s	
1''		121.8, C		
2''/6''	7.39, d (8.7)	130.2, CH		
3''/5''	6.82, d (8.7)	115.1, CH		
4''		157.3, C		
5'-OH	13.02, s			

^a^Confirmed by HMBC correlations of H-12 and H-6' to the carbon at *δ*_C_ 164.4

**Fig. 3:**
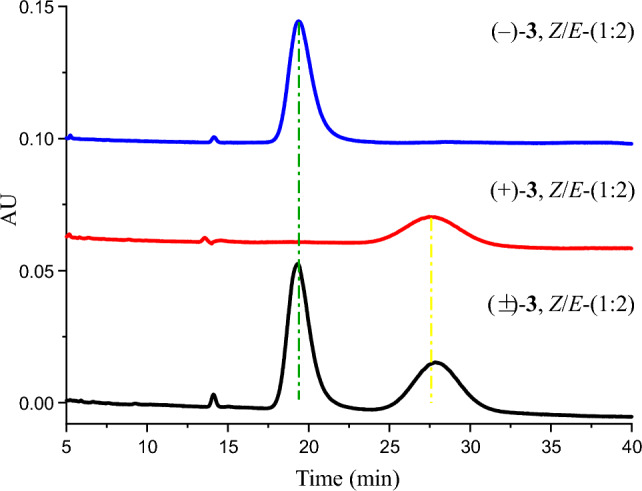
The mixture **3** was resolved into optically pure compounds (‒)-*Z*/*E*-**3** and ( +)-*Z*/*E*-**3** using HPLC with a ND(2)-RH chiral column (4.6 × 250 mm) eluted with 45% MeCN-H_2_O at 1 mL/min

**Fig. 4:**
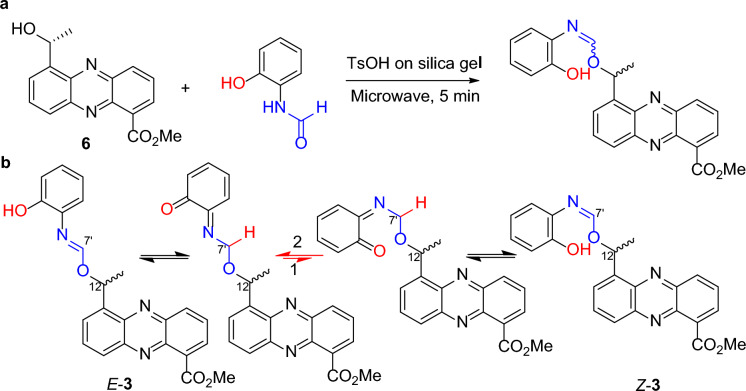
The synthesis of **3** from **6** and *o*-formamidophenol (**a**). Possible *Z*/*E*- dynamic equilibrium in solution via keto-enol tautomerization and single bond rotation at C-7'; this dynamic allows for the interconversion between the *Z* and *E* isomers, contributing to the complexity of their separation and characterization (**b**)

Phenazinoate C (**3**) was isolated as a yellow powder, presenting a complex mixture that is both racemic and an inseparable mix of *E*/*Z*-isomers. Its molecular formula was determined to be C_23_H_19_O_4_N_3_ based on the HRESIMS peak at *m*/*z* 402.1456 for [M + H]^+^ ion. When applying analytical high-performance liquid chromatography (HPLC) on an ND(2)-RH chiral column, it was feasible to separate compound **3** into enantiomerically pure entities, as illustrated in Fig. 3. The ^1^H-NMR and distortionless enhancement by polarization transfer including the detection of quaternary nuclei (DEPTQ) spectra for compound **3** revealed two distinct sets of signals, with an approximate ratio of 2:1 when measured in pyridine-*d*_5_. The structural elucidation of the major isomer within the mixture **3** began with the examination of its ^1^H-NMR spin systems, as depicted in Fig. 2. The identification of a methyl 5-(1-alkoxyethyl)-phenazine-1-carboxylate moiety was substantiated by delineating three distinct spin systems: one extending from H-2 (*δ*_H_ 8.21) to H-4 (*δ*_H_ 8.38), a second from H-6 (*δ*_H_ 8.09) to H-8 (*δ*_H_ 8.30), and a third connecting H-12 (*δ*_H_ 7.28) to H_3_-13 (*δ*_H_ 2.04). Additionally, pivotal HMBC interactions further clarified the structure, as illustrated in Fig. 2. Notable correlations including H_3_-13 to C-5 (*δ*_C_ 141.3), H-12 to both C-5 and C-6 (*δ*_C_ 129.7), as well as C-10a (*δ*_C_ 142.3). Correlations were also observed from both CH_3_O-11 (*δ*_H_ 4.04) and H-2 to C-11 (*δ*_C_ 167.9). The presence of an *o*-aminophenol moiety within the structure of compound **3** was suggested by a set of COSY correlations, which traced the continuous connectivity from H-3' (*δ*_H_7.16) to H-6' (*δ*_H_ 7.09). The structure was further elucidated by analyzing the HMBC cross-peaks. These revealed significant correlations from H-7' (*δ*_H_ 8.63) to C-12 (*δ*_C_ 51.0) and C-1' (*δ*_C_ 128.5), indicating the formation of a C = N bond between the 12-alkoxy group and the 1'-NH_2_ group. These findings provided a clearer understanding of the compound's chemical constitution, identified as methyl 5-(1-((2-hydroxyphenylimino)methoxy)ethyl)phenazine-1-carboxylate. The structural assignment of the minor isomer of compound **3** was deduced to be the geometric isomer of its major counterpart, owing to the resemblance of their 2D NMR correlations, as presented in Fig. 2. The principal distinction arose from the 1D NMR data, particularly around the C = N moiety, detailed in Table 2. This assignment was further corroborated by the synthetic approach that involved combining methyl (*R*)-saphenate with *o*-formamidophenol or *o*-hydroxyphenyl formamide, which also aligned with the structural identification conclusions presented in Fig. 4a. Moreover, the fact that the mixture **3** remained an inseparable *E*/*Z*-isomeric blend was rationalized by the presence of a dynamic equilibrium between the *E*- and *Z*-geometric isomers in solution. This equilibrium is facilitated by two keto-enol tautomerizations, along with unrestricted rotation around the C-7'–N single bond, as depicted in Fig. 4b. These findings elucidated the dynamic behavior of the isomers and the complexity of their separation.

**Table 2:** ^1^H (600 MHz) and ^13^C (150 MHz) NMR data for **3** in pyridine-*d*_6_

No.	**3** (*E*- isomer, major)		**3** (*Z*- isomer, minor)	
	*δ*_H_, mult. (*J* in Hz)	*δ*_C_, type	*δ*_H_, mult. (*J* in Hz)	*δ*_C_, type
1		133.0, C		133.1, C
2	8.21, dd (1.4, 6.9)	132.1, CH	8.23, dd (1.4, 6.9)	132.3, CH
3	7.76, dd (6.9, 8.7)	132.2, CH	7.77, dd (6.9, 8.7)	131.9, CH
4	8.38, dd (1.4, 8.7)	133.8, CH	8.38, dd (1.4, 8.7)	133.5, CH
4a		142.4, C		142.2, C
5		141.3, C		139.4, C
6	8.09, d (1.2, 6.9)	129.7, CH	7.64, d (6.9)	129.8, CH
7	7.77, dd (6.9, 8.7)	131.2, CH	7.72, dd (6.9, 8.7)	130.6, CH
8	8.30, dd (1.2, 8.7)	129.9, CH	8.34, dd (1.2, 8.7)	129.9, CH
8a		144.2, C		144.4, C
9a		141.1, C		141.2, C
10a		142.3 C		142.0, C
11		167.9, C		167.8, C
12	7.28, q (7.2)	51.0, CH	6.71, q (7.2)	52.8, CH
13	2.04, d (7.2)	19.1, CH_3_	1.93, d (7.2)	19.1, CH_3_
11-OCH_3_	4.04, s	52.9, CH_3_	4.05, s	52.9, CH_3_
1'		128.5, C		125.6, C
2'		156.6, C		156.4, C
3'	7.16, dd (1.4, 8.0)	117.5, CH	7.13, d (8.0)	117.6, CH
4'	7.24, ddd (1.7, 8.0, 9.0)	130.1, CH	7.24, ddd (1.7, 8.0, 9.0)	130.1, CH
5'	6.83, ddd (1.4, 7.5, 9.0)	119.7, CH	6.82, dd (7.8, 9.0)	119.5, CH
6'	7.09, dd (1.7, 7.5)	131.9, CH	7.09, dd (1.7, 7.8)	131.5, CH
7'	8.63, s	164.3, CH	9.38, s	164.4, CH
2'-OH	12.00, s		11.31, s

**Fig. 5:**
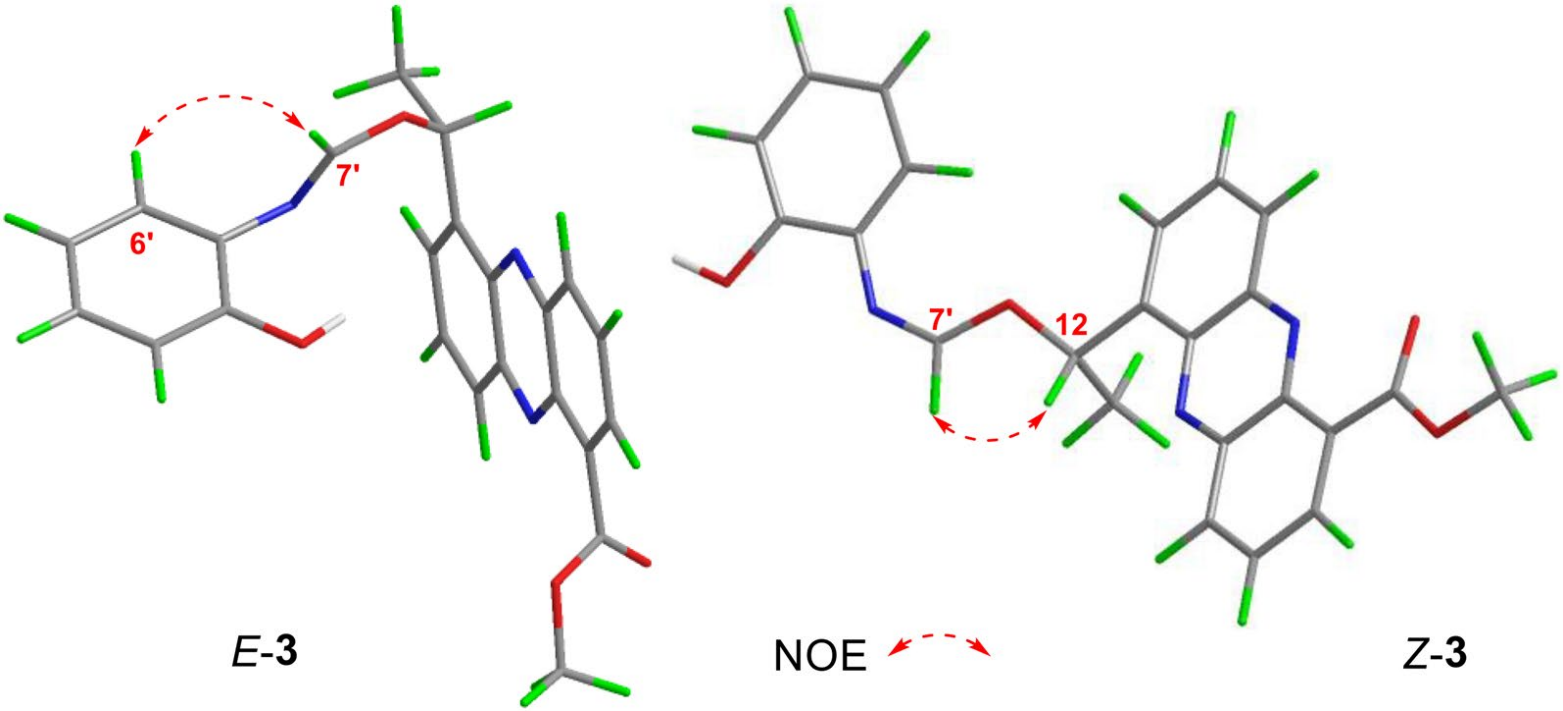
NOE correlations of *Z*-**3** and *E*-**3**

To ascertain the geometry of the C = N bond within the isomers of compound **3**, a Nuclear Overhauser Enhancement difference (NOEdiff) experiment was employed. The results, illustrated in Fig. 5, revealed distinct NOE correlations for each isomer. For the major geometric isomer of **3**, the NOE correlation observed between H-7' (*δ*_H_ 8.63) and H-6' (*δ*_H_ 7.09) allowed for the assignment of the *E*-geometry of the C = N bond Conversely, for the minor isomer of **3**, the selective irradiation of H-7' (*δ*_H_ 9.38) led to an enhancement observed at H-12 (*δ*_H_ 6.71), which indicated the *Z*-geometry of the C = N bond.

The racemic phenazinoate D (**4**) was isolated as a yellow powder. Its molecular formula, C_24_H_21_O_4_N_3_, was deduced from the HRESIMS peak at *m*/*z* 416.1604 [M + H]^+^ (calcd for C_24_H_21_N_3_O_4_^+^, 416.1605). The UV–Visible spectral data of the compound matched those of known phenazine analogs, indicating that **4** is a phenazine derivative. The inclusion of a methyl 5-(1-hydroxyethyl)-phenazine-1-carboxylate moiety was substantiated by pivotal 2D-NMR correlations, as depicted in Fig. 2. The structure features a 1',2',5'-trisubstituted phenyl ring, as evidenced by the COSY correlation of H-3' (*δ*_H_ 6.76) with H-4' (*δ*_H_ 7.01), as well as essential HMBC correlations. These include the coupling of H-3' to C-1' (*δ*_C_ 136.1) and C-5' (*δ*_C_ 126.2), H-4' to C-2' (*δ*_C_ 146.2) and C-6' (*δ*_C_ 121.7), and H-6' (*δ*_H_ 7.72) to both C-2' and C-4' (*δ*_C_ 123.8). Additionally, the pivotal HMBC signals connecting H-12 (*δ*_H_ 5.56) to C-1', C-2', and C-6' suggest that C-12 is directly bound to C-1' through a single C‒C bond. The ^1^H and ^13^C NMR data (Table 3) revealed the presence of an additional methyl group at *δ*_H/C_ 2.03/23.5 (CH_3_-7'), a carbonyl carbon at *δ*_C_ 169.0 (C-8'), and a phenolic hydroxy proton at *δ*_H_ 9.35. The HMBC correlations, specifically between H_3_-7' and C-8', confirmed the presence of an acetamide moiety within the structure. The chemical shifts observed for C-2' and C-5' allowed for the definitive placement of the hydroxy and acetamide groups on the molecular framework. Consequently, the structures of these compounds were conclusively established as methyl 5-(1-(2-hydroxy-5-acetamidophenyl)ethyl)phenazine-1-carboxylate.

**Table 3:** ^1^H and ^13^C NMR data for compounds **4** and **5** in DMSO-*d*_6_

No.	**4** (600, 150 MHz)		**5** (500, 125 MHz)	
	*δ*_H_, mult. (*J* in Hz)	*δ*_C_, type	*δ*_H_, mult. (*J* in Hz)	*δ*_C_, type
1		131.7, C		131.8, C
2	8.23, dd (1.4, 6.9)	131.4, CH	8.23, dd (1.4, 6.9)	131.3, CH
3	8.00, dd (6.9, 8.7)	129.8, CH	8.01, dd (6.9, 8.8)	129.9, CH
4	8.46, dd (1.4, 8.7)	132.9, CH	8.44, dd (1.4, 8.8)	132.7, CH
4a		141.2, C		141.1, C
5		145.8, C		143.3, C
6	7.77, d (7.0)	128.1, CH	8.20, d (6.8)	127.6, CH
7	7.96, dd (7.0, 8.7)	131.8, CH	8.03, dd (6.8, 8.8)	131.8, CH
8	8.07, dd (1.2, 8.7)	127.4, CH	8.14, dd (1.4, 8.8)	128.1, CH
8a		142.9, C		142.7, C
9a		139.6, C		139.6, C
10a		141.0, C		140.7, C
11		166.8, C		166.8, C
12	5.56, q (7.2)	36.7, CH	6.04, q (6.4)	70.2, CH
13	1.71, d (7.2)	21.5, CH_3_	1.55, d (6.4)	23.8, CH_3_
11-OCH_3_	3.99, s	52.6, CH_3_	4.00, s	52.6, CH_3_
1'		136.1, C	3.45, ddd (5.3, 11.1, 16.5)	61.6, CH_2_
			3.55, ddd (5.2, 11.4, 16.5)	
2'		146.2, C	3.37, dd (5.2, 5.3)	79.5, CH
3'	6.76, d (8.3)	115.9, CH	3.45, ddd (5.3, 11.1, 16.5)	60.8, CH_2_
			3.55, ddd (5.2, 11.4, 16.5)	
4'	7.01, dd (2.1, 8.4)	123.8, CH		
5'		126.2, C		
6'	7.72, d (2.1)	121.7, CH		
7'	2.03, s	23.5, CH_3_		
8'		169.0, C		
1'-OH			4.60, brs	
2'-OH	9.35, s			
3'-OH			4.60, brs	

The racemic mixture of phenazinoate E (**5**) was obtained as a yellow powder and determined to have the molecular formula C_19_H_20_O_5_N_2_, as indicated by a HRESIMS peak at *m*/*z* 357.1451 [M + H]^+^. The UV–Visible and ^1^H,^13^C-NMR spectral data for compound **5** suggested the presence of a methyl 5-(1-alkoxyethyl)phenazine-1-carboxylate moiety. The ^1^H-^1^H COSY correlations, as shown in Fig. 2, from HO-1' (*δ*_H_ 4.60) to HO-3' (*δ*_H_ 4.60) through H_2_-1' (*δ*_H_ 3.45/3.55), H-2' (*δ*_H_ 3.37) and H_2_-3' (*δ*_H_ 3.45/3.55) in sequence indicated a glycerin fragment, with substitution occurring at the 2'-hydroxy position. The connection between the glycerin and phenazine units was implied by an ether linkage between C-12 and C-2', supported by the HMBC correlation of H-2' to C-12 (*δ*_C_ 70.2). Consequently, the structure of compound **5** was conclusively identified as methyl 5-(1-((1,3-dihydroxypropan-2-yl)oxy)ethyl) phenazine-1-carboxylate.

**Fig. 6:**
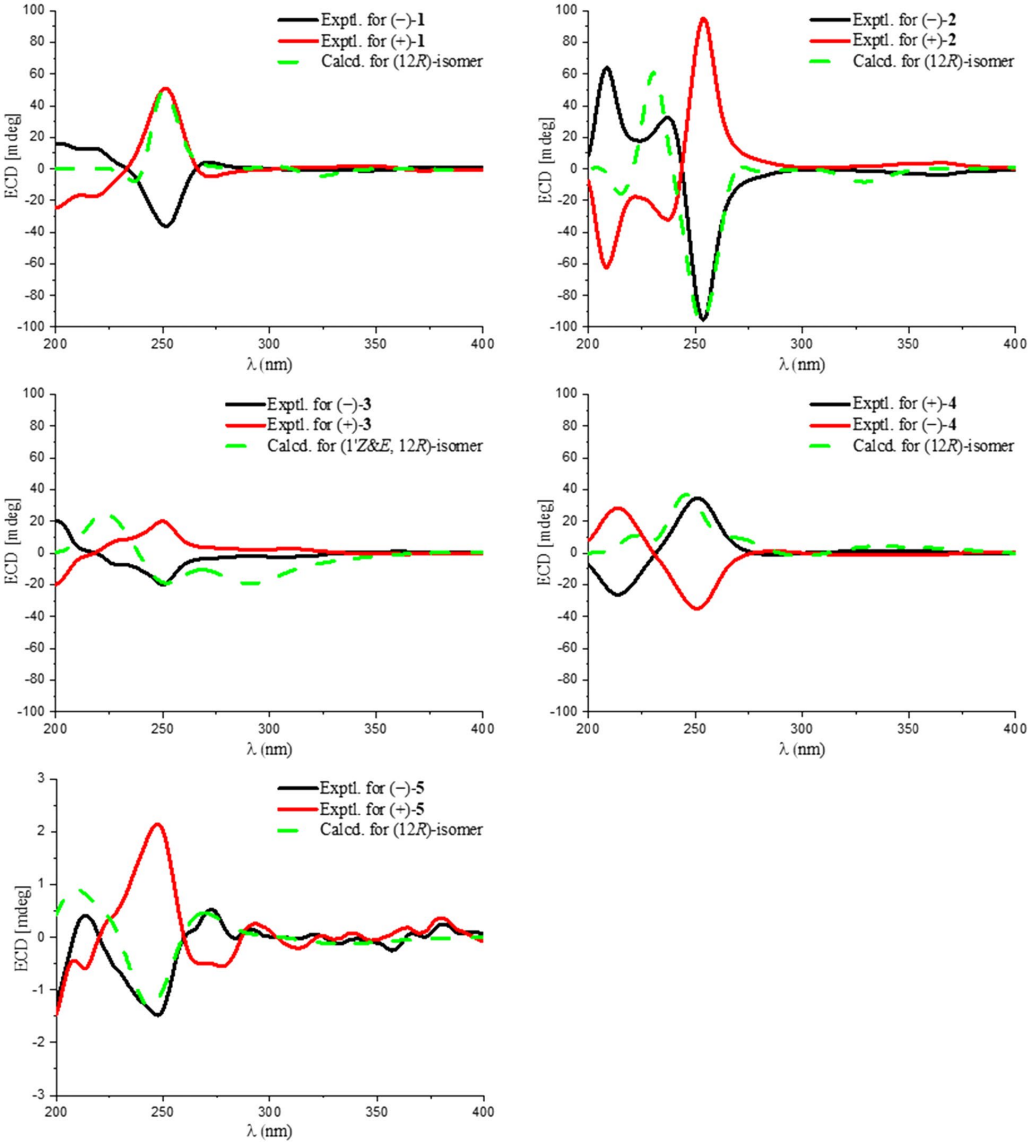
The experimental and calculated ECD curves of **1**‒**5**

The new compounds **1**–**5** were successfully resolved into their optically pure enantiomers using HPLC equipped with chiral columns. To determine the absolute configurations (ACs) of these ten compounds, the electronic circular dichroism (ECD) curves for the (*R*)-isomers were computed using time-dependent density functional theory (TDDFT) with the B3LYP/6–31G(d) basis set [19, 20], as detailed in the Supporting Information. The comparison of the experimental ECD curves with the calculated ones for compounds (+)-**1**, (−)-**2**, (−)-**3**, (−)-**4**, and (−)-**5** showed a good correlation with the curves of their respective (*R*)-isomers, as illustrated in Fig. 6. Consequently, the ACs of compounds (+)-**1**, (−)-**2**, (−)-**3**, ( +)-**4**, and (−)-**5** were assigned as (*R*)-, while the ACs of compounds (−)-**1**, ( +)-**2**, ( +)-**3**, (−)-**4**, and (+)-**5** were unambiguously determined to be (*S*)-.

### Physicochemical properties of 1–5

#### Phenazinoate A (1)

Yellow powder; UV (MeOH) *λ*_max_ (log *ε*) 253 (3.65), 365 (2.87) nm; ^1^H and ^13^C NMR data (Table 1); HRESIMS *m/z* 535.1502 [M + H]^+^ (calcd for C_31_H_23_N_2_O_7_, 535.1500). (−)-**1**, $\left[\alpha\right]^{28}_{\text {D}}$ −18.6 (*c* 0.1, MeOH); ECD (0.94 mM, MeOH) *λ*_max_ (Δ*ε*) 251 (−11.7), 271 (+1.4) nm. (+)-**1**:$\left[\alpha\right]^{28}_{\text {D}}$ +14.7 (*c* 0.1, MeOH); ECD (0.94 mM, MeOH)* λ*_max_ (Δ*ε*) 251 (+16.5), 272 (−1.6) nm.

#### Phenazinoate B (2)

Yellow powder; UV (MeOH) *λ*_max_ (log *ε*) 249 (2.93), 365 (2.17) nm; ^1^H and ^13^C NMR data (Table 1); HRESIMS *m/z* 374.1505 [M + H]^+^ (calcd for C_22_H_20_N_3_O_3_, 374.1499). (−)-**2**, $\left[\alpha\right]^{28}_{\text {D}}$ −40.9 (*c* 0.1, MeOH); ECD (1.3 mM, MeOH) *λ*_max_ (Δ*ε*) 209 (+14.4), 237 (+7.3), 254 (− 21.6) nm, 364 (−0.9). ( +)-**2**, $\left[\alpha\right]^{28}_{\text {D}}$ + 30.9 (*c* 0.1, MeOH); ECD (1.3 mM, MeOH) *λ*_max_ (Δ*ε*) 209 (−14.0), 238 (−7.3), 254 (+21.6), 364 (+ 0.9) nm.

#### Phenazinoate C (3)

Yellow powder; UV (MeOH) *λ*_max_ (log *ε*) 251 (3.69), 365 (2.99) nm; ^1^H and ^13^C NMR data (Table 2); HRESIMS *m/z* 402.1456 [M + H]^+^ (calcd for C_23_H_20_N_3_O_4_, 402.1488). (−)-**3**, $\left[\alpha\right]^{28}_{\text {D}}$ −37.1 (*c* 0.1, MeOH); ECD (1.2 mM, MeOH) *λ*_max_ (Δ*ε*) 229 (−1.9), 250 (−5.1), 306 (−0.6). (+)-**3**, $\left[\alpha\right]^{28}_{\text {D}}$ + 36.5 (*c* 0.1, MeOH); ECD (1.2 mM, MeOH) *λ*_max_ (Δ*ε*) 229 (+ 2.1), 251 (+ 5.0), 307 (+ 0.6) nm.

#### Phenazinoate D (4)

Yellow powder; UV (MeOH) *λ*_max_ (log *ε*) 253 (3.46), 365 (2.77) nm; ^1^H and ^13^C NMR data (Table 3); HRESIMS *m/z* 416.1604 [M + H]^+^ (calcd for C_24_H_21_N_3_O_4_, 416.1605). (−)-**4**, $\left[\alpha\right]^{28}_{\text {D}}$ −25.2 (*c* 0.1, MeOH); ECD (1.2 mM, MeOH) *λ*_max_ (Δ*ε*) 214 (+7.2), 251 (−8.8), 286 (+0.3) nm. (+)-**4**, $\left[\alpha\right]^{28}_{\text {D}}$ + 30.3 (*c* 0.1, MeOH); ECD (1.2 mM, MeOH) *λ*_max_ (Δ*ε*) 214 (−6.6), 251 (+8.7), 284 (−0.2) nm.

#### Phenazinoate E (5)

Yellow powder; UV (MeOH) *λ*_max_ (log *ε*) 252 (3.60), 366 (2.86) nm; ^1^H and ^13^C NMR data (Table 3); HRESIMS *m/z* 357.1451 [M + H]^+^ (calcd for C_19_H_21_N_2_O_5_, 357.1445). (−)-**5**, $\left[\alpha\right]^{28}_{\text {D}}$ −9.3 (*c* 0.1, MeOH); ECD (1.4 mM, MeOH) *λ*_max_ (Δ*ε*) 214 (+0.1), 248 (−0.3), 272 (+0.1) nm. (+)-**5**, [α]28 D + 9.6 (*c* 0.1, MeOH); ECD (1.4 mM, MeOH) *λ*_max_ (Δ*ε*) 246 (+ 0.5), 272 (−0.1) nm.

### Proposed biosynthetic pathway of compounds 1–5 and semi-synthetic production of compounds 1–3

**Fig. 7:**
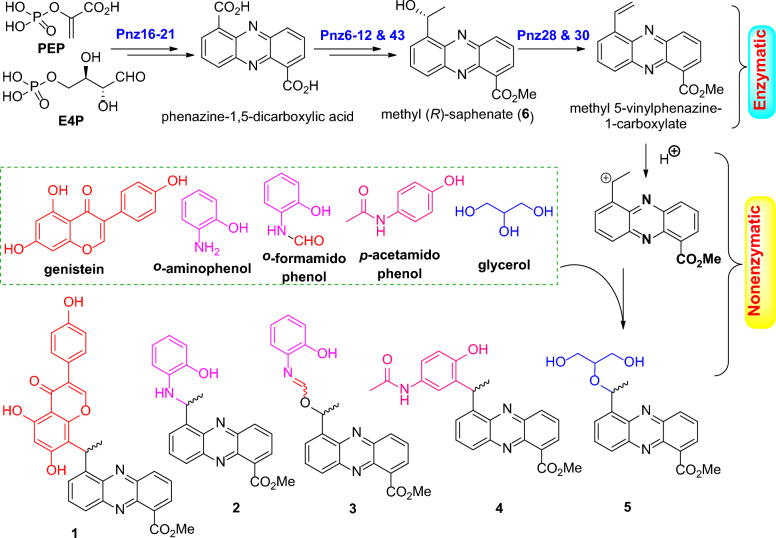
Proposed biosynthetic pathway of phenazinoates **1**‒**5** in *Streptomyces* sp. OUCMDZ-4923

Previously, we identified the enzymes Pnz16‒21 in *Streptomyces* sp. OUCMDZ-4923 as being responsible for the biosynthesis of the phenazine-1,5-dicarboxylic acid core, which is consequently converted into (*R*)-saphenic acid by enzymes Pnz6‒12 and then *O*-methylated to form methyl (*R*)-saphenate (**6**) by the methyltransferase Pnz43 [16]. We now propose that methyl 5-vinylphenazine-1-carboxylate, produced by the dehydration of methyl (*R*)-saphenate (**6**) under the action of enzymes Pnz28/30 [16], serves as a critical intermediate. This intermediate undergoes non-enzymatic reactions with compounds such as genistein, *o*-aminophenol, *o*-formamidophenol, *p*-acetamidophenol, and glycerol through a nonenzymatic pathway, leading to the formation of phenazinoates A–E (**1**‒**5**) (Fig. 7). In line with this hypothesis, compounds **1**‒**3** were successfully semi-synthesized from methyl (*R*)-saphenate (**6**) by reacting it with genistein, *o*-aminophenol, or *o*-formamidophenol. This transformation was achieved using *p*-toluenesulfonic acid absorbed on silica gel as a catalyst under microwave irradiation.

### Biological activity evaluation of compounds 1–5

The antibacterial efficacy of five pairs of enantiomerically pure compounds **1**–**5** was assessed as previously described [21]. The panel of test pathogens included four Gram-positive bacteria: *Staphylococcus aureus* ATCC 6538, methicillin-resistant *S*. *aureus* ATCC 43300 (MRSA), *Clostridium perfringens* CGMCC 1.0876, and *Bacillus subtilis* CGMCC 1.3376, along with two Gram-negative bacteria: *Pseudomonas aeruginosa* ATCC 10145 and *Escherichia coli* ATCC 11775. The minimum inhibitory concentrations (MICs), which are the lowest concentrations that inhibit microbial growth, were determined across a range of 25.0–0.78 μg/mL. Compounds **1** and **2** exhibited inhibitory activity against *B*. *subtilis*, *C*. *perfringens*, *S*. *aureus*, and MRSA, with MIC values ranging from 0.78 to 3.13 μg/mL, as detailed in Table 4. No antimicrobial activity was observed for the other tested bacteria, and compounds **3**–**5** did not exhibit inhibition against any of the test pathogens at the maximum concentration tested, which was 25.0 μg/mL. Additionally, it was noted that there was no difference in antibacterial activity between the two enantiomers. The results indicated that conjugating isoflavone or *o*-aminophenol moieties to the phenazine core can enhance the antibacterial efficacy of phenazine conjugates. However, both the *Levo*- and *Dextro*- enantiomers demonstrated the same level of efficacy against the four tested Gram-positive bacteria, suggesting that the chirality of these compounds does not influence their antibacterial activity. This observation could be explained by the possibility that the chiral center of the drug molecule may not located at the core position of the pharmacophore, or that the receptor's binding pocket is sufficiently spacious or flexible to accommodate either enantiomer, resulting in an equivalent biological effect.

**Table 4:** The MICs of compounds **1** and **2** against bacteria (*μ*g/mL)^a^

Compounds	*S. aureus*	MRSA	*B. subtilis*	*C. perfringens*
(−)-**1**	3.13	1.56	0.78	0.78
(+)-**1**	3.13	1.56	0.78	0.78
(−)-**2**	6.25	12.5	6.25	12.5
(+)-**2**	6.25	12.5	6.25	12.5
Cip^b^	0.098	0.39	0.012	0.049

^a^The MICs for compounds **3**–**5** against *B*. *subtilis*, *C*. *perfringens*, *S*. *aureus*, MRSA, *P*. *aeruginosa*, and* E*. *coli* as well as those for compounds** 1** and** 2** against *P*. *aeruginosa*, and* E*. *coli*, were all greater than 25.0 μg/mL

^b^Ciprofloxacin hydrochloride, used as the positive control, exhibited MICs of 0.049* μg*/mL against *P*. *aeruginosa* and 0.0061 μg/mL against *E*. *coli*, respectively

## Conclusion

In summary, five novel phenazine derivatives, phenazinoates A–E (**1**–**5**), were isolated and characterized from the mangrove soil-derived *Streptomyces* sp. OUCMDZ-4923, representing five pairs of enantiomers. This marks the first study to report the conjugate of isoflavone, *o*-/*p*-aminophenol, or glycerol moieties to the phenazine core. Notably, compounds **1** and **2** which are conjugates of isoflavone or *o*-aminophenol and phenazine, exhibited antibacterial properties against a spectrum of four Gram-positive bacterial strains. Further insights were obtained from the analysis of biosynthetic pathways, leading to the successful semi-synthesis of phenazinoates A–C (**1**–**3**) from methyl (*R*)-saphenate using genistein, *o*-aminophenol, or *o*-acetamidophenol. This synthesis was facilitated by microwave-assisted solid acid catalysis, providing a method to produce these compounds in sufficient quantities for subsequent comprehensive studies on their antibacterial activities.

## Supplementary Information

Supplementary material 1.

## Data Availability

The datasets used or analyzed during the current study are available from the corresponding author on reasonable request.
