# Supplementary material for: Phenazinoates A‒E, five pairs of phenazine conjugates from a mangrove soil-derived Streptomyces strain OUCMDZ-4923
Source: Nat Prod Bioprospect. 2026 Mar 4;16(1):42. doi: 10.1007/s13659-026-00597-0 (PMC12957693; doi:10.1007/s13659-026-00597-0)

Supplementary material for

Phenazinoates A–E, Five Pairs of Phenazine Conjugates from a Mangrove Soil-derived *Streptomyces* strain OUCMDZ-4923

Dongyang Wang[1,3,4†], Peipei Liu[1†], Yukang Gao[1], Linmeng Chen[1], Liping Wang[3,4], Ning Li[5], and Weiming Zhu[1,2*]

[1] Key Laboratory of Marine Drugs, Ministry of Education of China, School of Medicine and Pharmacy, Ocean University of China, Qingdao 266003, China

[2] Laboratory for Marine Drugs and Bioproducts, Qingdao Marine Science and Technology Center, Qingdao 266237, China

[3] Natural Product Research Center of Guizhou Province, Guiyang 550014, China

[4] State Key Laboratory of Discovery and Utilization of Functional Components in Traditional Chinese Medicine, Guizhou Medical University, Guiyang 550014, China

[5] Biology Institute, Qilu University of Technology (Shandong Academy of Sciences), Engineering Research Center of Zebrafish Models for Human Diseases and Drug Screening of Shandong Province, Jinan 250103, China

[†] Contributed equally to this work

[*]Corresponding author: Weiming Zhu, E-mail: weimingzhu@ouc.edu.cn

List of Supporting Information

Experimental section S4
General experimental procedures S4
Actinomycete material S4
Extraction and isolation S4
Spectroscopic data of compound 6 .... S5
ECD calculations S5
Table S1. Stable conformers of (*R*)-**1**, (*R*)-**2** and (*R*)-*Z*/*E*-**3** S6
Table S2. Stable conformers of (*R*)-**4** and (*R*)-**5** S7
Figure S1. HRESIMS spectrum of **1** S9
Figure S2. ^{1}H-NMR spectrum (500 MHz) of **1** in DMSO-d_6 .... S10
Figure S3. ^{13}C-NMR spectrum (125 MHz) of **1** in DMSO-d_6 .... S111
Figure S4. ^{1}H-^{1}H COSY spectrum (500 MHz) of **1** in DMSO-d_6 .... S12
Figure S5. HSQC spectrum (500 MHz, 125 MHz) of **1** in DMSO-d_6 .... S13
Figure S6. HMBC spectrum (500 MHz, 125 MHz) of **1** in DMSO-d_6 .... S14
Figure S7. HRESIMS spectrum of **2** S15
Figure S8. ^{1}H-NMR spectrum (500 MHz) of **2** in DMSO-d_6 .... S16
Figure S9. ^{13}C-NMR spectrum (125 MHz) of **2** in DMSO-d_6 .... S17
Figure S10. ^{1}H-^{1}H COSY spectrum (500 MHz) of **2** in DMSO-d_6 .... S18
Figure S11. HSQC spectrum (500 MHz, 125 MHz) of **2** in DMSO-d_6 .... S19
Figure S12. HMBC spectrum (500 MHz, 125 MHz) of **2** in DMSO-d_6 .... S20
Figure S13. HRESIMS spectrum of **3** S21
Figure S14. ^{1}H-NMR spectrum (600 MHz) of **3** in pyridine-d_5 .... S22
Figure S15. DEPTQ spectra (150 MHz) of **3** in pyridine-d_5 .... S23
Figure S16. ^{1}H-NMR spectrum (600 MHz) of **3** in pyridine-d_5 .... S24
Figure S17. Amplified ^{1}H-NMR spectrum (600 MHz) of **3** in pyridine-d_5 .... S25
Figure S18. DEPTQ spectrum (150 MHz) of **3** in pyridine-d_5 .... S26
Figure S19. Amplified DEPTQ spectrum (150 MHz) of **3** in pyridine-d_5 .... S27
Figure S20. ^{1}H-^{1}H COSY spectrum (600 MHz) of **3** in pyridine-d_5 .... S28
Figure S21. HSQC spectrum (600 MHz, 150 MHz) of **3** in pyridine-d_5 .... S29
Figure S22. HMBC spectrum (600 MHz, 150 MHz) of **3** in pyridine-d_5 .... S30
Figure S23. NOEdiff spectrum (500 MHz) of **3** in pyridine-d_5 .... S31
Figure S24. HRESIMS spectrum of **4** S32
Figure S25. ^{1}H-NMR spectrum (600 MHz) of **4** in DMSO-d_6 .... S33
Figure S27. ^{1}H-^{1}H COSY spectrum (600 MHz) of **4** in DMSO-d_6 .... S35
Figure S28. HSQC spectrum (600 MHz, 150 MHz) of **4** in DMSO-d_6 .... S36
Figure S29. HMBC spectrum (600 MHz, 150 MHz) of **4** in DMSO-d_6 .... S37
Figure S30. HRESIMS spectrum of **5** S38
Figure S31. ^{1}H-NMR spectrum (500 MHz) of **5** in DMSO-d_6 .... S39
Figure S32. ^{13}C-NMR spectrum (125 MHz) of **5** in DMSO-d_6 .... S40
Figure S33. ^{1}H-^{1}H COSY spectrum (500 MHz) of **5** in DMSO-d_6 .... S41

Figure S34. HSQC spectrum (500 MHz, 125 MHz) of **5** in DMSO-d_6 .... S42
Figure S35. HMBC spectrum (500 MHz, 125 MHz) of **5** in DMSO-d_6 .... S43
Figure S36. ^{1}H-NMR spectrum (600 MHz) of synthetic **1** in DMSO-d_6 .... S44
Figure S37. ^{1}H-NMR spectrum (600 MHz) of synthetic **2** in DMSO-d_6 .... S45
Figure S38. ^{1}H-NMR spectrum (600 MHz) of natural and synthetic **3** in pyridine-d_5... S46

Experimental section

General experimental procedures

The separations were performed on the Waters HPLC system (1525 binary HPLC pump at 4 mL/min, 2487 dual λ absorbance detector). Semi-preparation column (ODS-A, 10×250 mm) was produced by YMC CO., Ltd. UV-Visible spectra were recorded on a NanoDrop One Microvolume UV-Vis Spectrophotometer. ORs data were collected using a JASCO P-1010 digital polarimeter. ECD spectra were measured on a JASCO J-715 spectropolarimeter. NMR spectroscopic data were recorded on a Varian System 500 spectrometer (500 MHz) or a JEOL JNM-ECP 600 spectrometer (600 MHz) in DMSO-d_6 ($\delta_{H/C}$ 2.50/39.52), Pyridine-d_5 ($\delta_{H/C}$ 7.22/123.87) or $CDCl_3$ ($\delta_{H/C}$ 7.26/77.16) solution. HR-ESI-TOF mass spectra were recorded using a Q-TOF ULTIMA GLOBAL GAA076 LC mass spectrometer. LC/ESI-MS data were measured using a Waters ACQUITY SQD II UPLC-DAD-MS system. The microwave reaction utilized an Midea M1-L213B microwave oven.

Actinomycete material

Streptomyces sp. OUCMDZ-4923 was isolated from a soil sample gathered around the root of the mangrove plant *Kandelia candel* and identified as a member of the genus *Streptomyces* through 16S rRNA gene sequencing (GenBank accession No. MW070530) [S1].

Extraction and isolation

Streptomyces sp. OUCMDZ-4923 was seeded in 400 conical flasks (500 mL) each containing 150 mL of medium (pH 8.0) that was prepared by dissolving glycerin (15 g), peptone (15 g), soluble starch (15 g), soybean meal (5 g), and $CaCO_3$ (2 g) in 1 L of natural seawater. The flasks were shaken for 5 days on a rotary shaker at 180 rpm at 28°C and then 60 liters of fermentation broth were harvested. The broth was extracted three times with equal volumes of EtOAc. The EtOAc was combined and evaporated under reduced pressure to afford 50 g dark oil as the EtOAc extract.

The above EtOAc extract was fractionated by Sephadex LH-20, eluting with MeOH-CH_2Cl_2 (1:1, *v/v*), to give four fractions (Fr.1−Fr.4). Fr.3 (2.0 g) was subjected to VLC on a silica gel column using step gradient of petroleum ether (PE) and EtOAc, starting from a ratio of 30:1 and gradually moving to pure EtOAc (0:1) to yield 11 subfractions (Fr.3.1−Fr.3.11). Subsequently, Fr3.2 (20.0 mg) was subjected to further purification by HPLC using an ODS-A column using 75% MeOH-H_2O as the mobile phase (4 mL/min) to yield compound **4** (6.8 mg, t_R 6.6 min) as a racemic mixture. The enantiopure compounds (+)-**4** (2.5 mg, t_R 7.8 min) and (–)-**4** (3.0 mg, t_R 8.7 min) were successfully isolated using a chiral MD column (4.6 × 250 mm) and a mobile phase consisting of *n*-hexane and isopropanol in a 75:25 ratio contained 0.5‰ triethylamine (*v/v*) at 1.0 mL/min. Compound **3** (6.0 mg, t_R 8.5 min) were isolated as a racemic mixture from Fr.3.9 (16.8 mg) by HPLC over an ODS-A column eluting with 50% MeCN-H_2O (4 mL/min). Enantiomerically pure compounds (–)-**3** (2.8 mg, t_R 19.7 min) and (+)-**3** (2.9 mg, t_R 26.7 min) (peak area ratio = 1:1) were obtained after a HPLC separation over a ND(2)-RH chiral column (4.6 × 250 mm) using MeCN-H_2O (45:55) as the mobile phase at 1.0 mL/min. Both compounds (–)-**3** and (+)-**3** are a pair of inseparable *Z*-/*E*- isomers. Fr.4 (1.5 g) was subjected to a VLC

separation using a step gradient elution with PE-EtOAc (30:1–0:1) to obtain 12 subfractions (Fr.4.1–Fr.4.12). Fr.4.4 (40.0 mg) was subjected to HPLC purification on a semi-preparative ODS-A column using 30% MeCN-H_2O with 0.05‰ trifluoroacetic acid (TFA) as the eluent at a flow rate of 4 mL/min. This process yielded compound **6** (108.8 mg, t_R 20.3 min). The racemic mixtures of **1** (4.0 mg) and **2** (3.2 mg) were obtained from Fr.4.5 (80.0 mg) by column chromatography over silica gel eluting with CH_2Cl_2-MeOH (50:1). The enantiomerically pure compounds (–)-**1** (1.9 mg, t_R 41.0 min) and (+)-**1** (1.7 mg, t_R 47.0 min) were obtained from a chiral HPLC separation on a Chiralpak IA column using *n*-hexane-isopropanol (85:15, *v*/*v*) as the eluent at a flow rate of 1 mL/min. The racemic mixture of compounds (–)-**2** (1.3 mg, t_R 23.2 min) and (+)-**2** (1.2 mg, t_R 24.4 min) was successfully separated into their enantiopure forms using HPLC on an INC chiral column (4.6 × 250 mm) by employing a mobile phase composed of *n*-hexane and isopropanol (80:20, *v*/*v*) with a flow rate of 1.0 mL/min. Fr.4.10 was subjected to HPLC over a Cholester column (10 × 250 mm) eluting with 63% MeOH-H_2O (4 mL/min) to yield compound **5** (7.3 mg, t_R 9.0 min). The enantiopure compounds (–)-**5** (3.2 mg, t_R 20.6 min) and (+)-**5** (3.1 mg, t_R 22.9 min) were then obtained by HPLC separation on a Chiralpak IA column using *n*-hexane-isopropanol (88:12) as the mobile phase at a flow rate of 1 mL/min.

Spectroscopic data of compound 6

Methyl (R)-saphenate (6): yellow powder; $[\alpha]_D^{28}$ +52.9 (*c* 0.1, MeOH); UV (MeOH) λ_{max} (log ε) 252 (4.67), 366 (3.96) nm; ^{1}H-NMR (500 MHz, $CDCl_3$): 1.80 (3H, d, *J* = 6.5 Hz, H-13), 4.11 (3H, s, 11-OCH_3), 4.88 (1H, d, *J* = 5.5 Hz, 12-OH), 5.71 (1H, qd, *J* = 6.5, 5.5 Hz, H-12), 7.78 (1H, d, *J* = 6.8 Hz, H-6), 7.82 (1H, dd, *J* = 8.7, 6.8 Hz, H-7), 7.87 (1H, dd, *J* = 8.7, 6.8 Hz, H-3), 8.24 (1H, d, *J* = 6.8 Hz, H-8), 8.26 (1H, d, *J* = 8.7 Hz, H-4), 8.36 (1H, d, *J* = 8.7 Hz, H-2); ^{13}C-NMR (125 MHz, $CDCl_3$): 23.8 (CH_3, C-13), 52.9 (CH_3,11-OCH_3), 68.9 (CH, C-12), 127.5 (CH, C-6), 129.4 (CH, C-3), 129.8 (CH, C-8), 131.0 (CH, C-7), 131.5 (C, C-1), 132.3 (CH, C-2), 133.4 (CH, C-4), 140.9 (C, C-9a), 141.8 (C, C-10a), 142.7 (C, C-8a), 144.3 (C, C-4a), 167.1 (C, C-11); ESIMS *m*/*z* 283.2 $[M + H]^+$.

ECD calculations

The calculations were performed by using the density functional theory (DFT) as carried out in the Gaussian 09 [S2]. The preliminary conformational distributions search was performed by HyperChem Release 8.0 software. All ground-state geometries were optimized at the B3LYP/6-31G(d) level in the Gaussian 09. Solvent effects of MeOH were evaluated at the same DFT level by using the SCRF/PCM method [S3]. The relative energies (kcal/mol) of all conformations were calculated, and the equilibrium populations was also calculated using the Boltzmann distribution (Table S1). The stable conformers (2 kcal/mol energy threshold) obtained were used to ECD calculation at the B3LYP/6-31G(d) level. The calculated ECD spectra were produced by SpecDis 1.70.1 software [S4].

Table S1. Stable conformers of (*R*)-**1**, (*R*)-**2** and (*R*)-*Z*/*E*-**3**

Conformer (Percent)	Conformation	Conformer (Percent)	Conformation	Conformer (Percent)	Conformation
(*R*)-**1a** (26.88%)	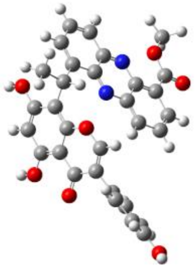	(*R*)-**2a** (27.1%)	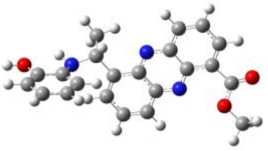	(*R*)-**3a** (32.4%)	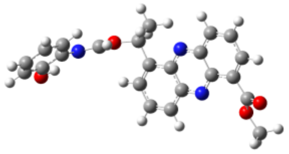
(*R*)-**1b** (20.71%)	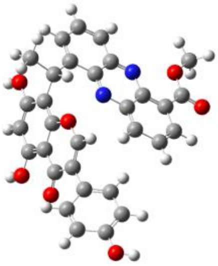	(*R*)-**2b** (26.8%)	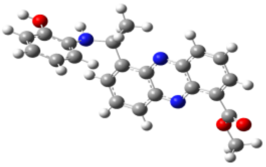	(*R*)-**3b** (30.3%)	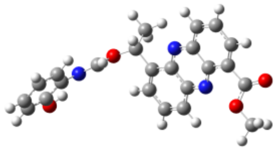
(*R*)-**1c** (20.67%)	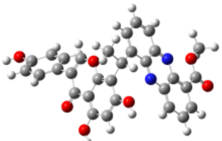	(*R*)-**2c** (26.8%)	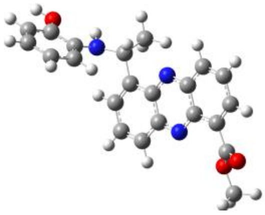	(*R*)-**3c** (30.3%)	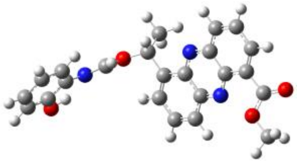
(*R*)-**1d** (16.48%)	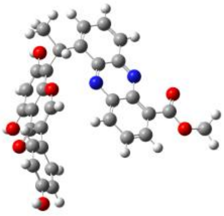	(*R*)-**2d** (9.9%)	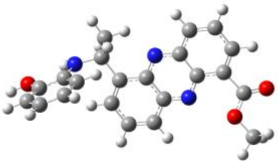	(*R*)-**3d** (2.4%)	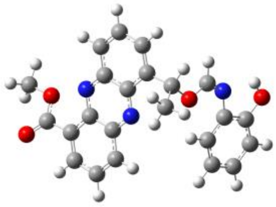
(*R*)-**1e** (15.26%)	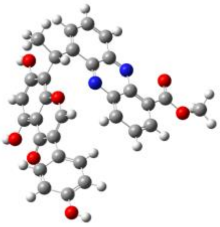	(*R*)-**2e** (9.4%)	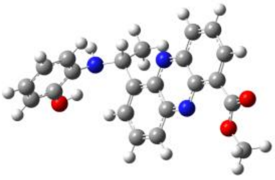	(*R*)-**3e** (2.4%)	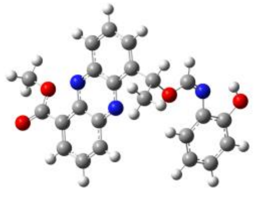
				(*R*)-**3f** (2.2%)	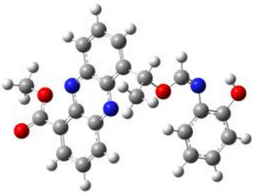

Table S2. Stable conformers of (*R*)-**4** and (*R*)-**5**

Conformer (Percent)	Conformation	Conformer (Percent)	Conformation
(*R*)-**4a** (100%)	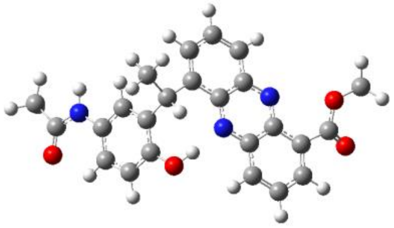	(*R*)-5**a** (51.1%)	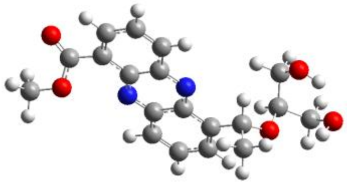
		(*R*)-5**b** (48.9%)	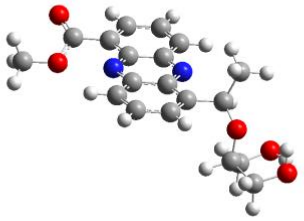

Synthesis of Phenazinoates A–C (1–3)

A solution was prepared by dissolving 50 mg of *p*-toluenesulfonic acid in 5 mL of water. This solution was then added to 450 mg of silica gel with a particle size of 100-200 mesh. The resulting mixture was subjected to vacuo evacuation to remove all traces of moisture, leaving it completely dry. Subsequently, the dried material was activated by heating at 110°C for 2 hours, yielding a solid acid catalyst.

To synthesize the target compounds, a solution was prepared by dissolving 20 mg (0.071 mmol) of methyl (*R*)-saphenate and 95.9 mg (0.355 mmol) of genistein (Adamas-beta® 99%) in 5 mL of acetone. This solution was then added to 200 mg of the previously prepared solid acid catalyst. After the addition, the acetone was evaporated under reduced pressure, leaving behind a residue. This residue was then heated in a microwave oven at a power setting of 350W for a duration of 5 min. Following the microwave treatment, the product was eluted with EtOAc (10 mL). The EtOAc eluent was subsequently washed thrice with water (10 mL) to remove any residual impurities. The EtOAc was then evaporated, resulting in a crude residue. This residue was purified using HPLC on an ODS-A column with 78% MeOH-H_2O as a mobile phase, yielding the racemic compound **1** (17.5 mg, t_R 15.2 min): ESIMS *m*/*z* 557.0 $[M + Na]^+$; ^{1}H-NMR (600 MHz, DMSO-d_6): see Figure S36.

Using similar procedures, the racemic compound **2** (13.1 mg, t_R 9.3 min) were synthsized from methyl (*R*)-saphenate (20 mg, 0.071 mmol) and *o*-aminophenol (38.7 mg, 0.355 mmol, Adamas-beta® 99%). The product was purified by HPLC on an ODS-A column, using a gradient elution of MeOH-H_2O (10-100%, 20 min). ESIMS *m*/*z* 374.1 $[M + H]^+$; ^{1}H-NMR (600 MHz, DMSO-d_6): see Figure S37.

To synthesize compound **3**, the *o*-formamidophenol was first obtained from *o*-aminophenol (Adamas-beta®, 97%) and formic acid (Adamas-beta®, 99%) following the method described by Hosseini-Sarvari M. et al [S5]. Subsequently, compound **3** was synthesized from methyl (*R*)-saphenate (20 mg, 0.071 mmol) and *o*-formamidophenol (48.6 mg, 0.355 mmol) through microwave-assisted solid acid catalysis as described earlier. Compound **3** (3.2 mg, t_R 11.2 min) was purified by HPLC on an ODS-A column, using a gradient elution of 40%-100% MeOH-H_2O (0-15 min). ESIMS *m*/*z* 402.1 $[M + H]^+$, 424.1 $[M + Na]^+$; ^{1}H-NMR (600 MHz,

pyridine-d_5): see Figure S38.

References:

[S1] Wang D, Liu P, Xia Y, Wang L, Li N, Zhu W. Antibacterial dimeric phenazine derivatives from a marine-derived *Streptomyces* sp. OUCMDZ-4923. *Mar Life Sci Tech* 2025; doi:10.1007/s42995-025-00328-3

[S2] Gaussian 09, Revision A.02, M. J. Frisch, G. W. Trucks, H. B. Schlegel, G. E. Scuseria, M. A. Robb, J. R. Cheeseman, G. Scalmani, V. Barone, B. Mennucci, G. A. Petersson, H. Nakatsuji, M. Caricato, X. Li, H. P. Hratchian, A. F. Izmaylov, J. Bloino, G. Zheng, J. L. Sonnenberg, M. Hada, M. Ehara, K. Toyota, R. Fukuda, J. Hasegawa, M. Ishida, T. Nakajima, Y. Honda, O. Kitao, H. Nakai, T. Vreven, J. A. Montgomery, Jr., J. E. Peralta, F. Ogliaro, M. Bearpark, J. J. Heyd, E. Brothers, K. N. Kudin, V. N. Staroverov, R. Kobayashi, J. Normand, K. Raghavachari, A. Rendell, J. C. Burant, S. S. Iyengar, J. Tomasi, M. Cossi, N. Rega, J. M. Millam, M. Klene, J. E. Knox, J. B. Cross, V. Bakken, C. Adamo, J. Jaramillo, R. Gomperts, R. E. Stratmann, O. Yazyev, A. J. Austin, R. Cammi, C. Pomelli, J. W. Ochterski, R. L. Martin, K. Morokuma, V. G. Zakrzewski, G. A. Voth, P. Salvador, J. J. Dannenberg, S. Dapprich, A. D. Daniels, O. Farkas, J. B. Foresman, J. V. Ortiz, J. Cioslowski, and D. J. Fox, Gaussian, Inc., Wallingford CT, 2009.

[S3] (a) Miertus, S.; Tomasi, J. *Chem. Phys.* 1982; 65: 239–245. doi:10.1016/0301-0104(82)85072-6. (b) Tomasi, J.; Persico, M. *Chem. Rev.* 1994; 94: 2027–2094. doi:10.1021/cr00031a013. (c) Cammi, R.; Tomasi, J. *J. Comp. Chem.* 1995; 16: 1449–1458. doi:10.1002/jcc.540161202

[S4] Bruhn, T.; Schaumlöffel, A.; Hemberger, Y.; Pescitelli, G. SpecDis, Version 1.70.1, Berlin, Germany, 2017, https://specdissoftware.jimdo.com.

[S5] Hosseini-Sarvari M, Sharghi H. ZnO as a new catalyst for N-formylation of amines under solvent-free conditions. J Org Chem. 2006; 71: 6652–6654. doi:10.1021/jo060847z

Figure S1. HRESIMS spectrum of **1**

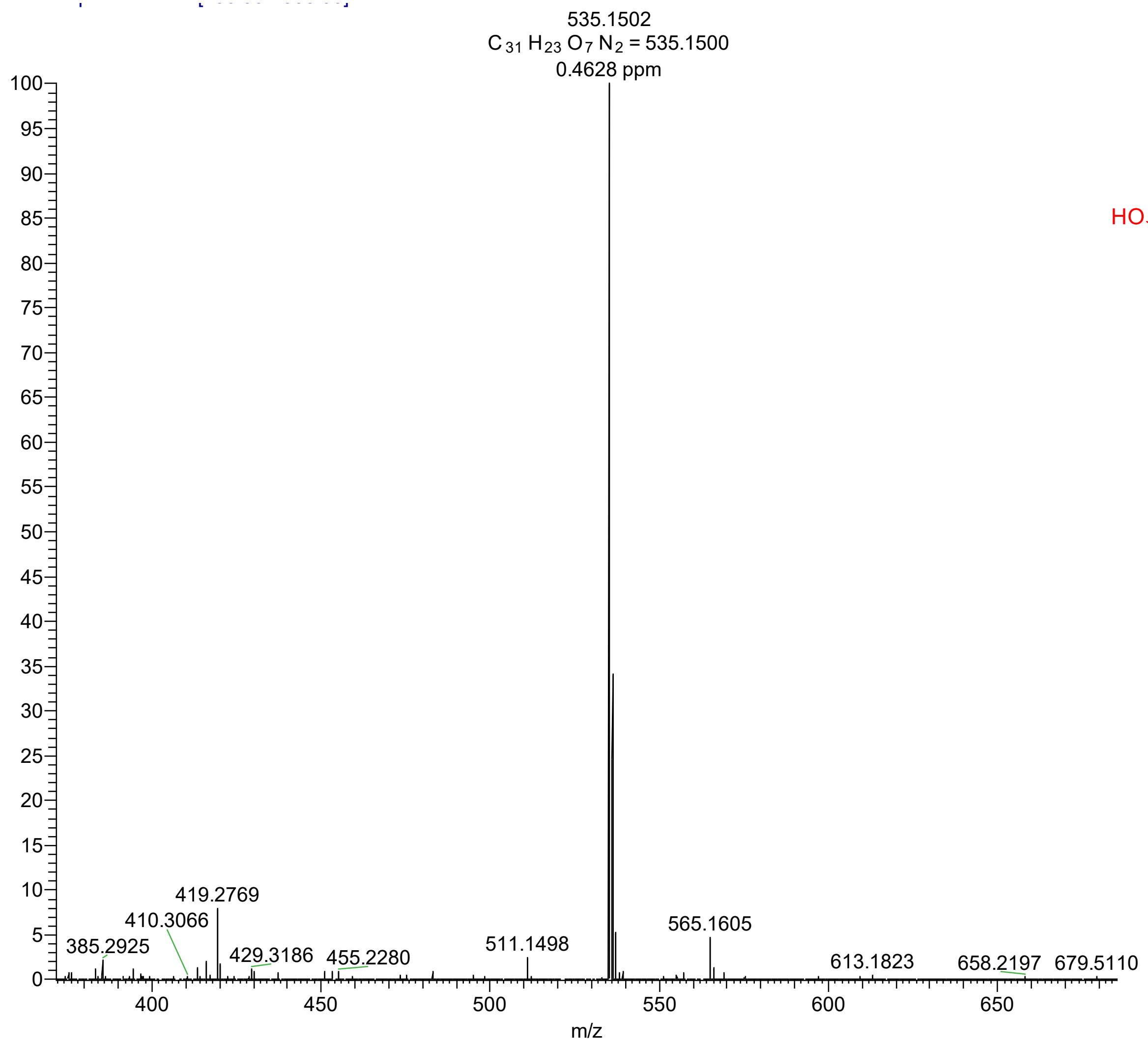

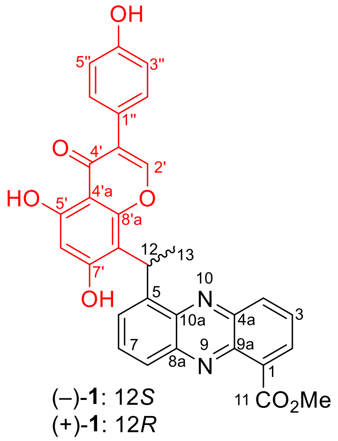

Figure S2. ^{1}H-NMR spectrum (500 MHz) of **1** in DMSO-d_6

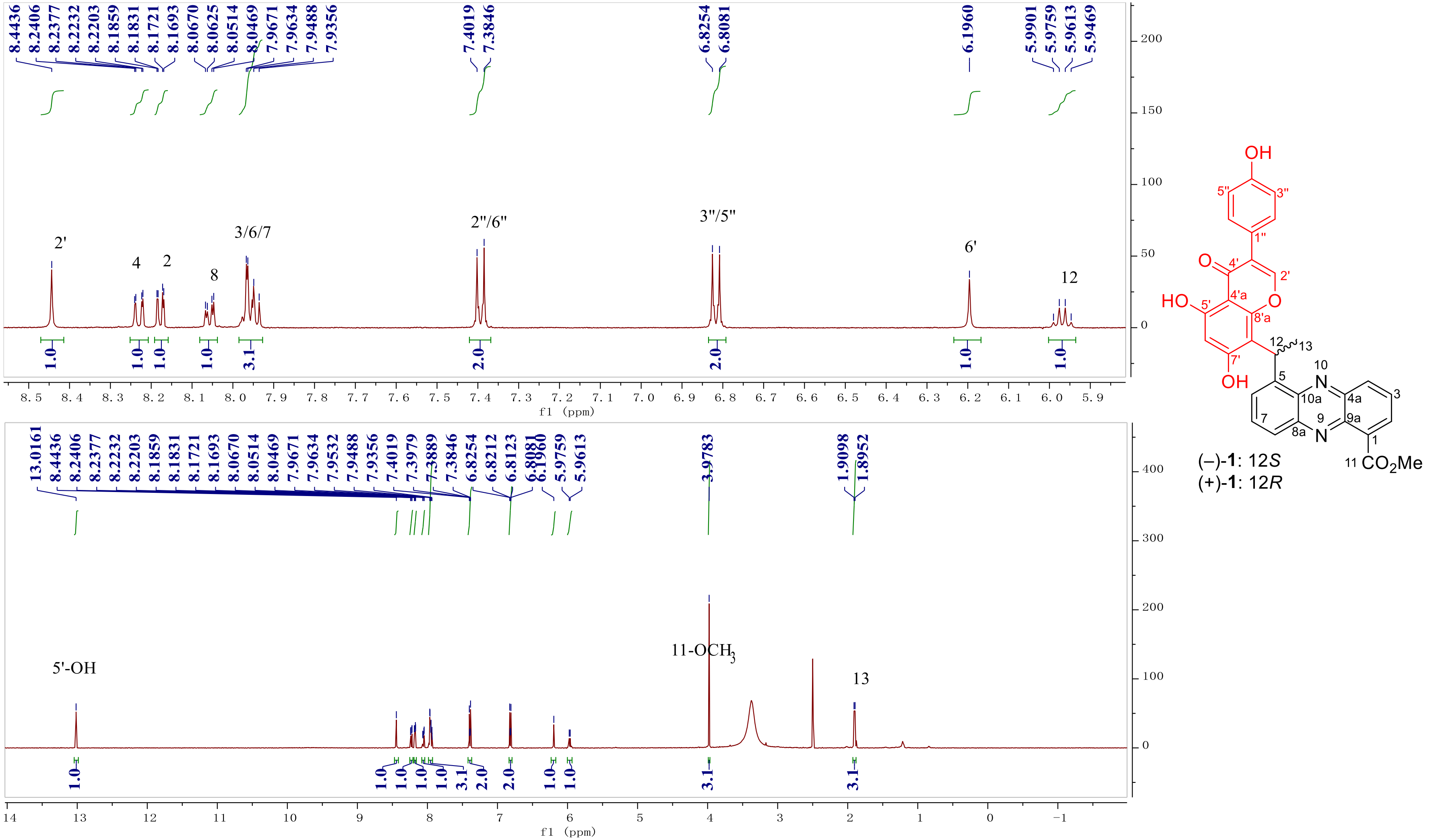

Figure S3. ^{13}C-NMR spectrum (125 MHz) of **1** in DMSO-d_6

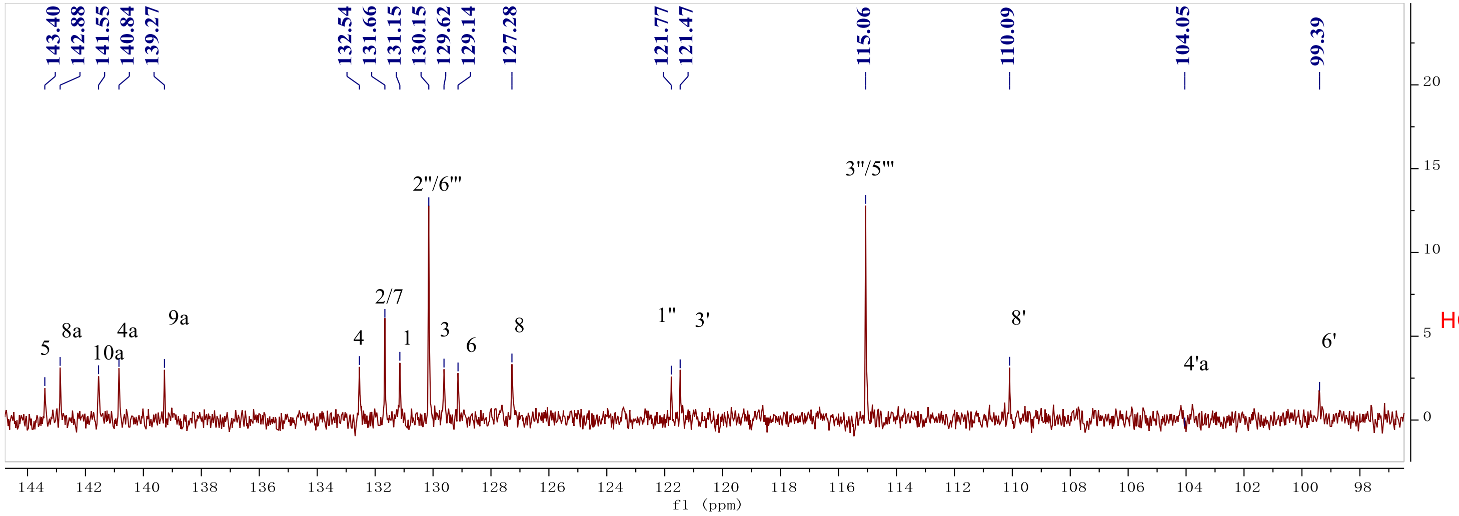

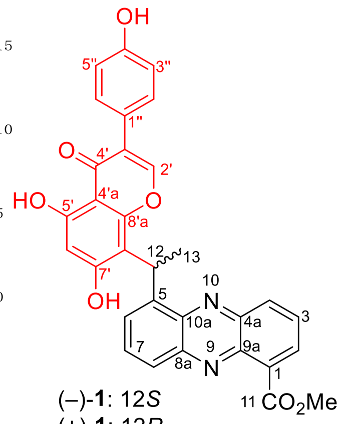

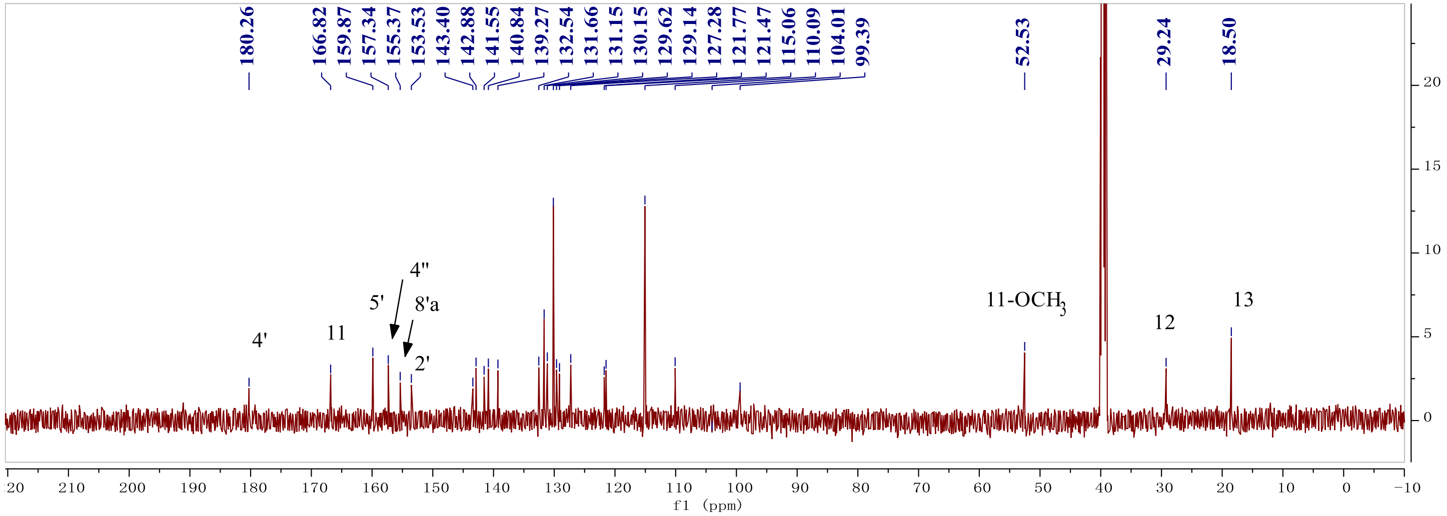

Figure S4. ^{1}H-^{1}H COSY spectrum (500 MHz) of **1** in DMSO-d_6

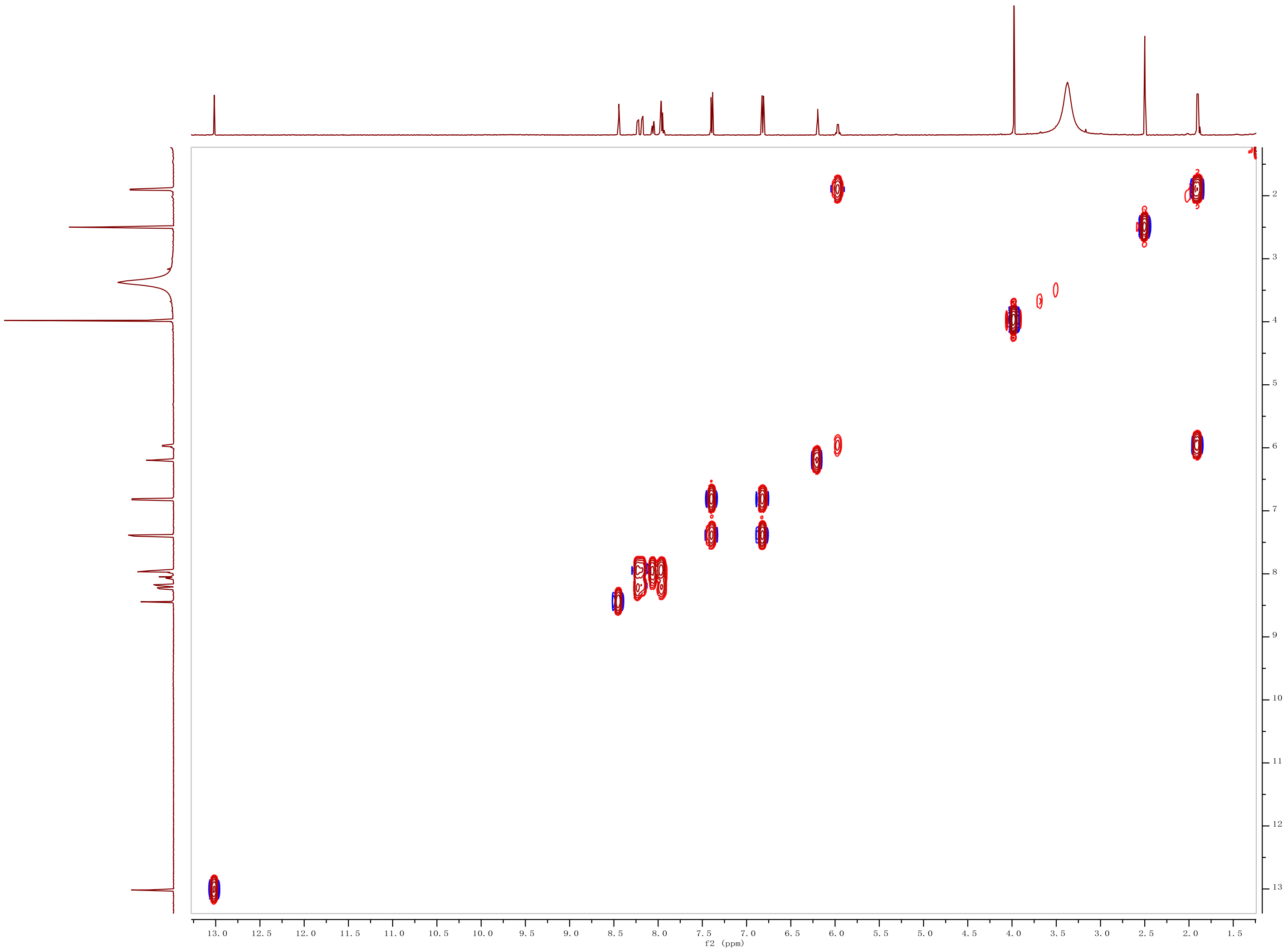

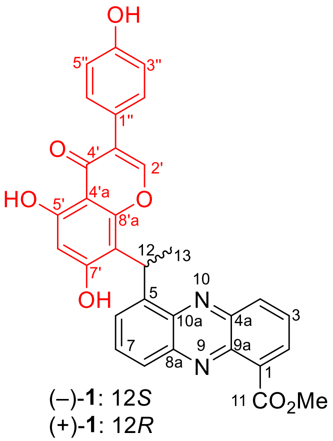

Figure S5. HSQC spectrum (500 MHz, 125 MHz) of **1** in DMSO-d_6

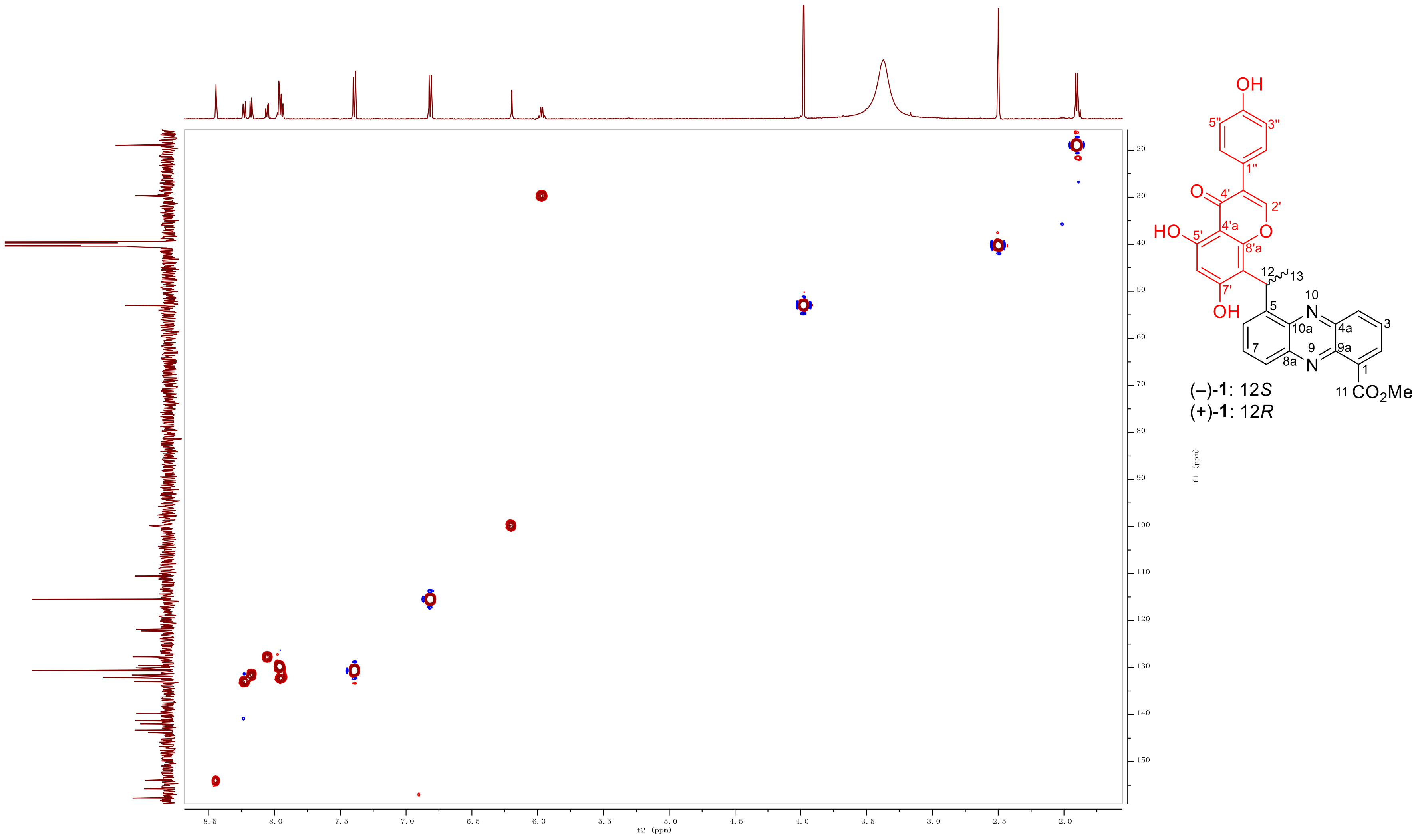

Figure S6. HMBC spectrum (500 MHz, 125 MHz) of **1** in DMSO-d_6

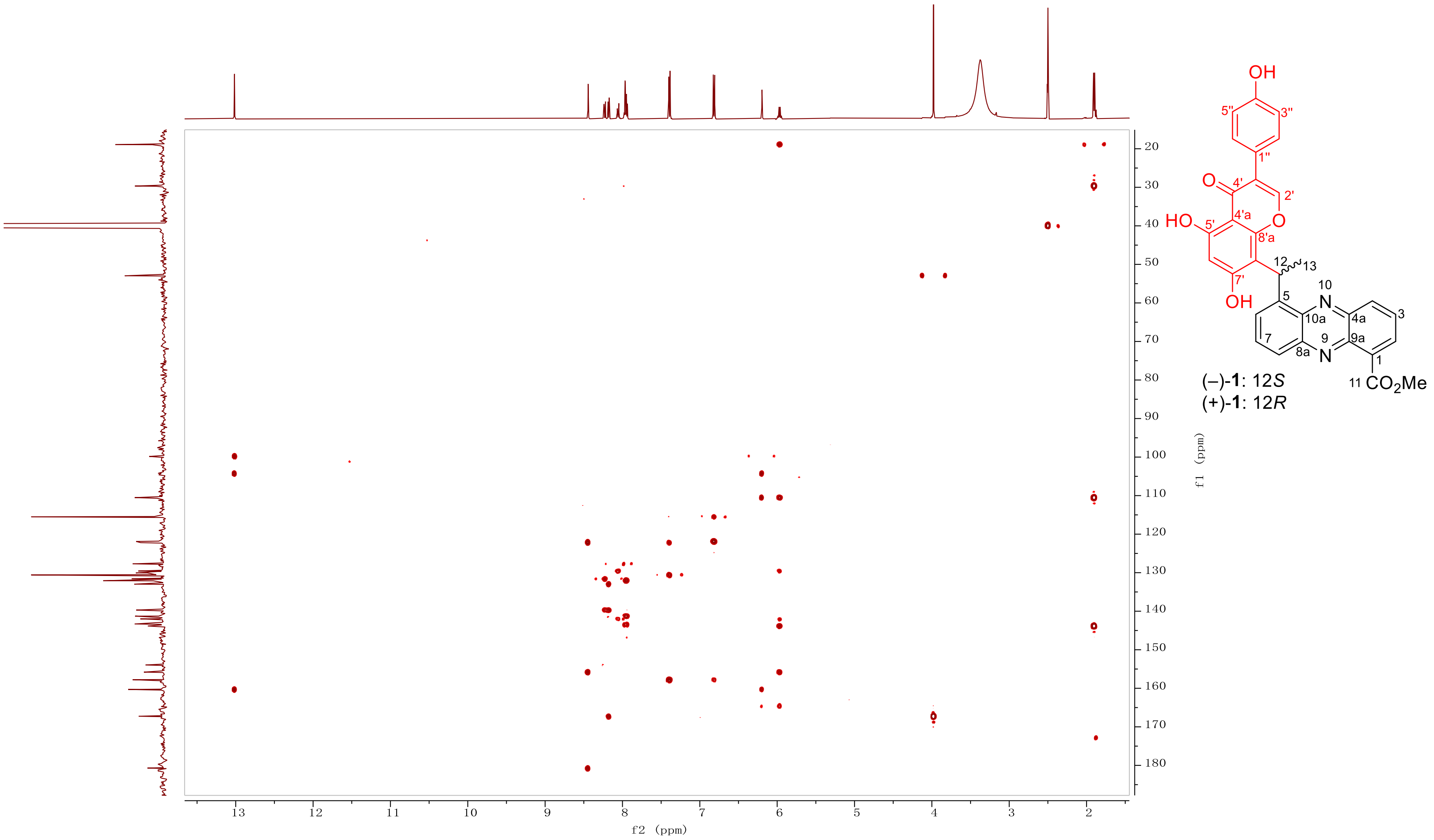

Figure S7. HRESIMS spectrum of **2**

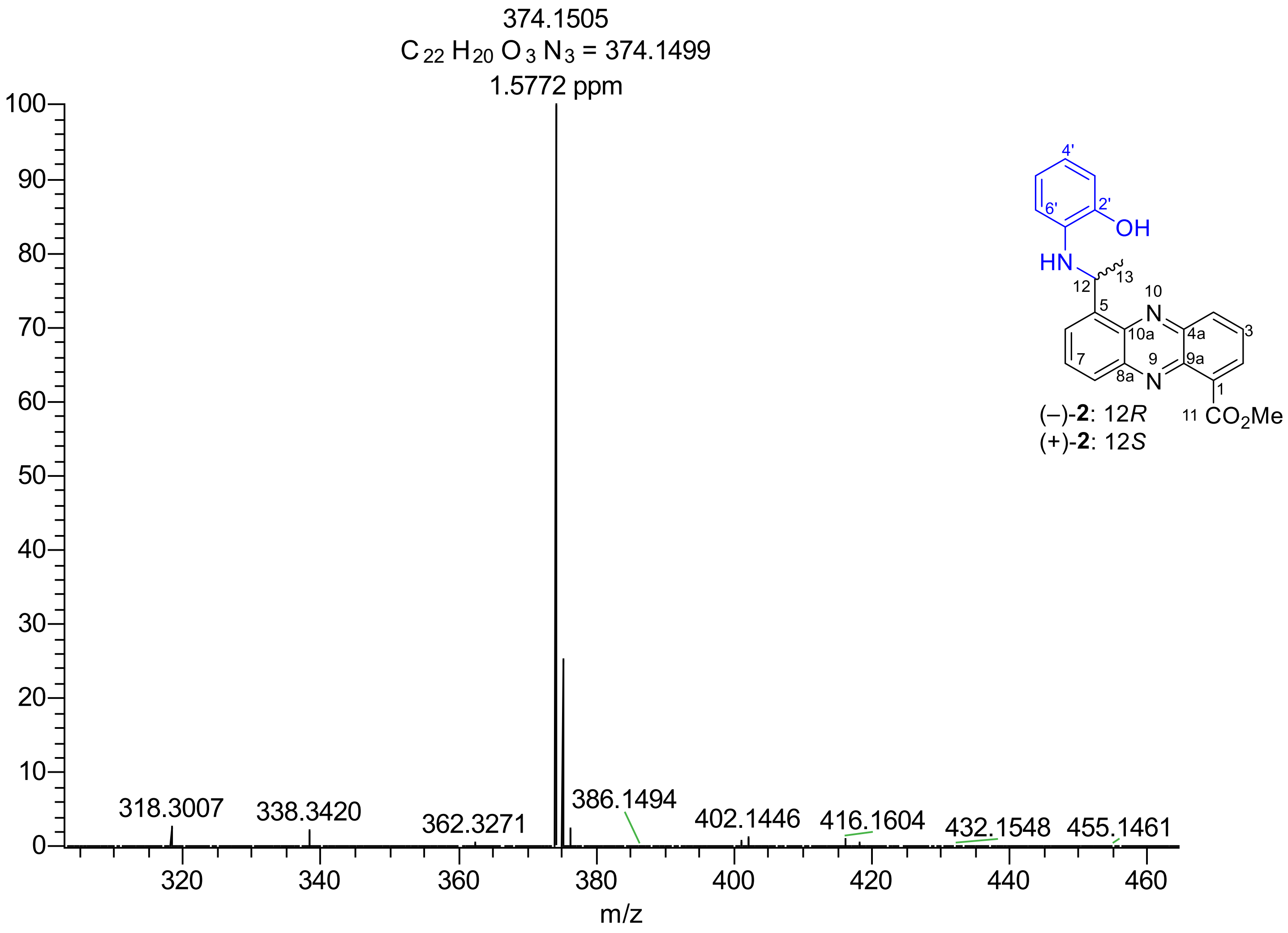

Figure S8. ^{1}H-NMR spectrum (500 MHz) of **2** in DMSO-d_6

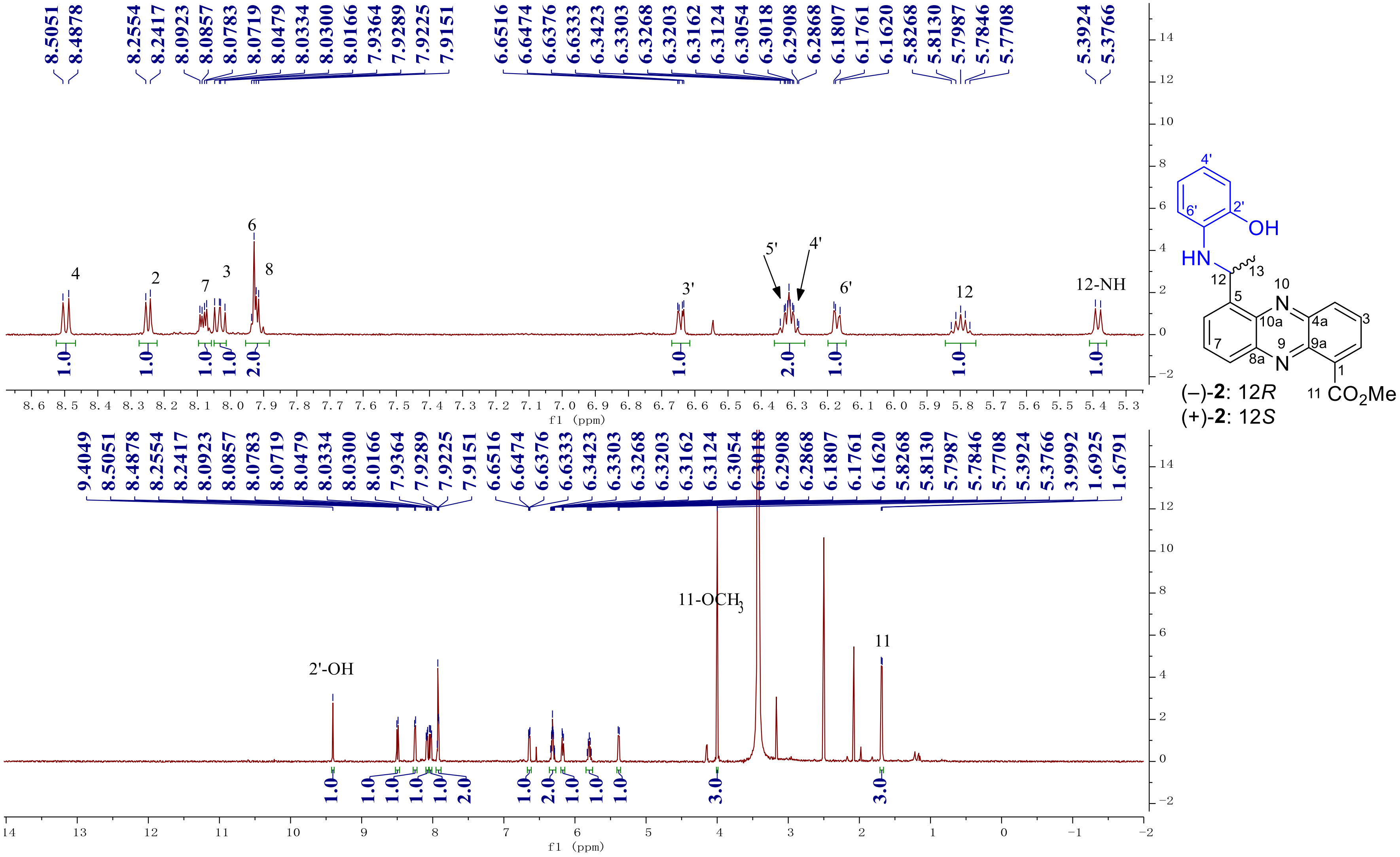

Figure S9. ^{13}C-NMR spectrum (125 MHz) of **2** in DMSO-d_6

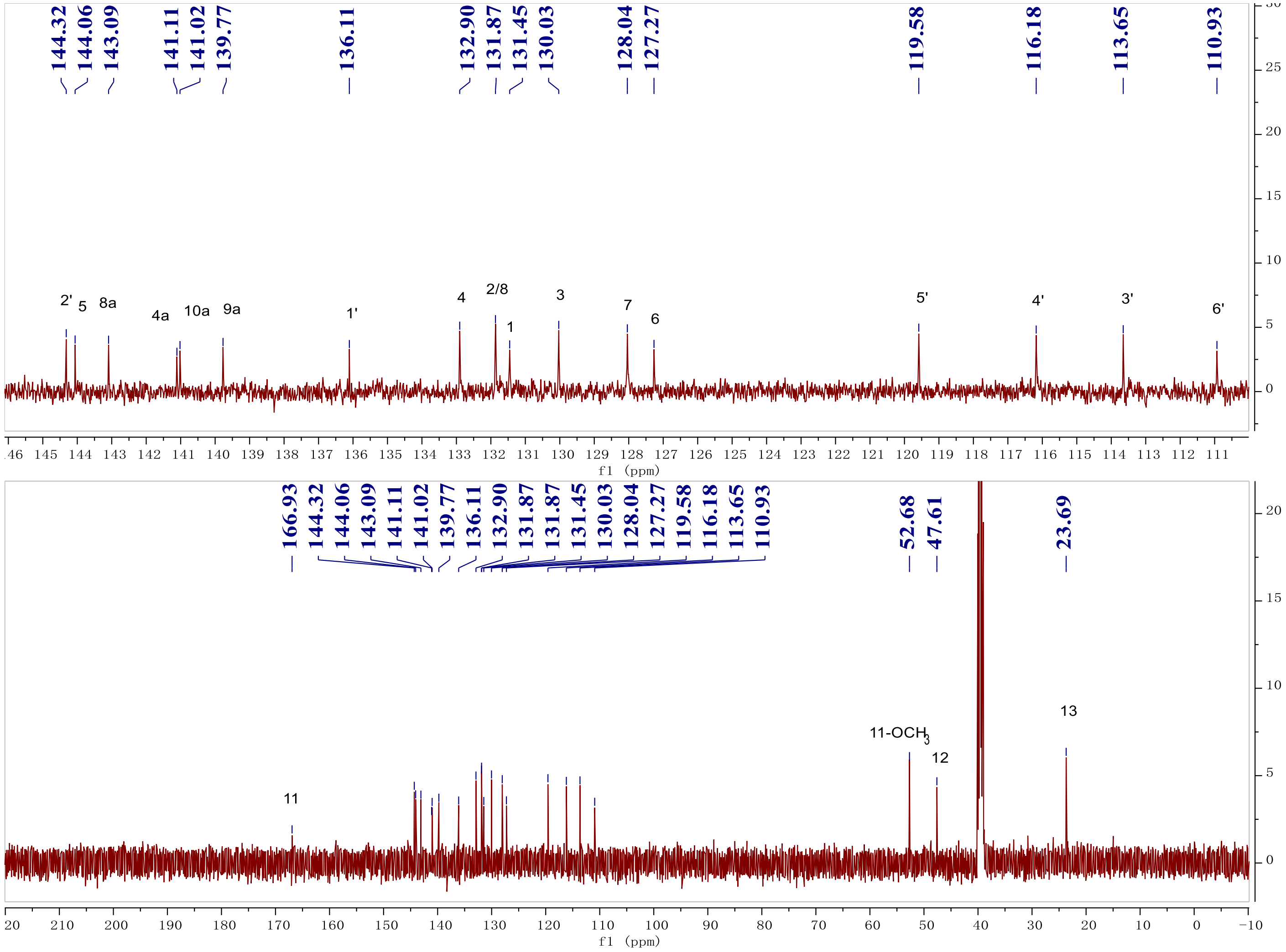

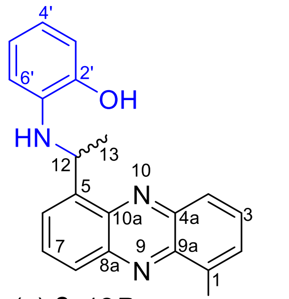

(–)-**2**: 12*R*
(+)-**2**: 12*S*

Figure S10. ^{1}H-^{1}H COSY spectrum (500 MHz) of **2** in DMSO-d_6

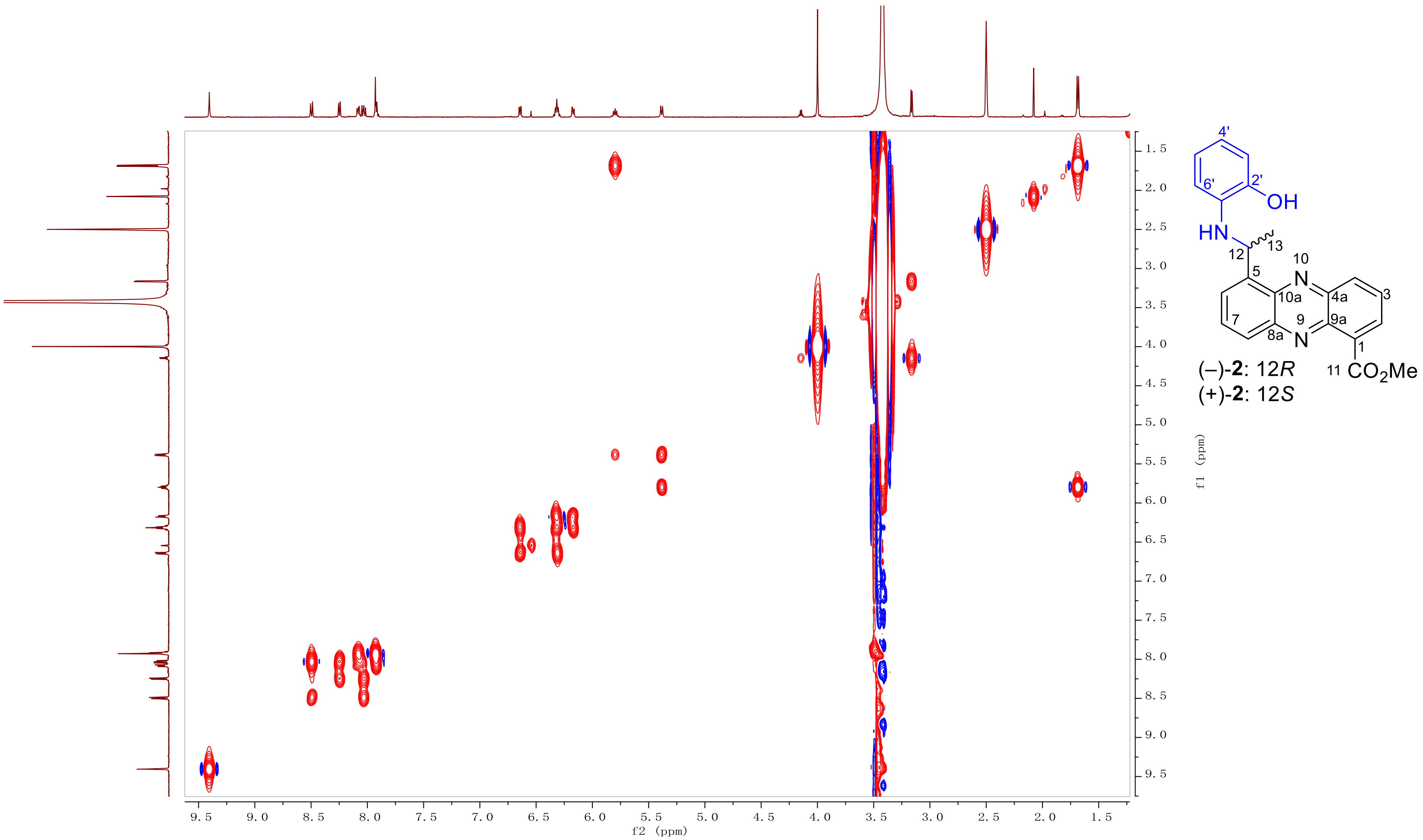

Figure S11. HSQC spectrum (500 MHz, 125 MHz) of **2** in DMSO-d_6

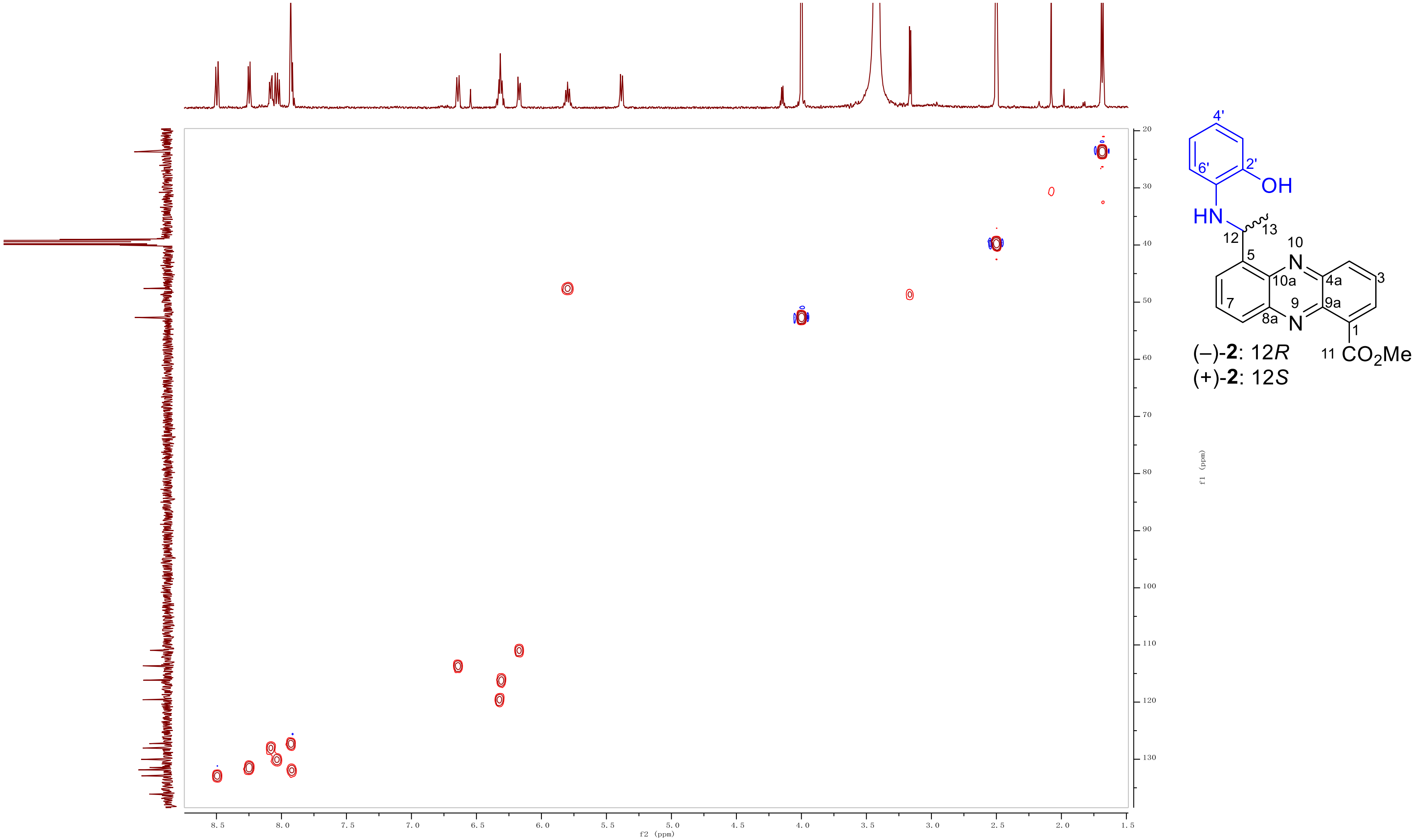

Figure S12. HMBC spectrum (500 MHz, 125 MHz) of **2** in DMSO-d_6

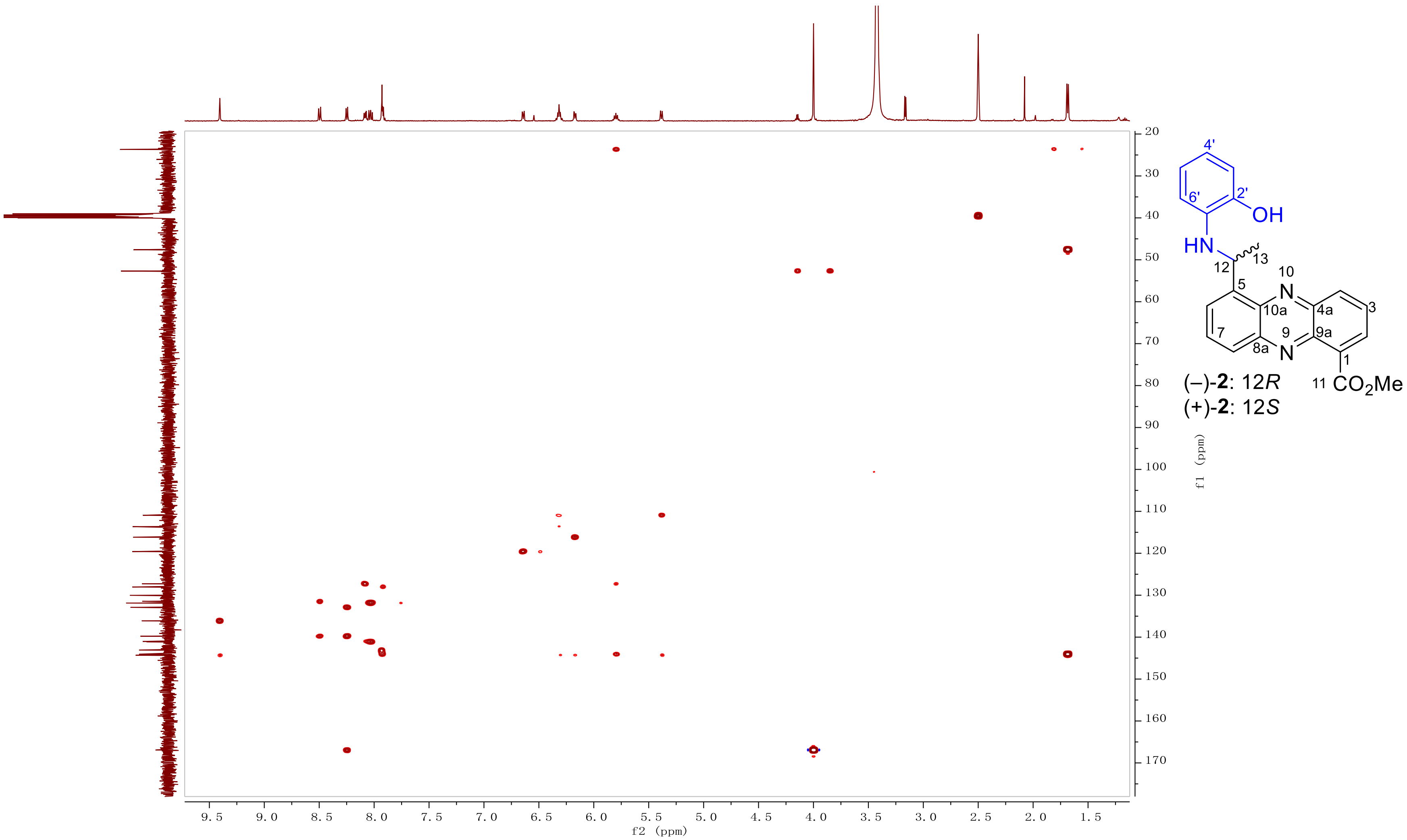

Figure S13. HRESIMS spectrum of **3**

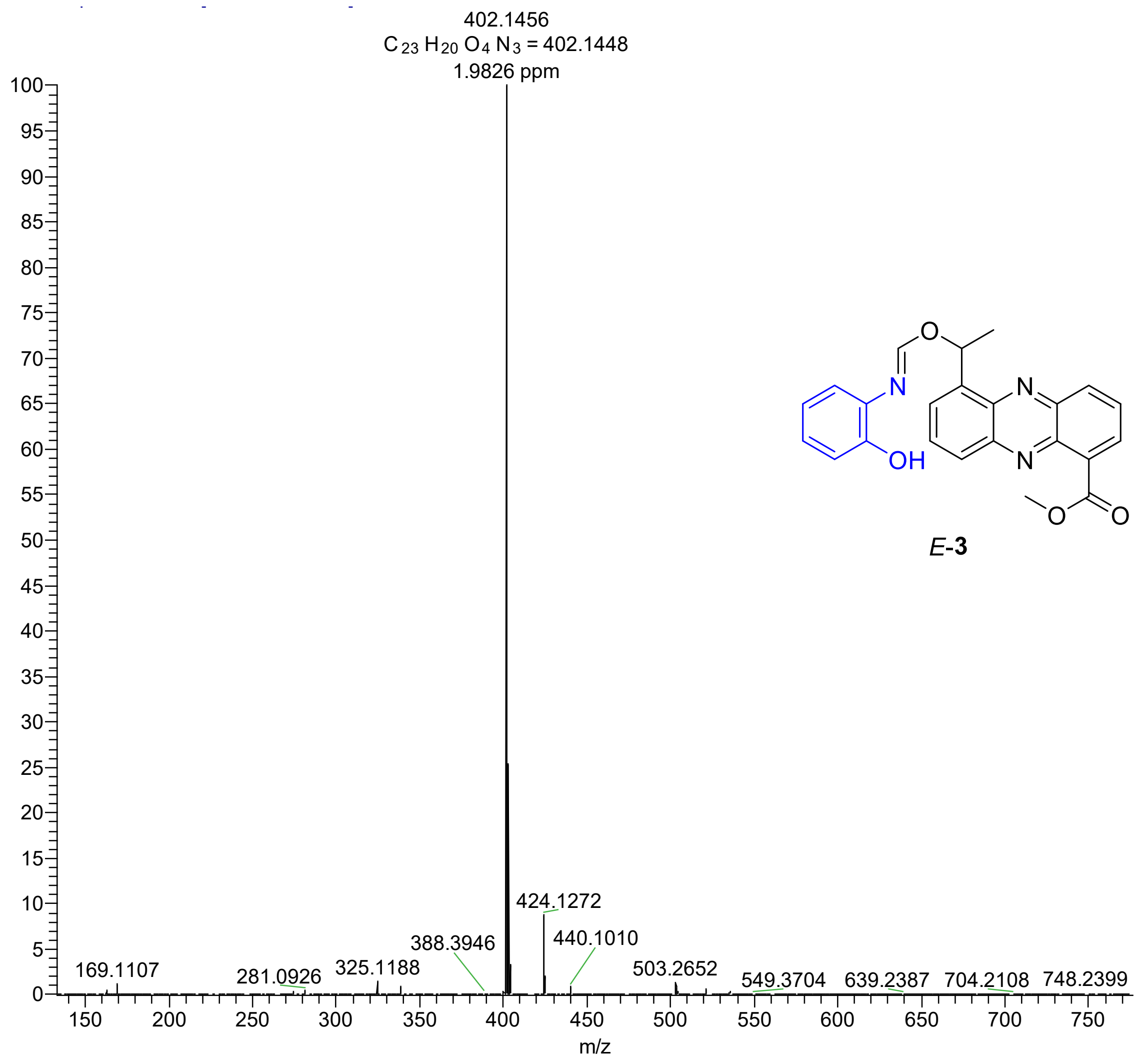

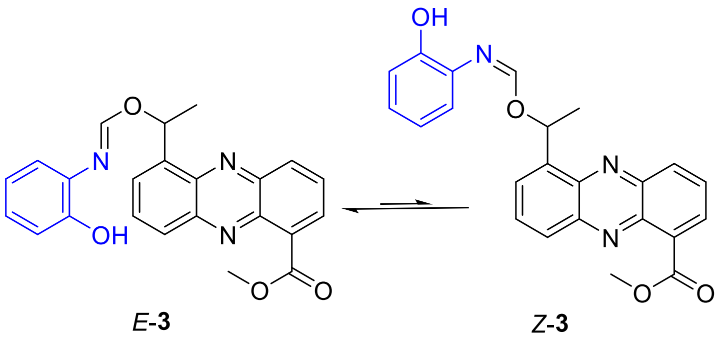

Figure S14. ^{1}H-NMR spectrum (600 MHz) of **3** in pyridine-d_5

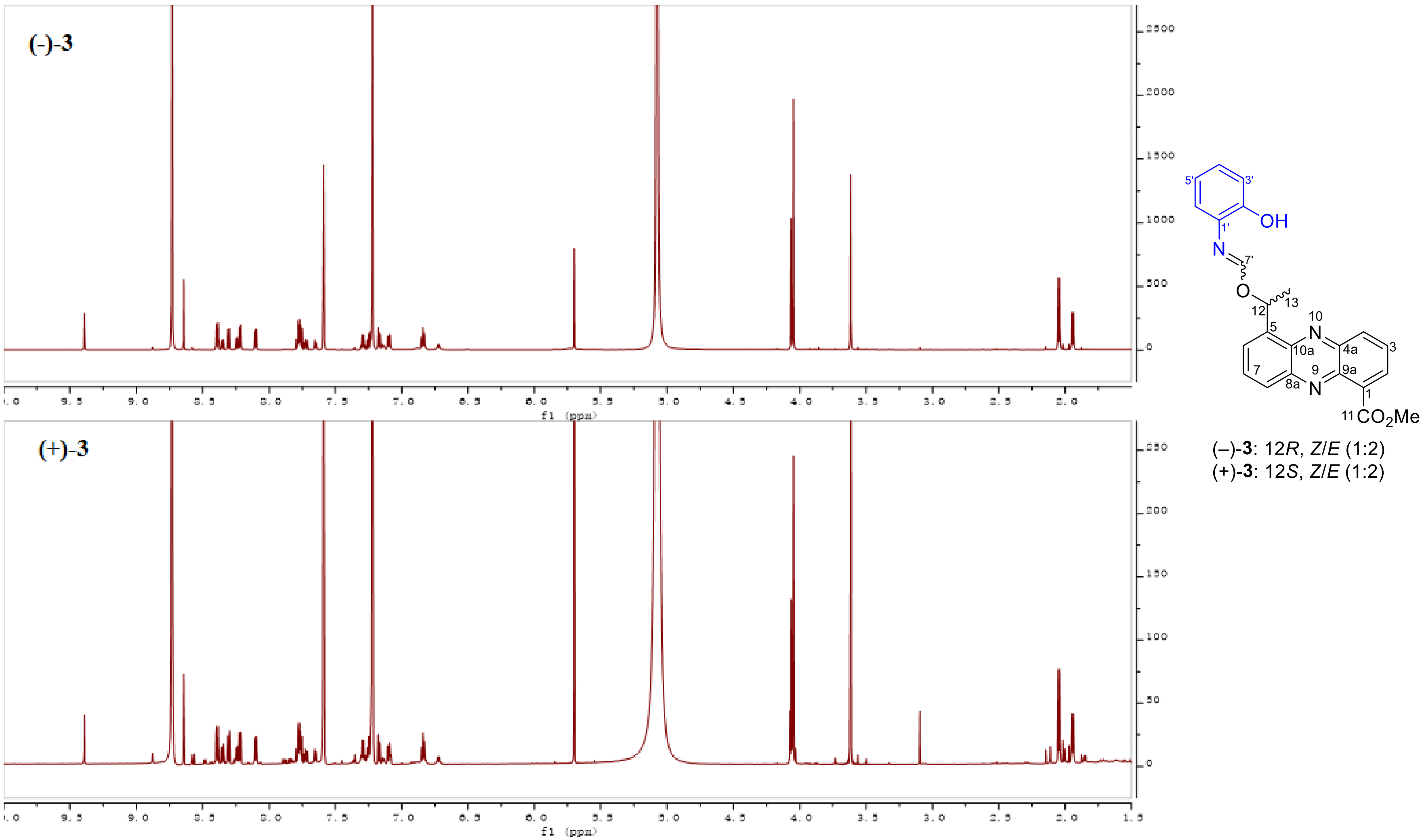

Figure S15. DEPTQ spectra (150 MHz) of **3** in pyridine-d_5

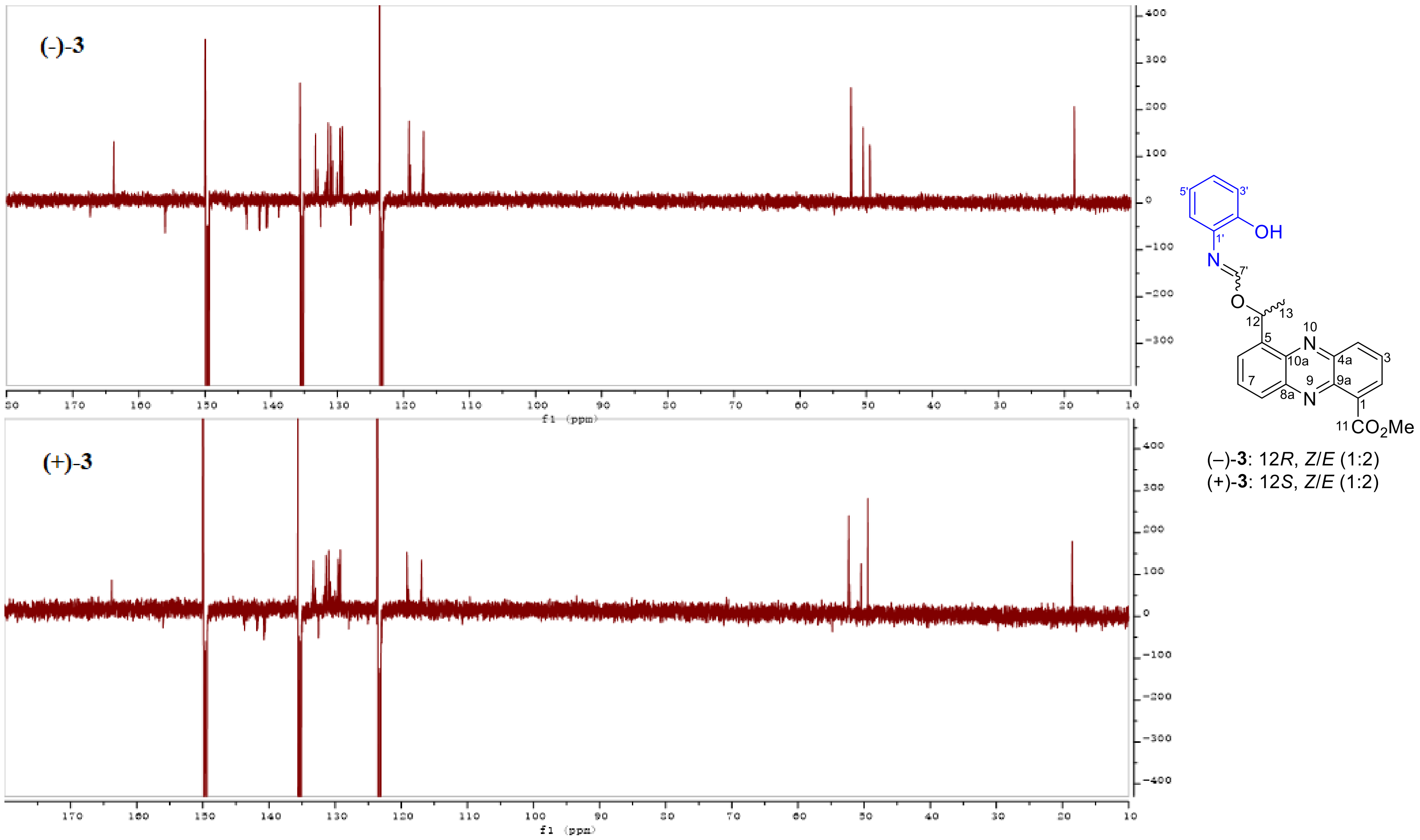

Figure S16. ^{1}H-NMR spectrum (600 MHz) of **3** in pyridine-d_5

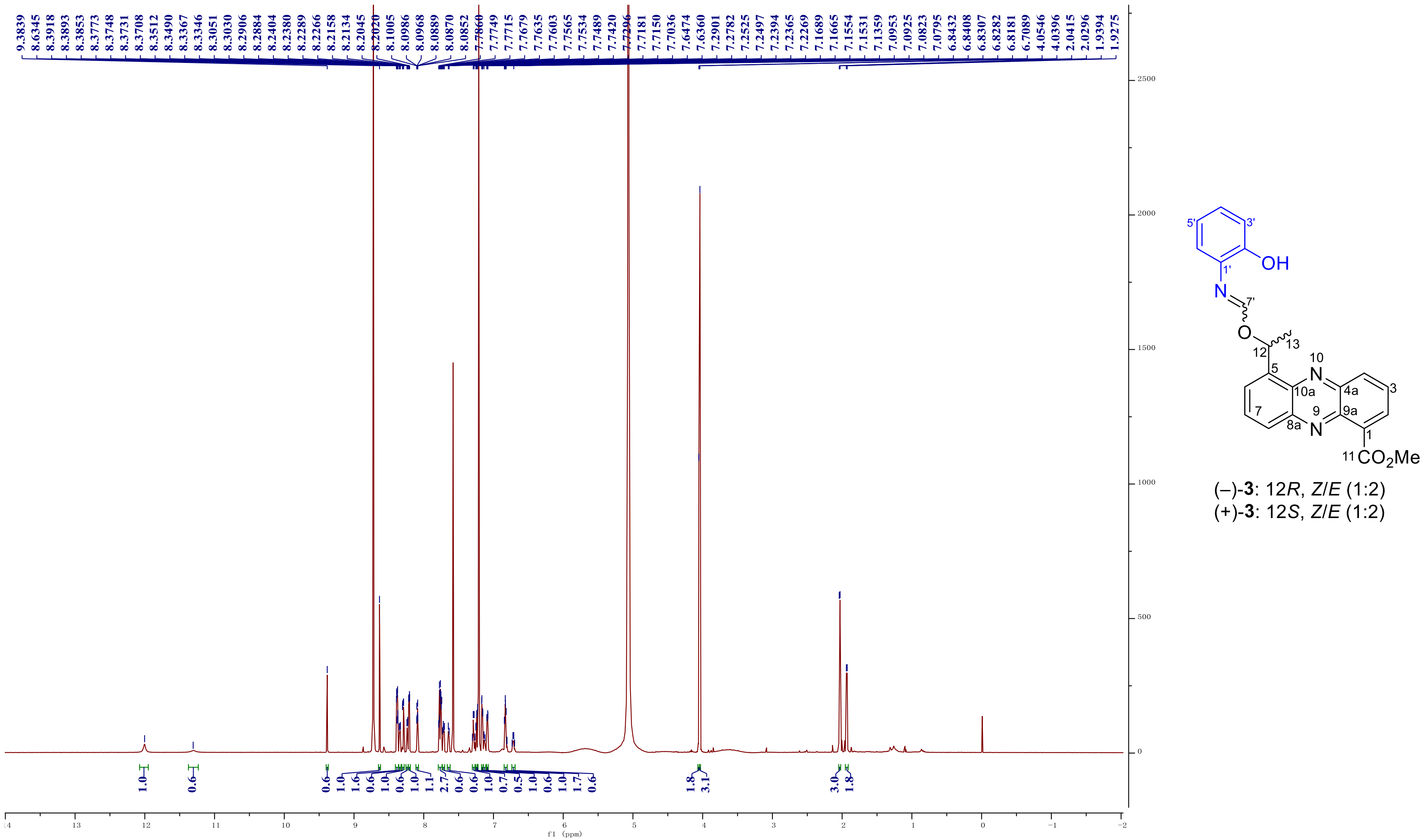

Figure S17. Amplified ^{1}H-NMR spectrum (600 MHz) of **3** in pyridine-d_5

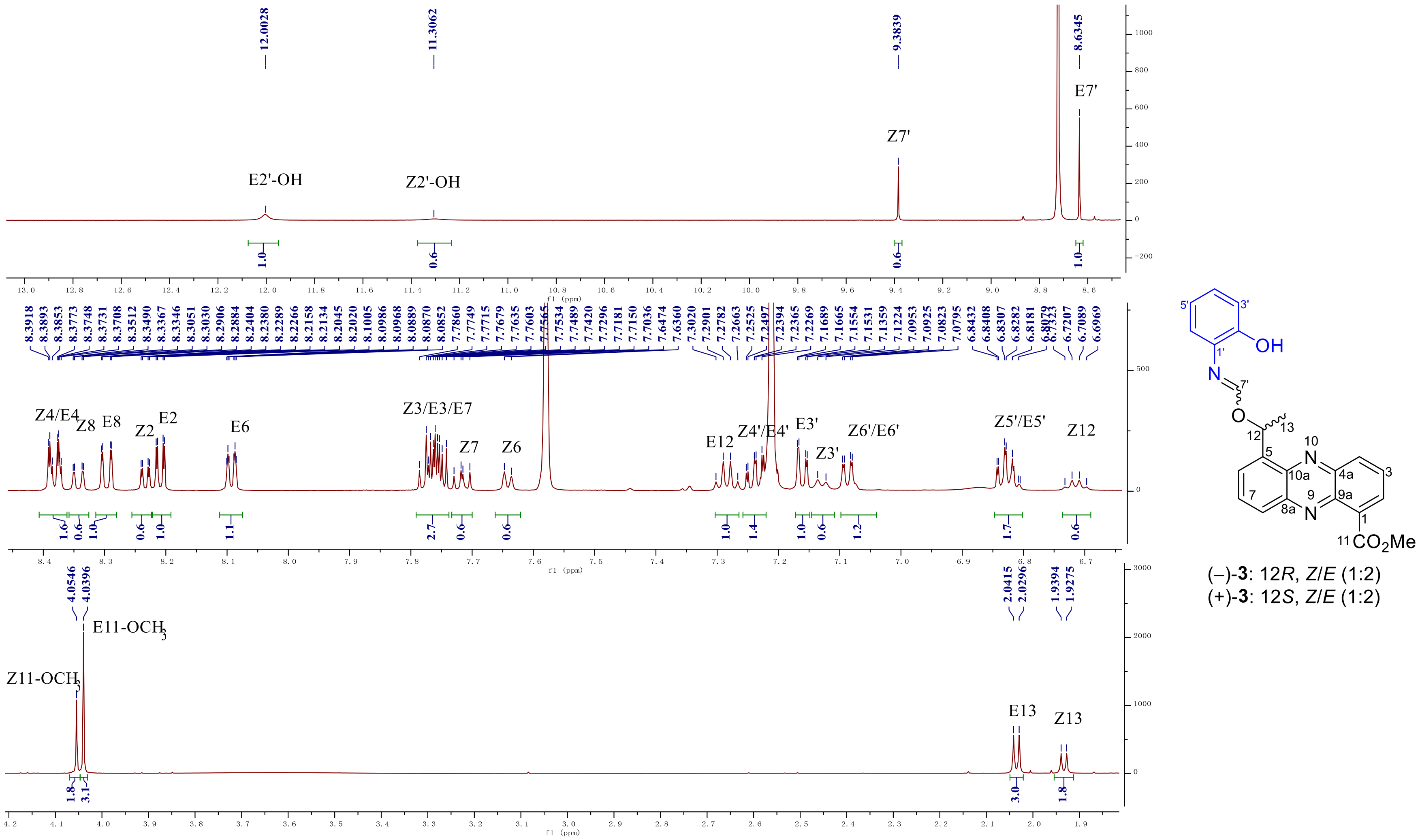

(–)-**3**: 12*R*, *Z*/*E* (1:2)
(+)-**3**: 12*S*, *Z*/*E* (1:2)

Figure S18. DEPTQ spectrum (150 MHz) of **3** in pyridine-d_5

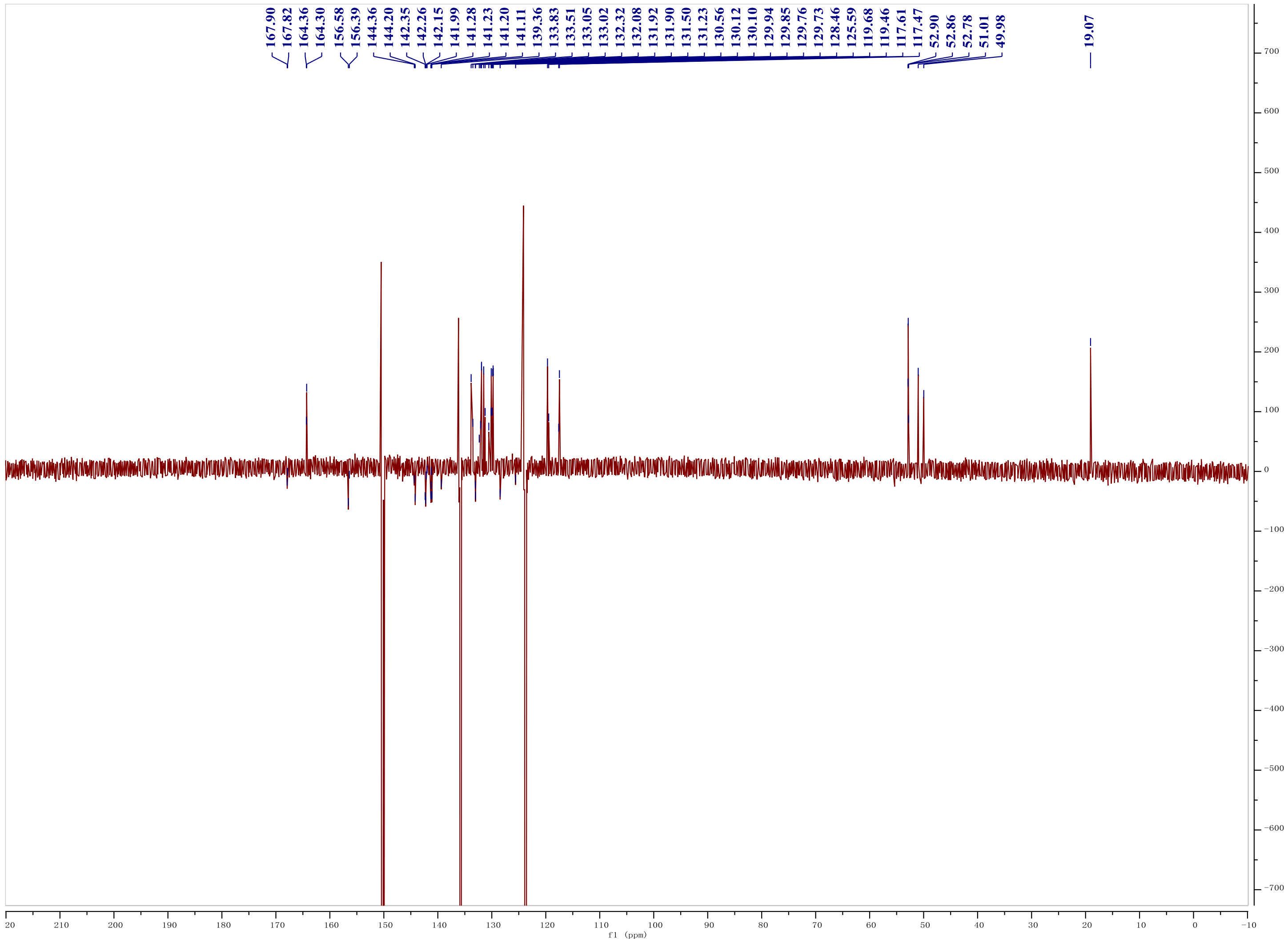

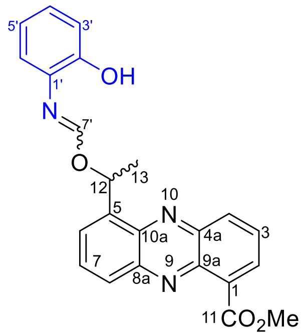

(–)-**3**: 12*R*, *Z*/*E* (1:2)
(+)-**3**: 12*S*, *Z*/*E* (1:2)

Figure S19. Amplified DEPTQ spectrum (150 MHz) of **3** in pyridine-d_5

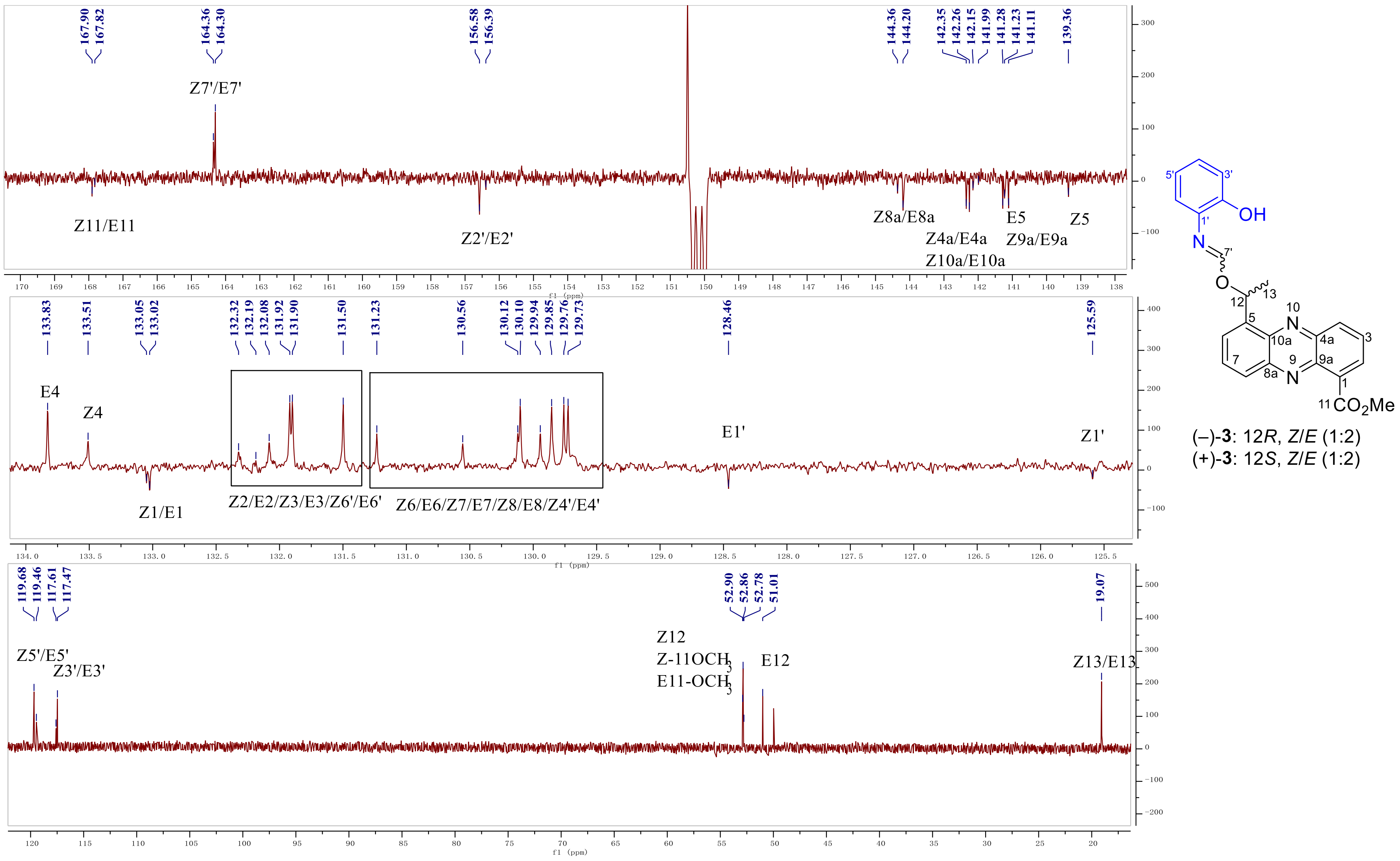

Figure S20. ^{1}H-^{1}H COSY spectrum (600 MHz) of **3** in pyridine-d_5

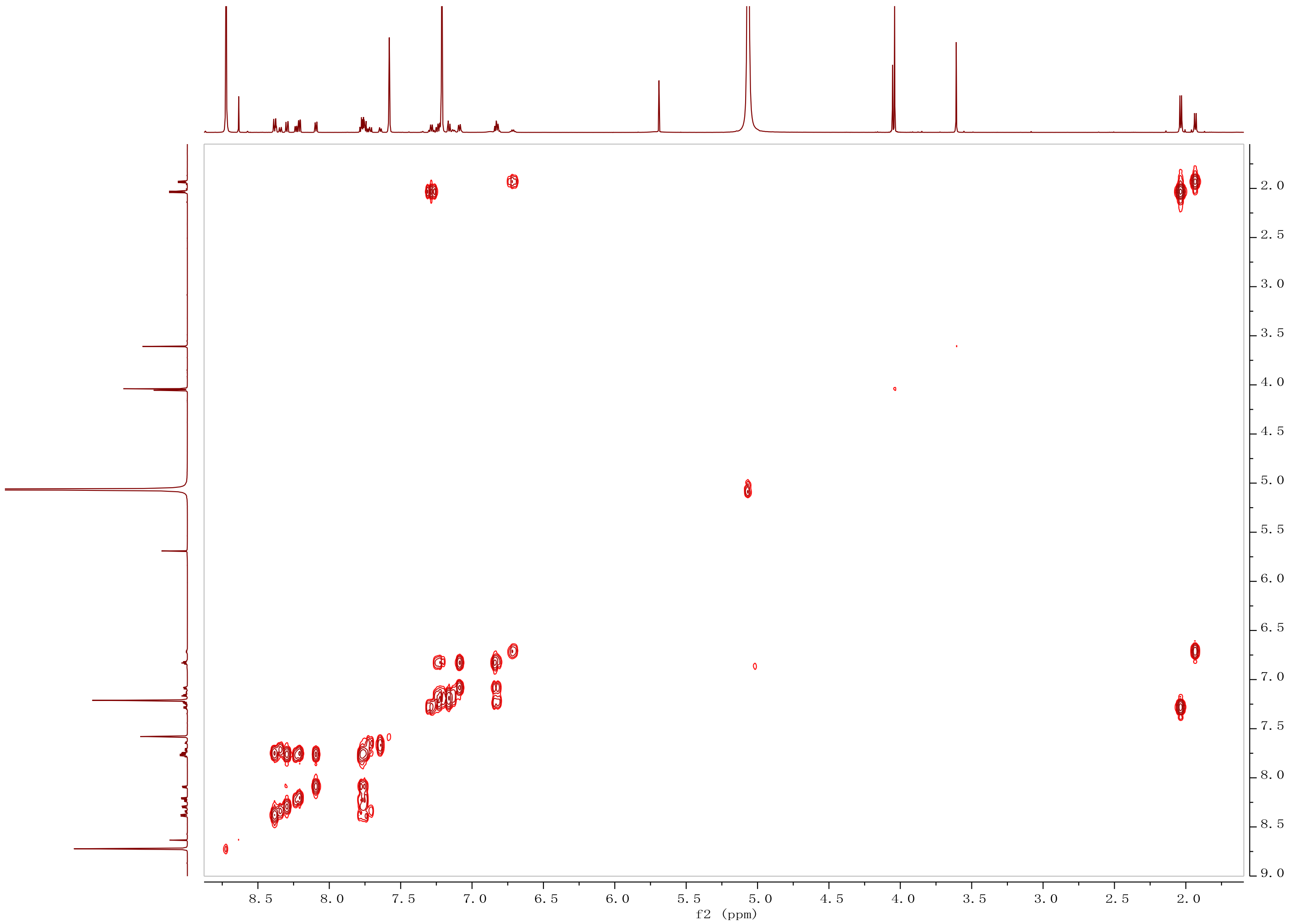

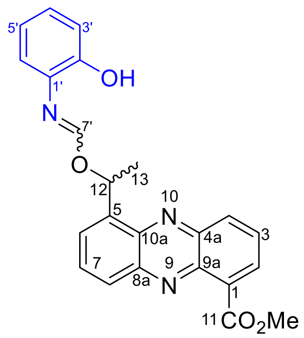

(–)-**3**: 12*R*, *Z*/*E* (1:2)
(+)-**3**: 12*S*, *Z*/*E* (1:2)

Figure S21. HSQC spectrum (600 MHz, 150 MHz) of **3** in pyridine-*d_5*

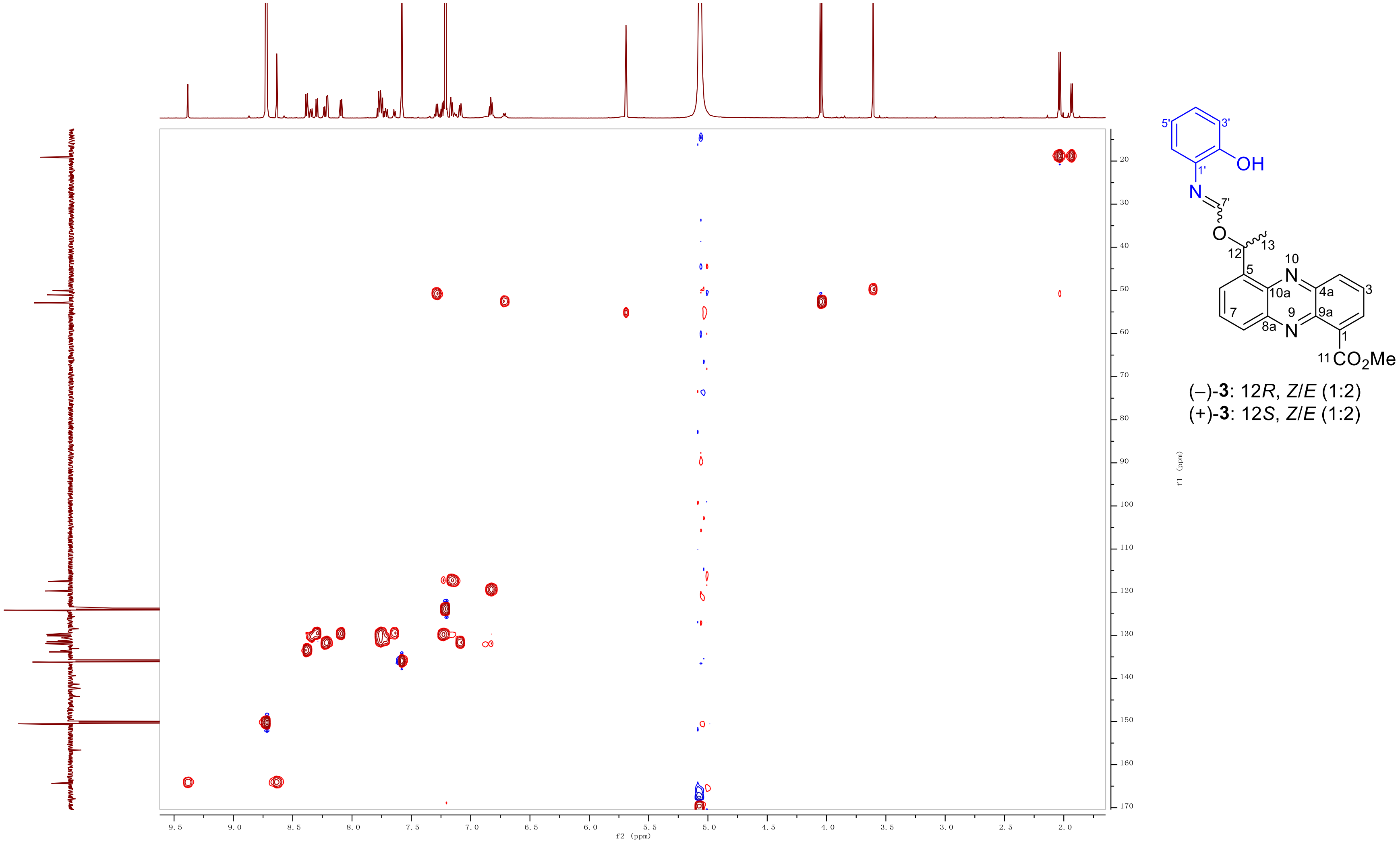

Figure S22. HMBC spectrum (600 MHz, 150 MHz) of **3** in pyridine-d_5

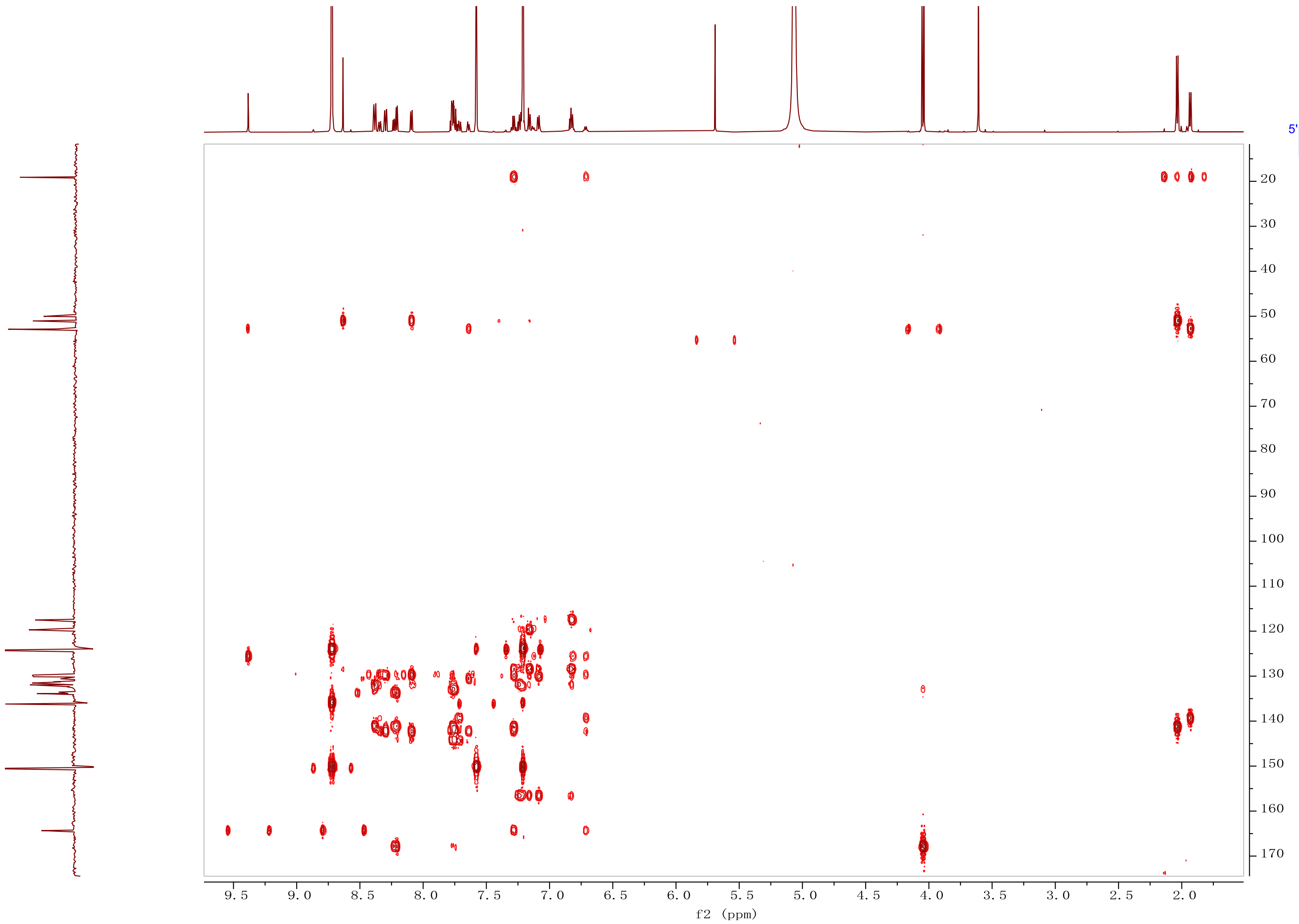

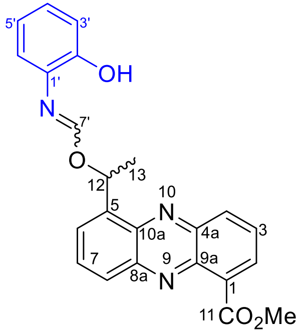

(–)-**3**: 12*R*, *Z*/*E* (1:2)
(+)-**3**: 12*S*, *Z*/*E* (1:2)

Figure S23. NOEdiff spectrum (500 MHz) of **3** in pyridine-d_5

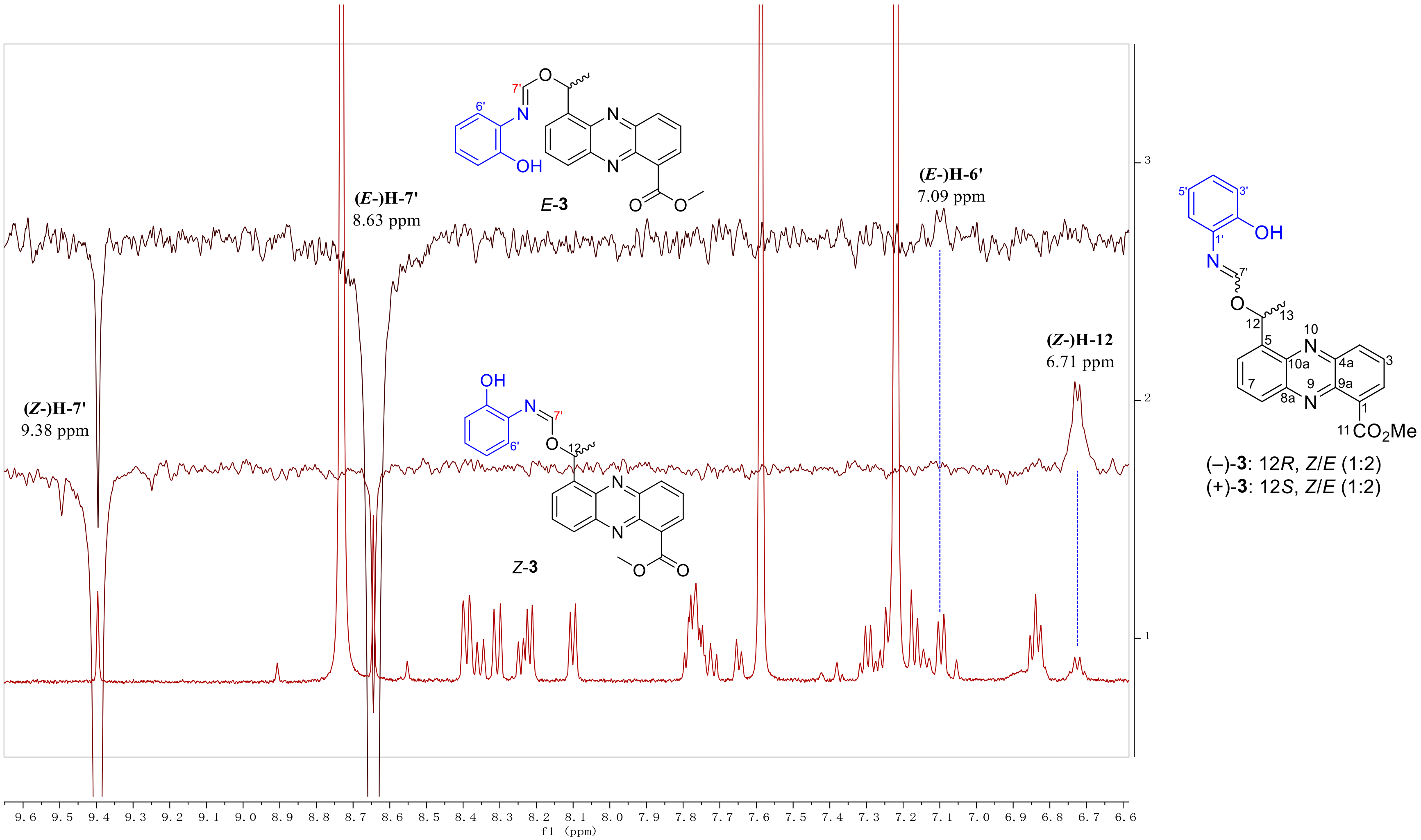

Figure S24. HRESIMS spectrum of **4**

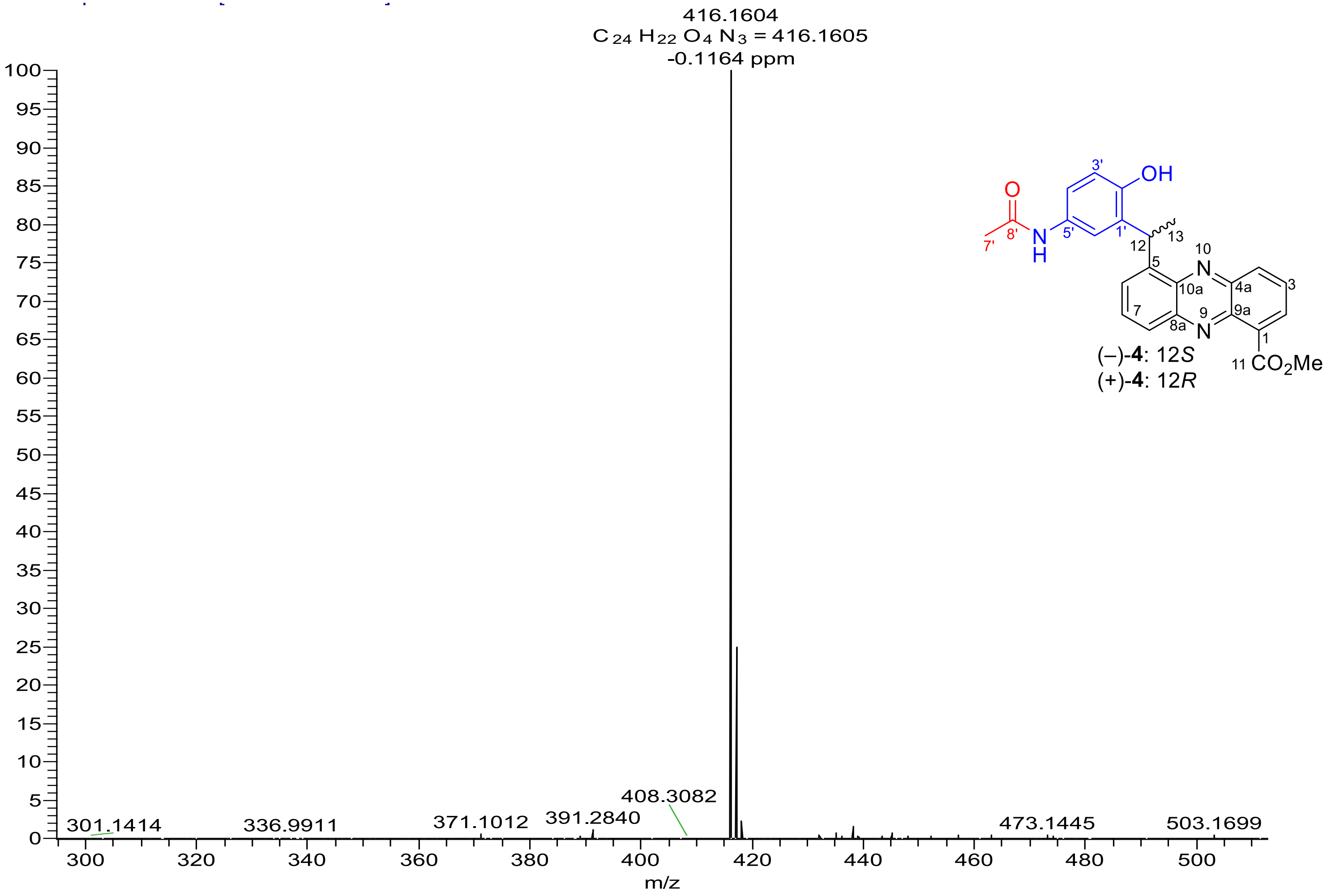

Figure S25. ^{1}H-NMR spectrum (600 MHz) of **4** in DMSO-d_6

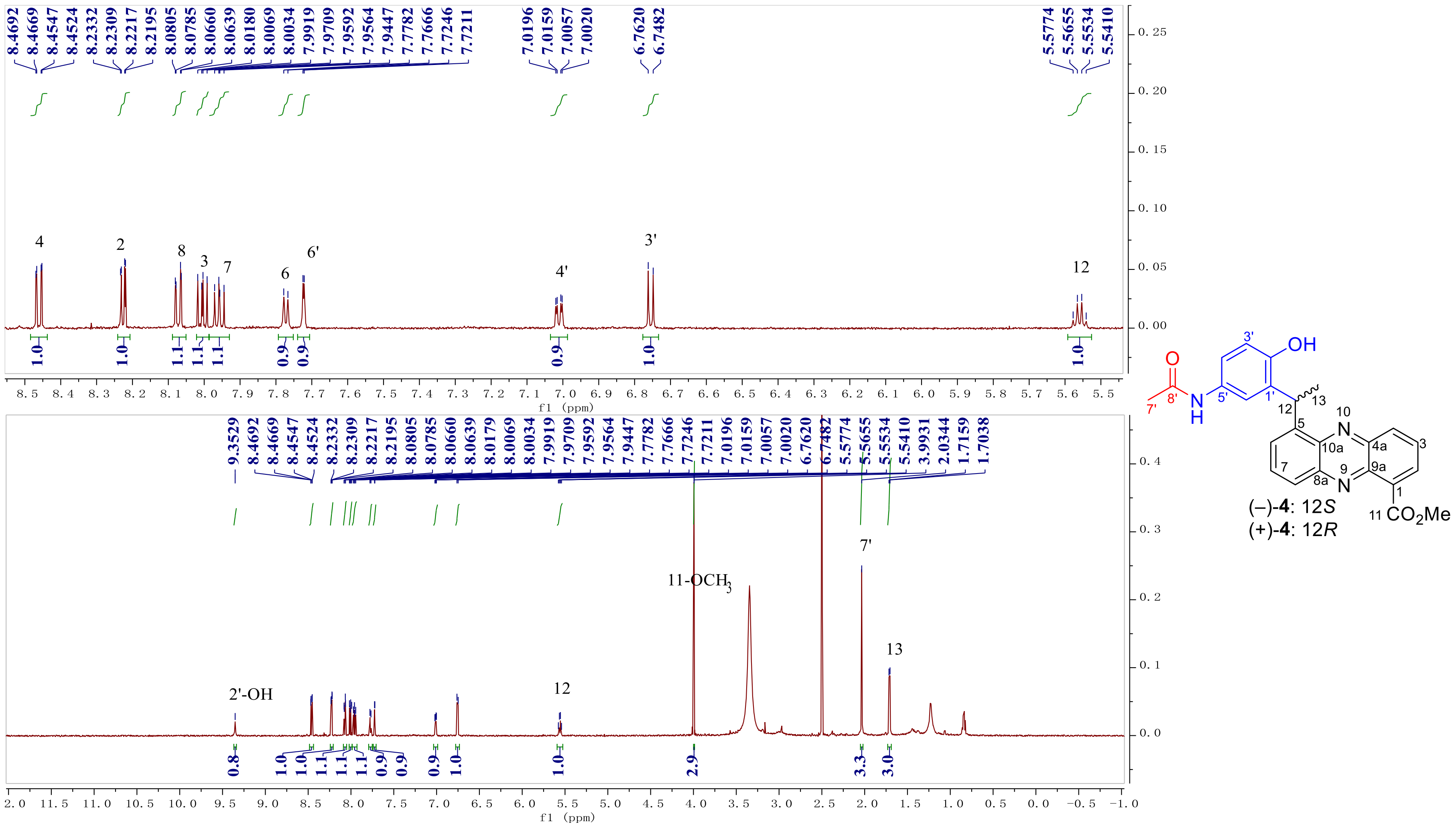

Figure S26. ^{13}C-NMR spectrum (150 MHz) of **4** in DMSO-d_6

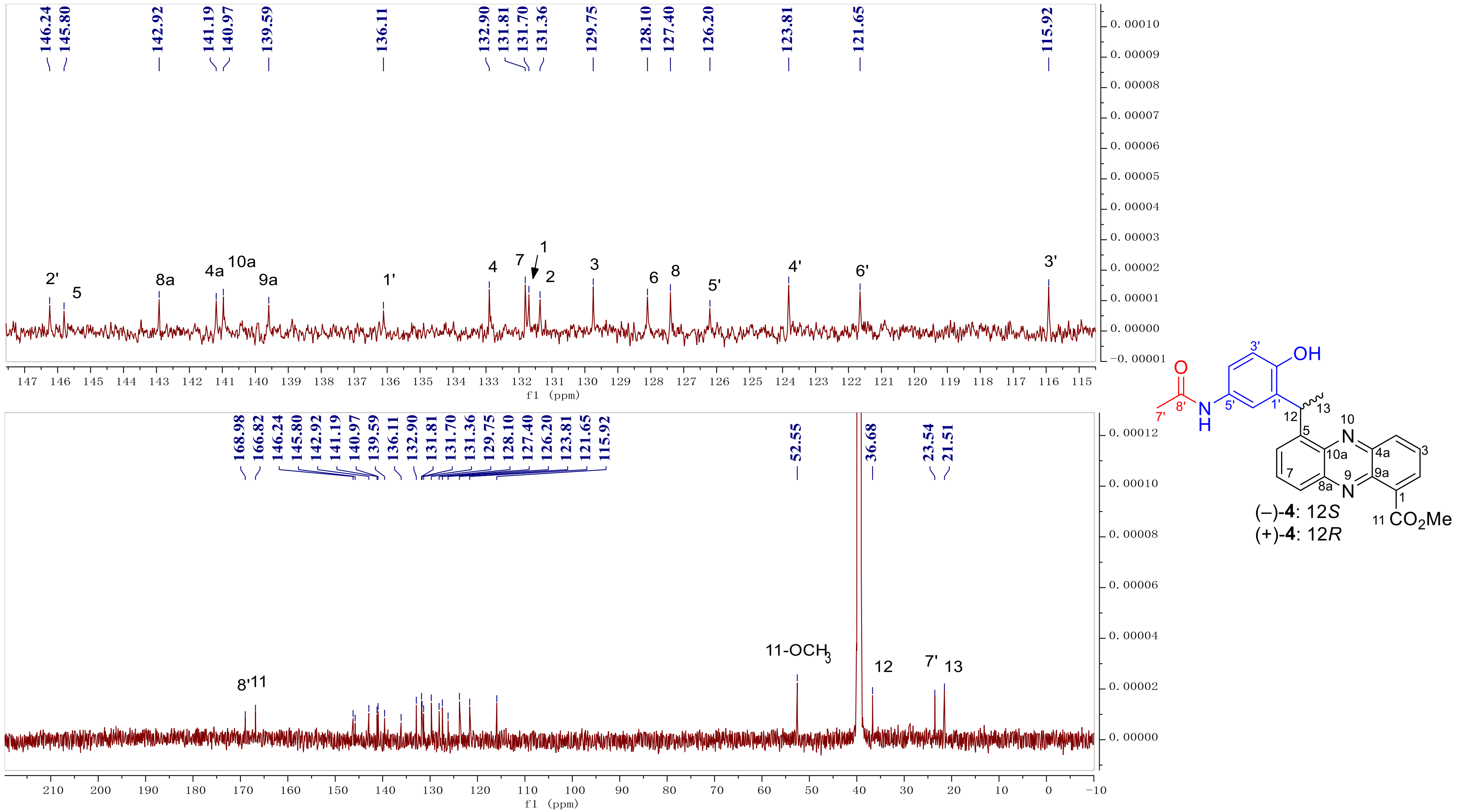

Figure S27. ^{1}H-^{1}H COSY spectrum (600 MHz) of **4** in DMSO-d_6

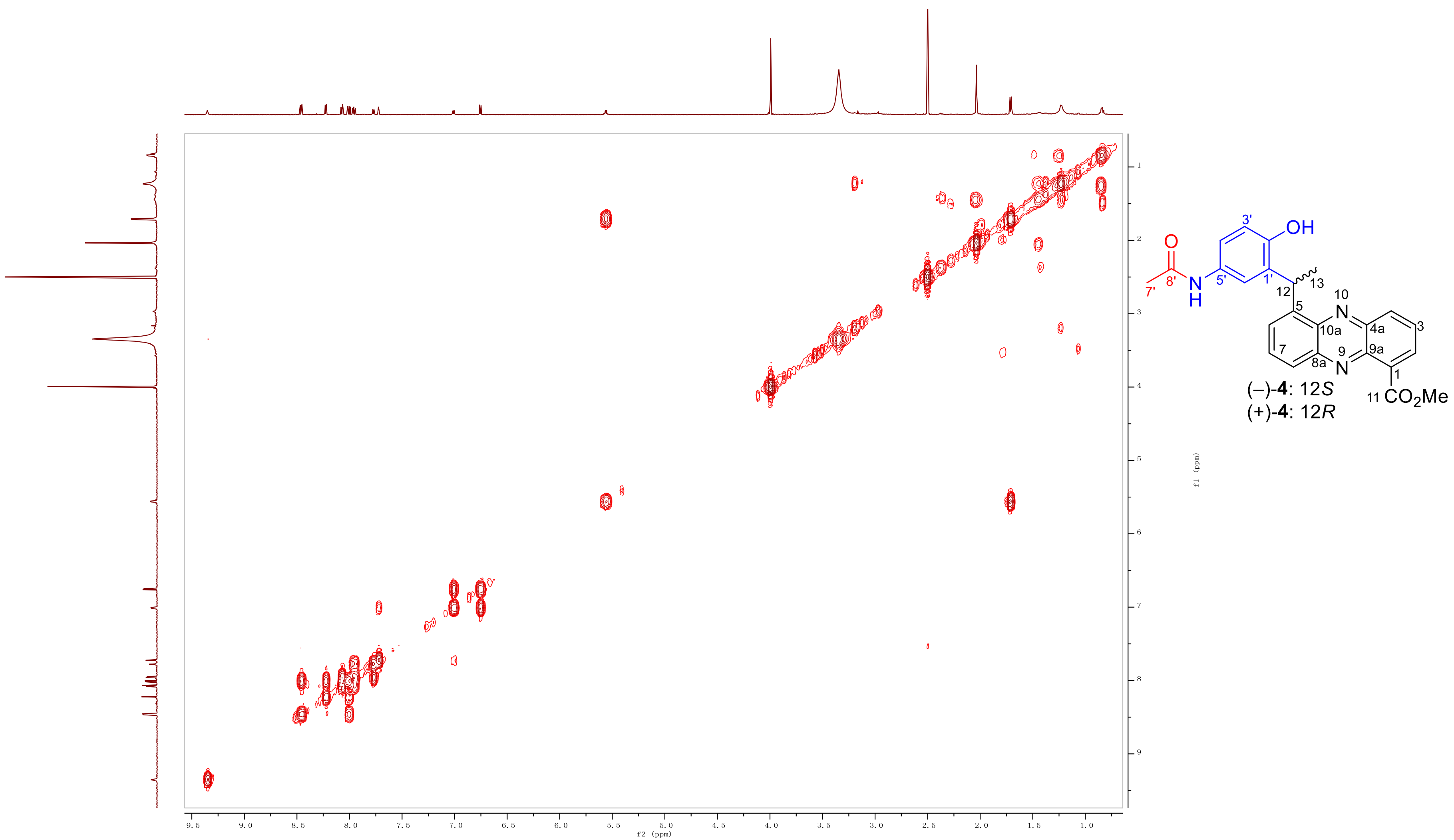

Figure S28. HSQC spectrum (600 MHz, 150 MHz) of **4** in DMSO-d_6

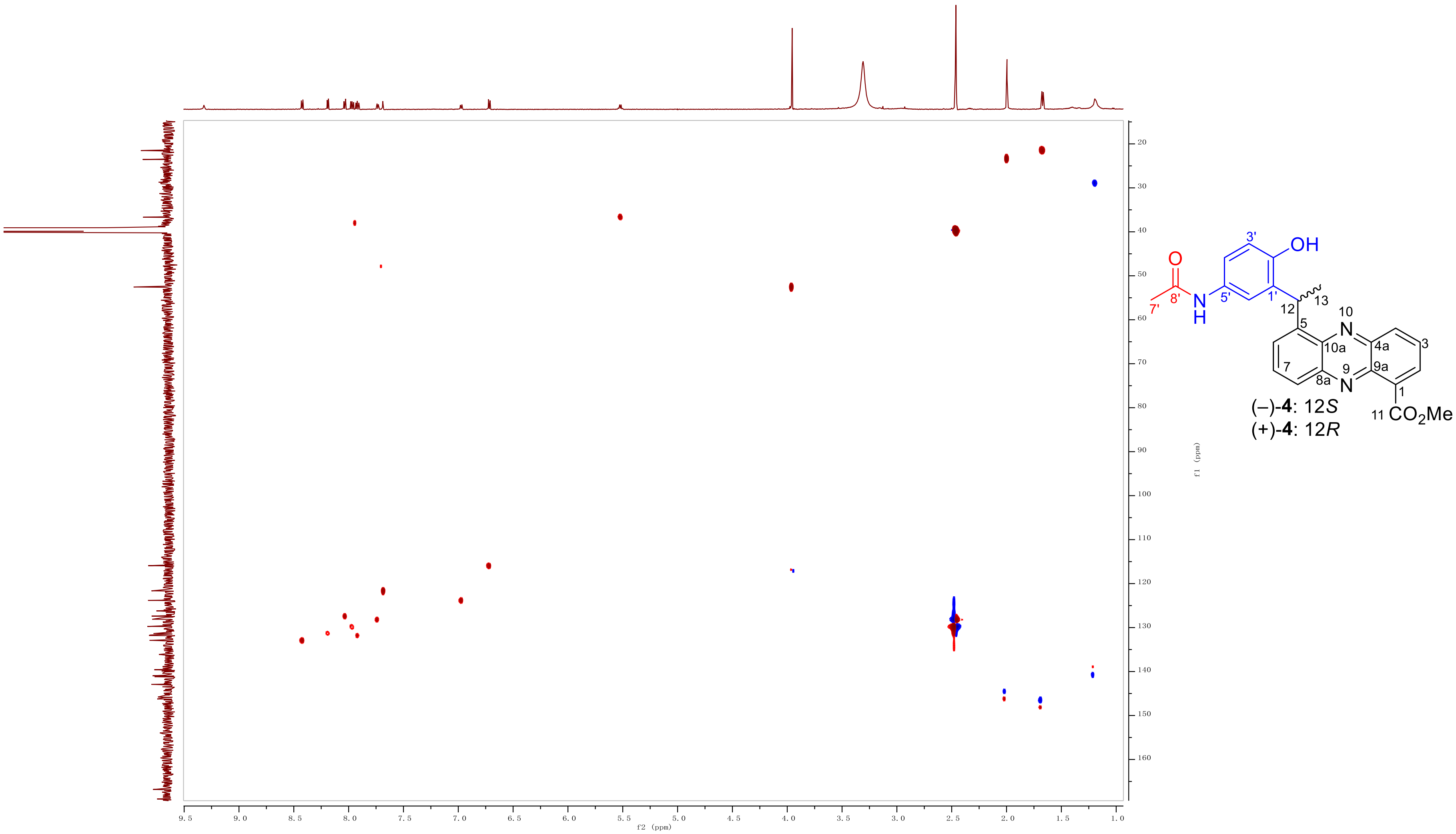

Figure S29. HMBC spectrum (600 MHz, 150 MHz) of **4** in DMSO-d_6

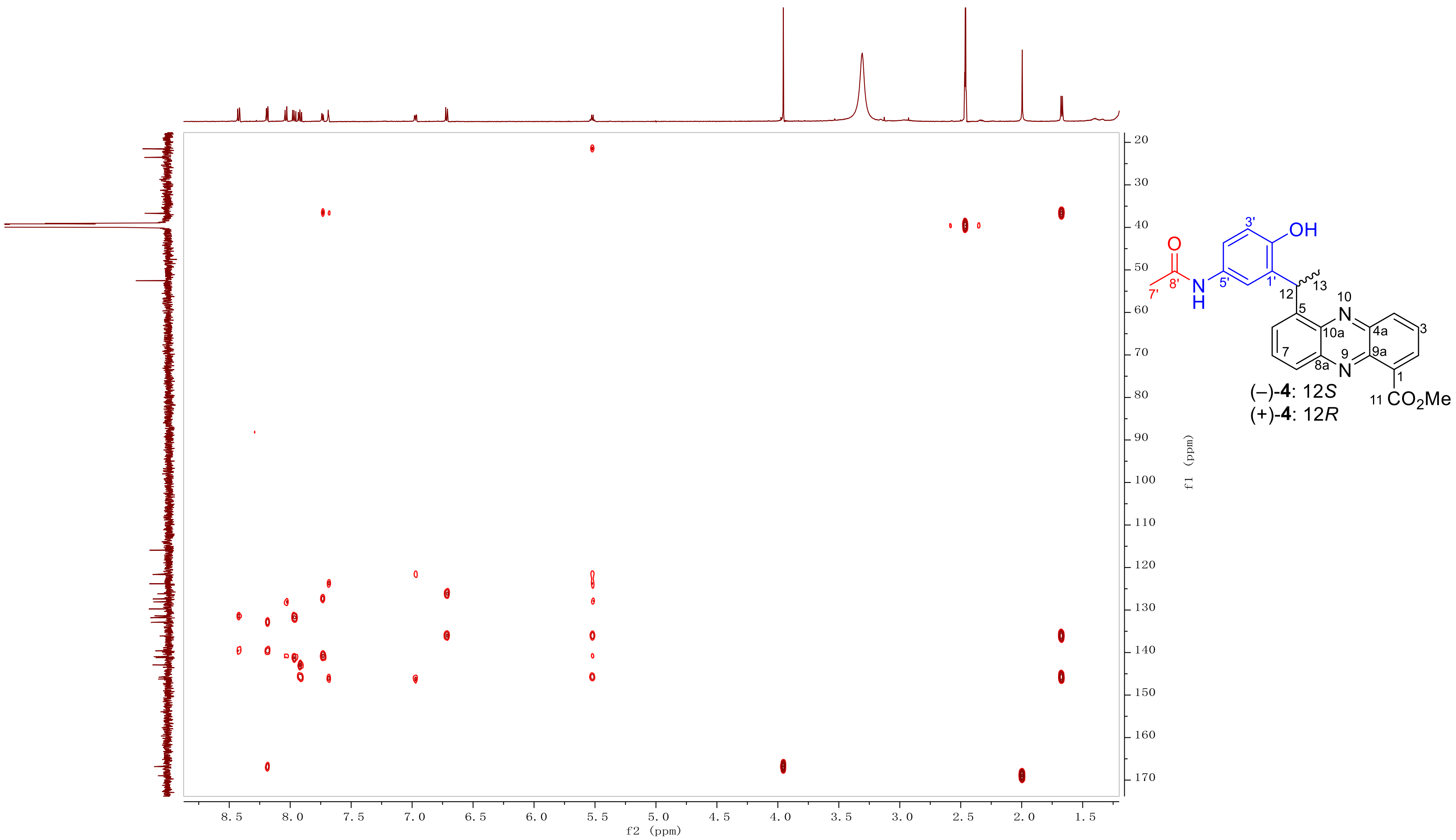

Figure S30. HRESIMS spectrum of **5**

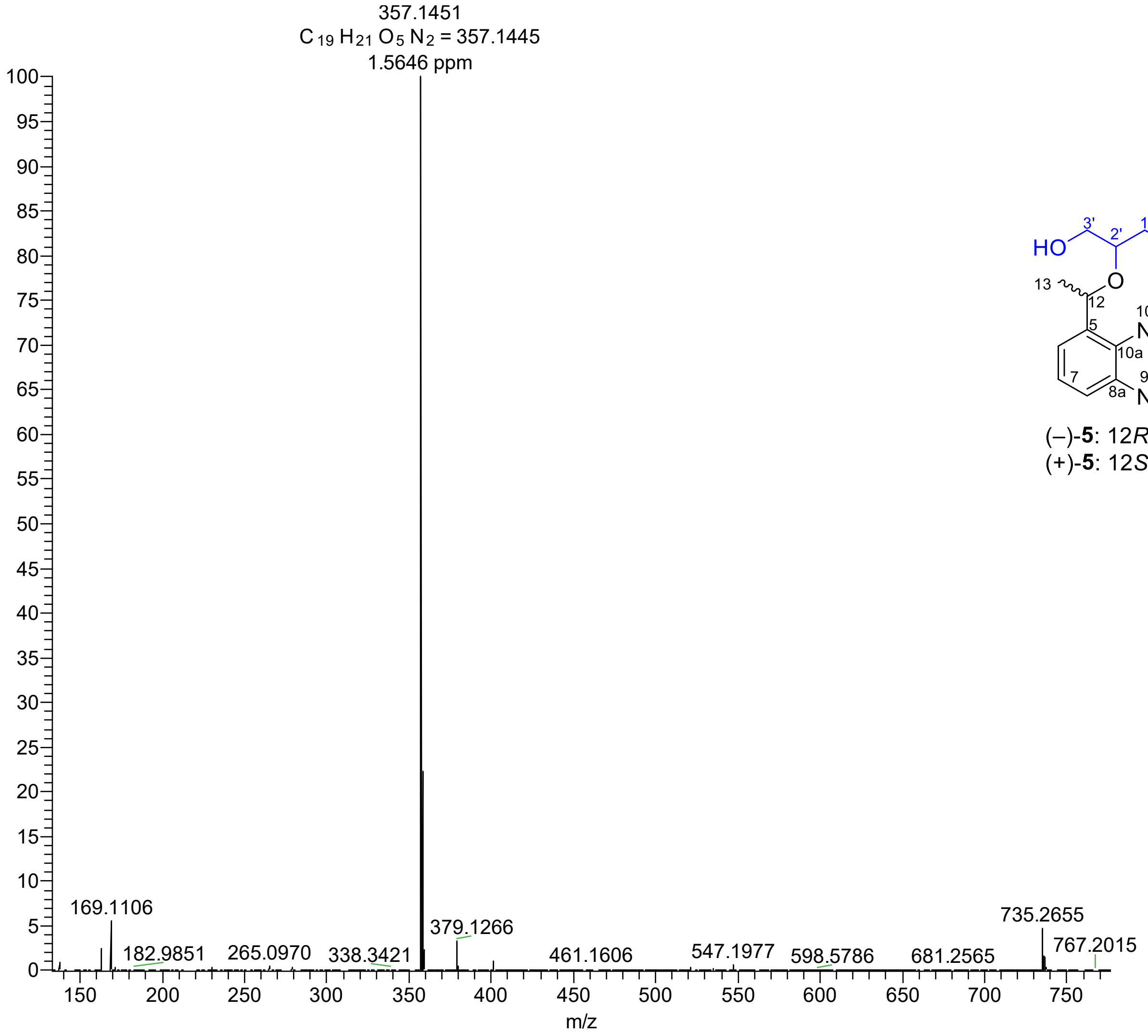

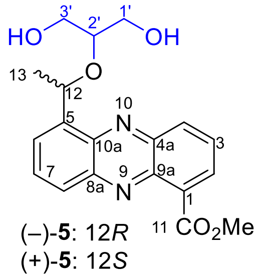

Figure S31. ^{1}H-NMR spectrum (500 MHz) of **5** in DMSO-d_6

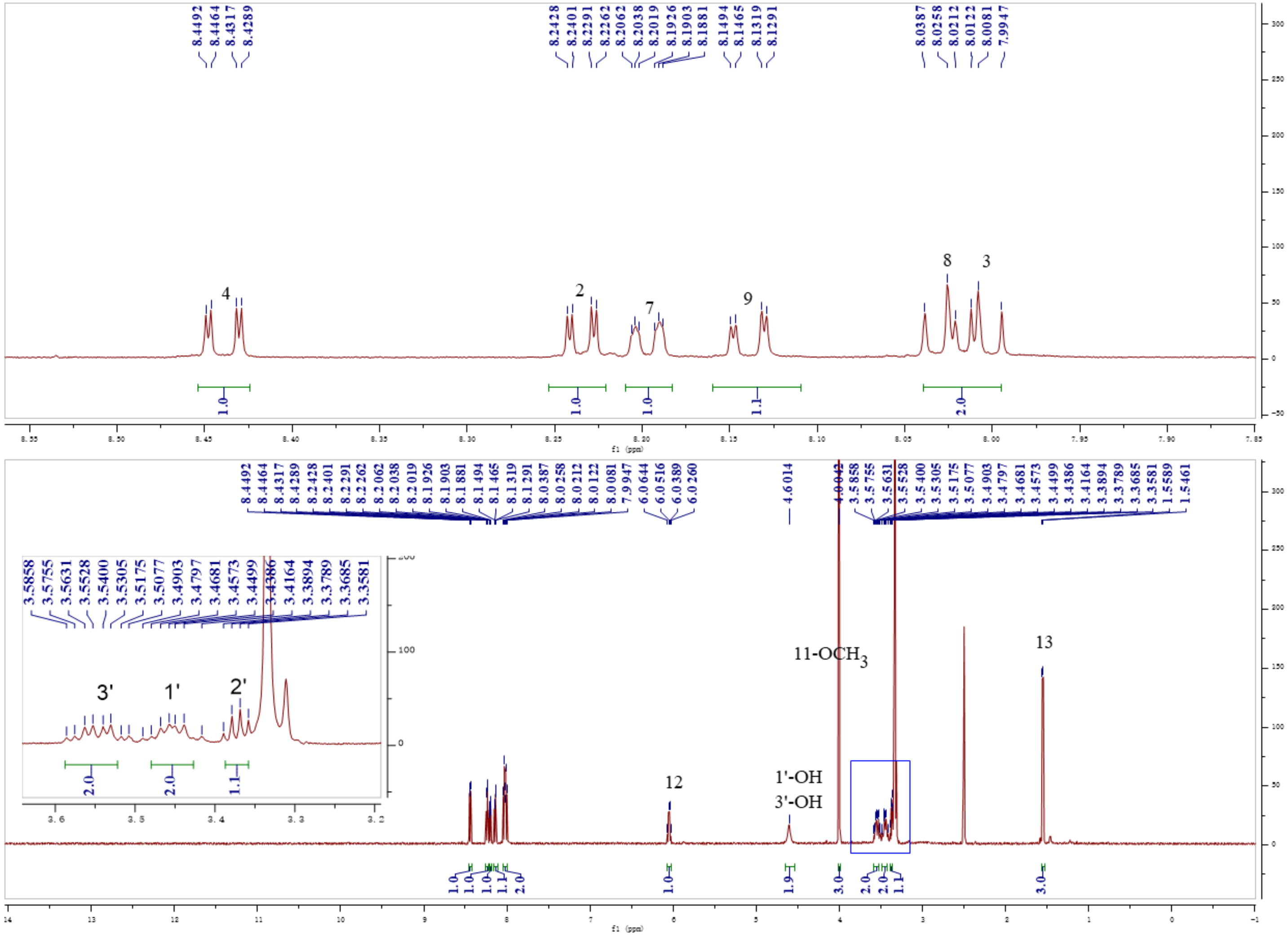

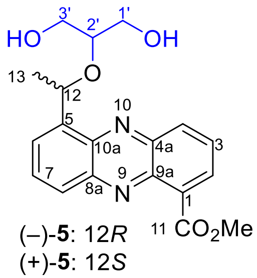

Figure S32. ^{13}C-NMR spectrum (125 MHz) of **5** in DMSO-d_6

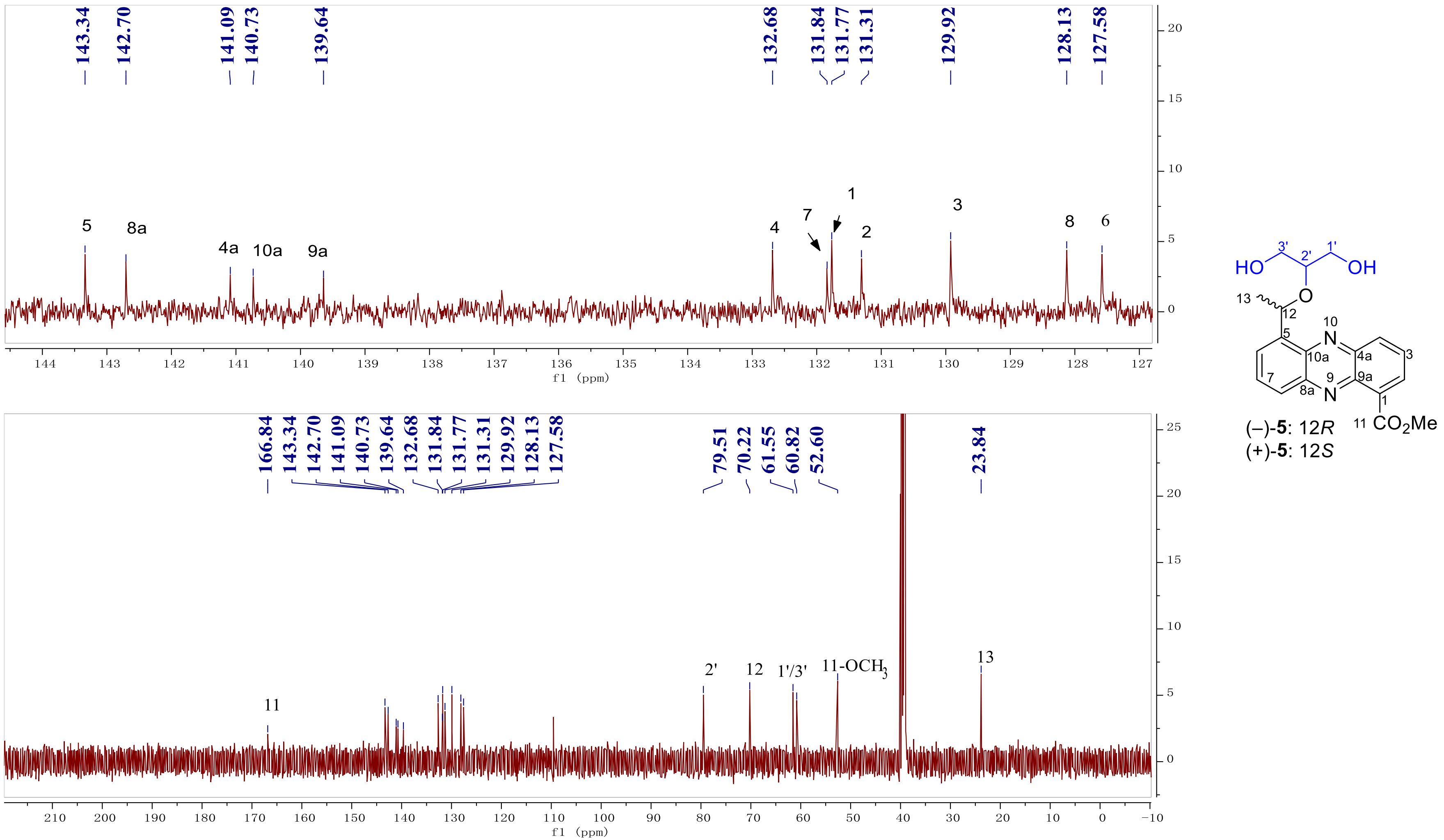

Figure S33. ^1H-^1H COSY spectrum (500 MHz) of **5** in DMSO-d_6

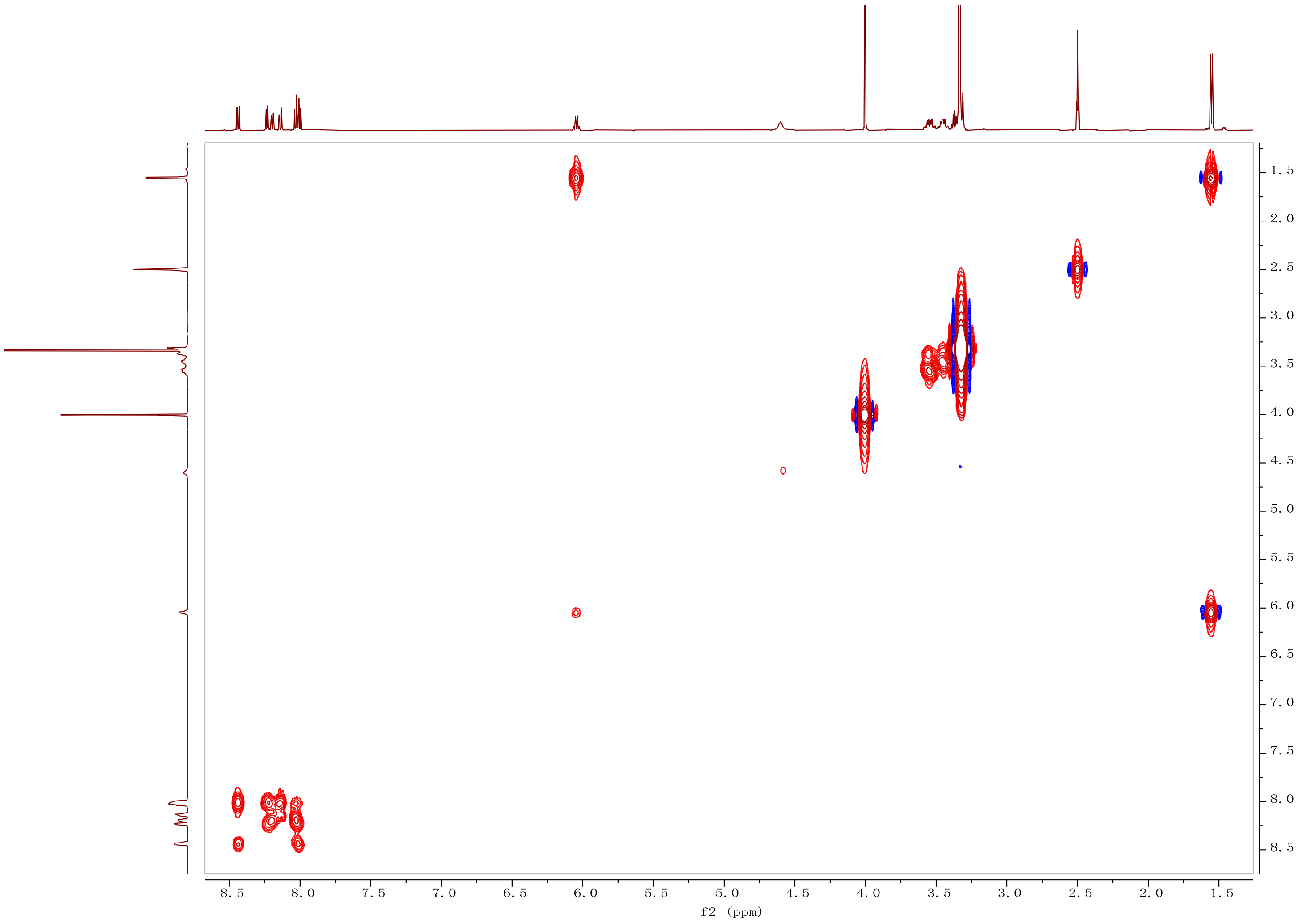

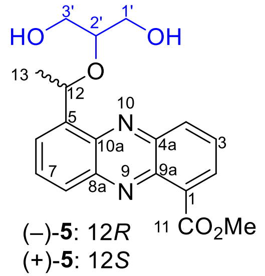

Figure S34. HSQC spectrum (500 MHz, 125 MHz) of **5** in DMSO-d_6

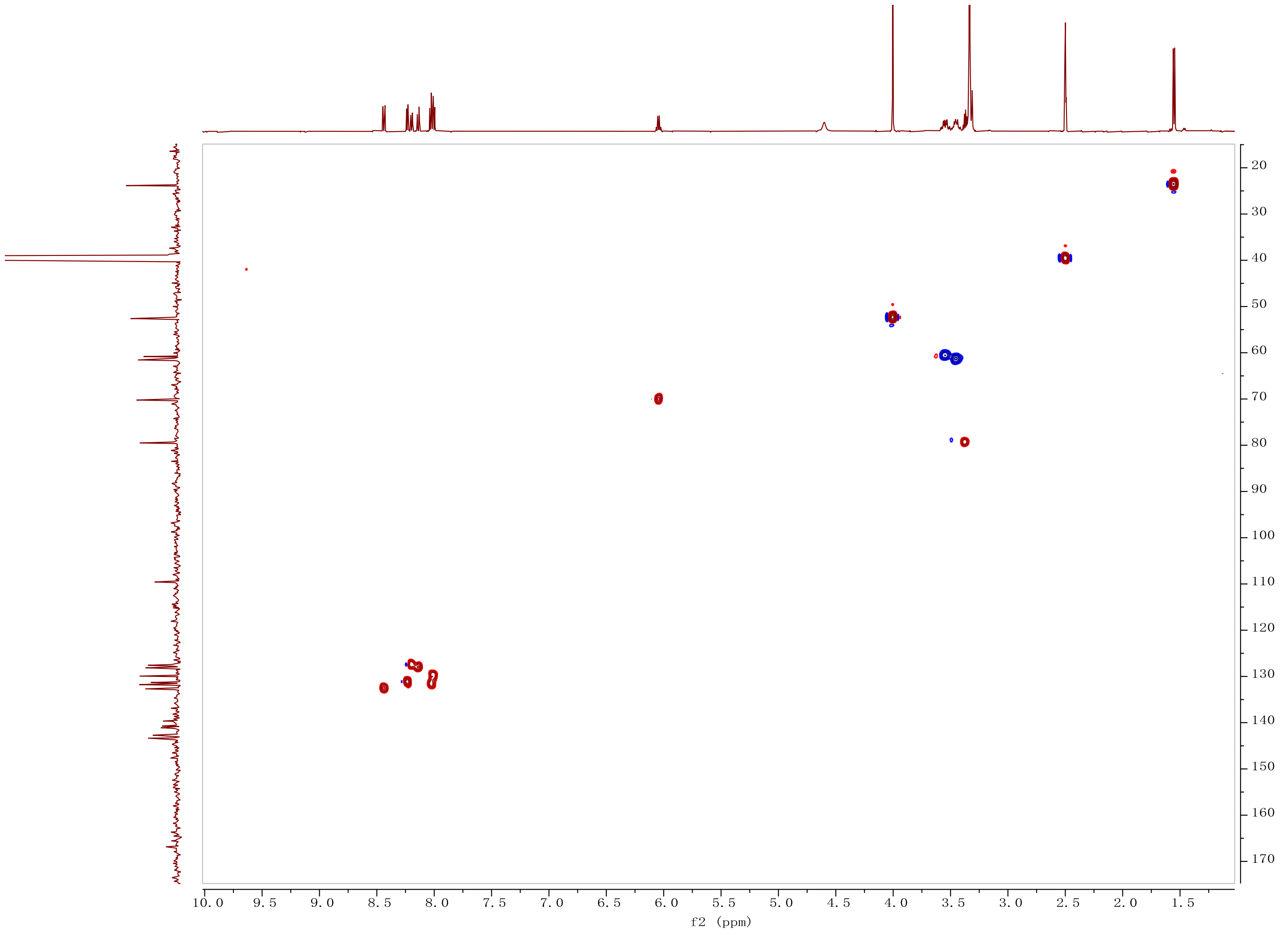

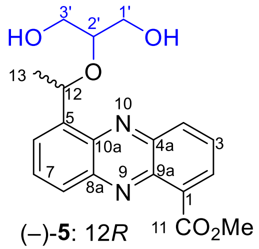

(−)-**5**: 12*R*
(+)-**5**: 12*S*

Figure S35. HMBC spectrum (500 MHz, 125 MHz) of **5** in DMSO-d_6

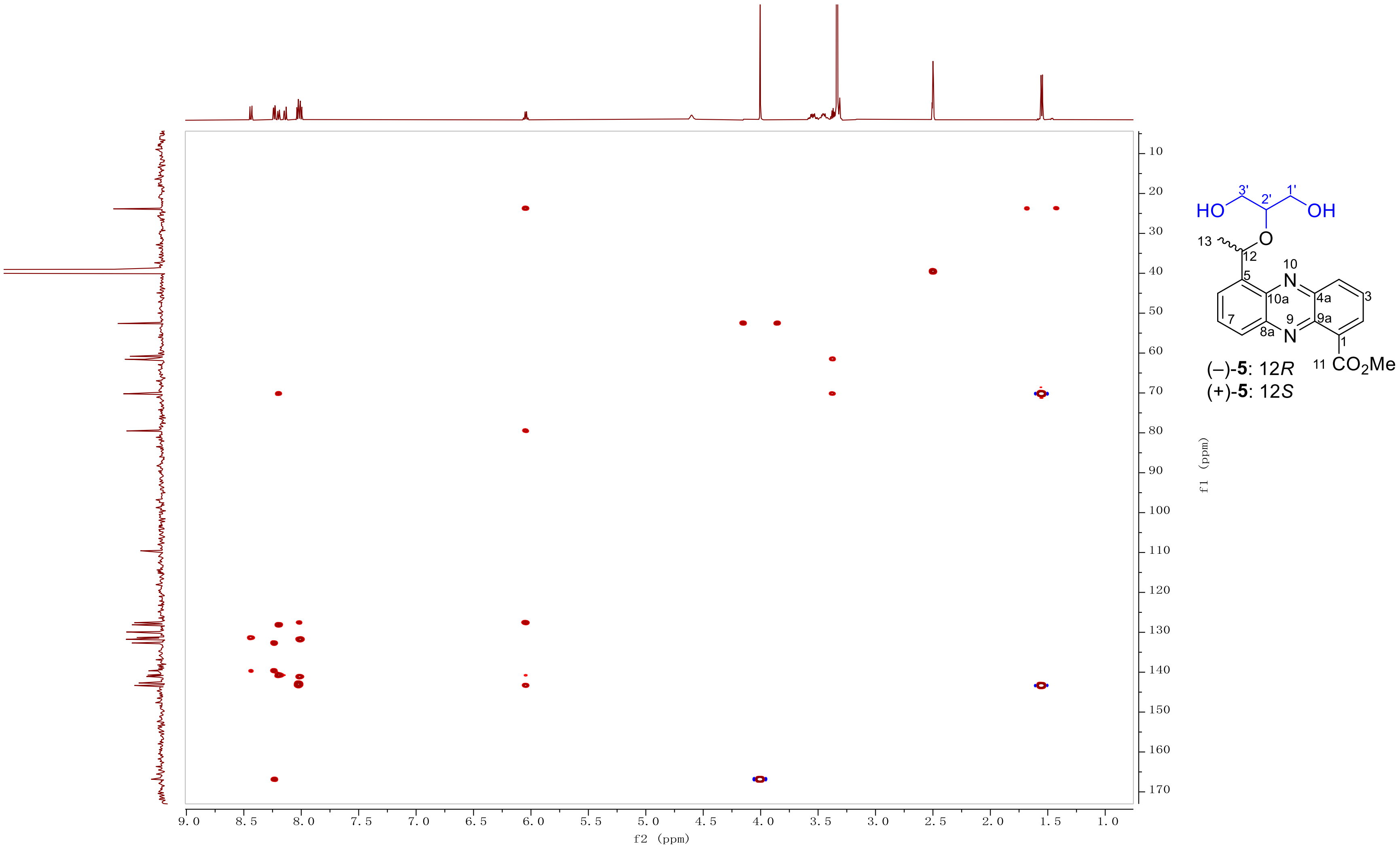

Figure S36. ^{1}H-NMR spectrum (600 MHz) of synthetic **1** in DMSO-d_6

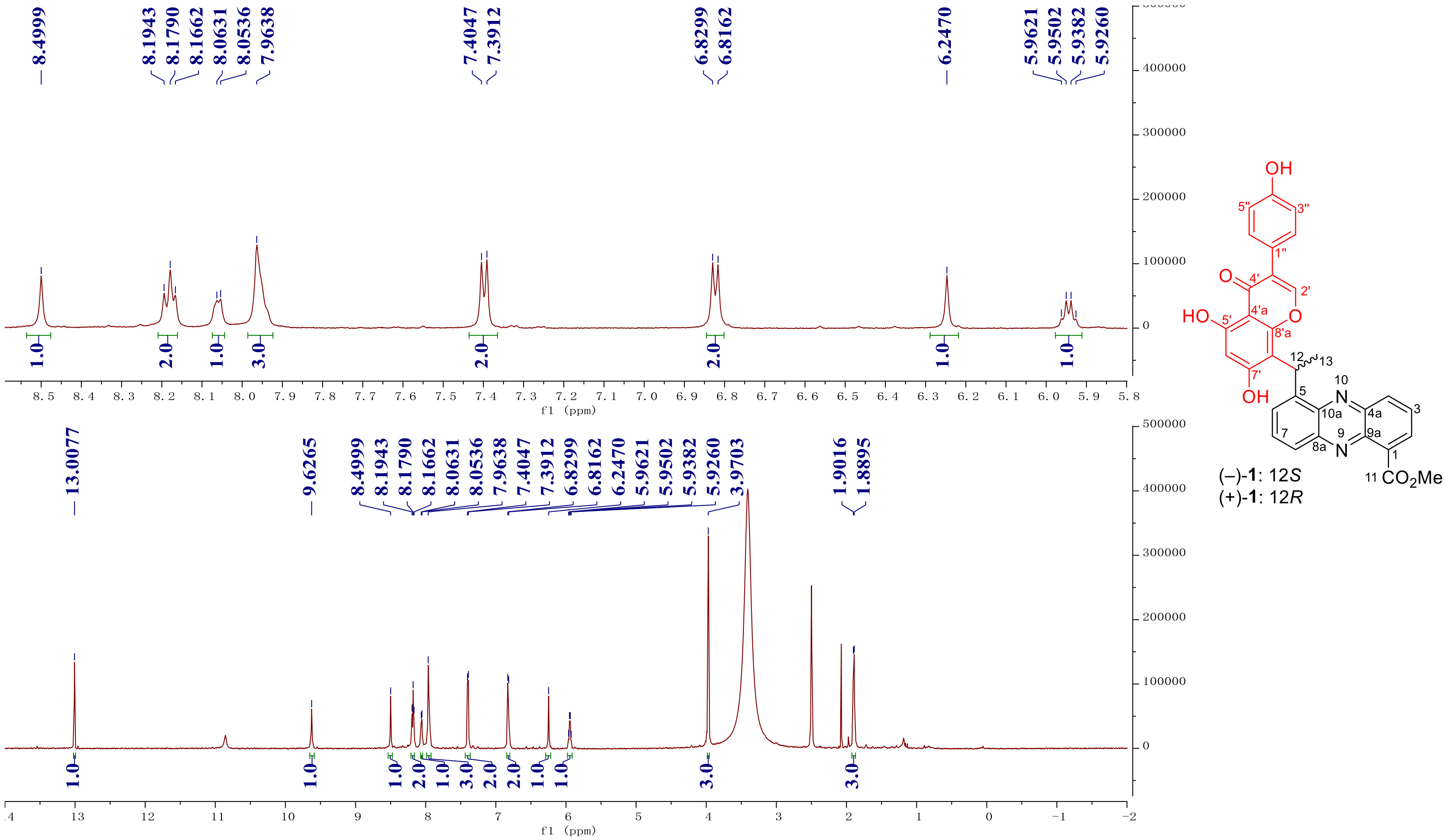

Figure S37. ^{1}H-NMR spectrum (600 MHz) of synthetic **2** in DMSO-d_6

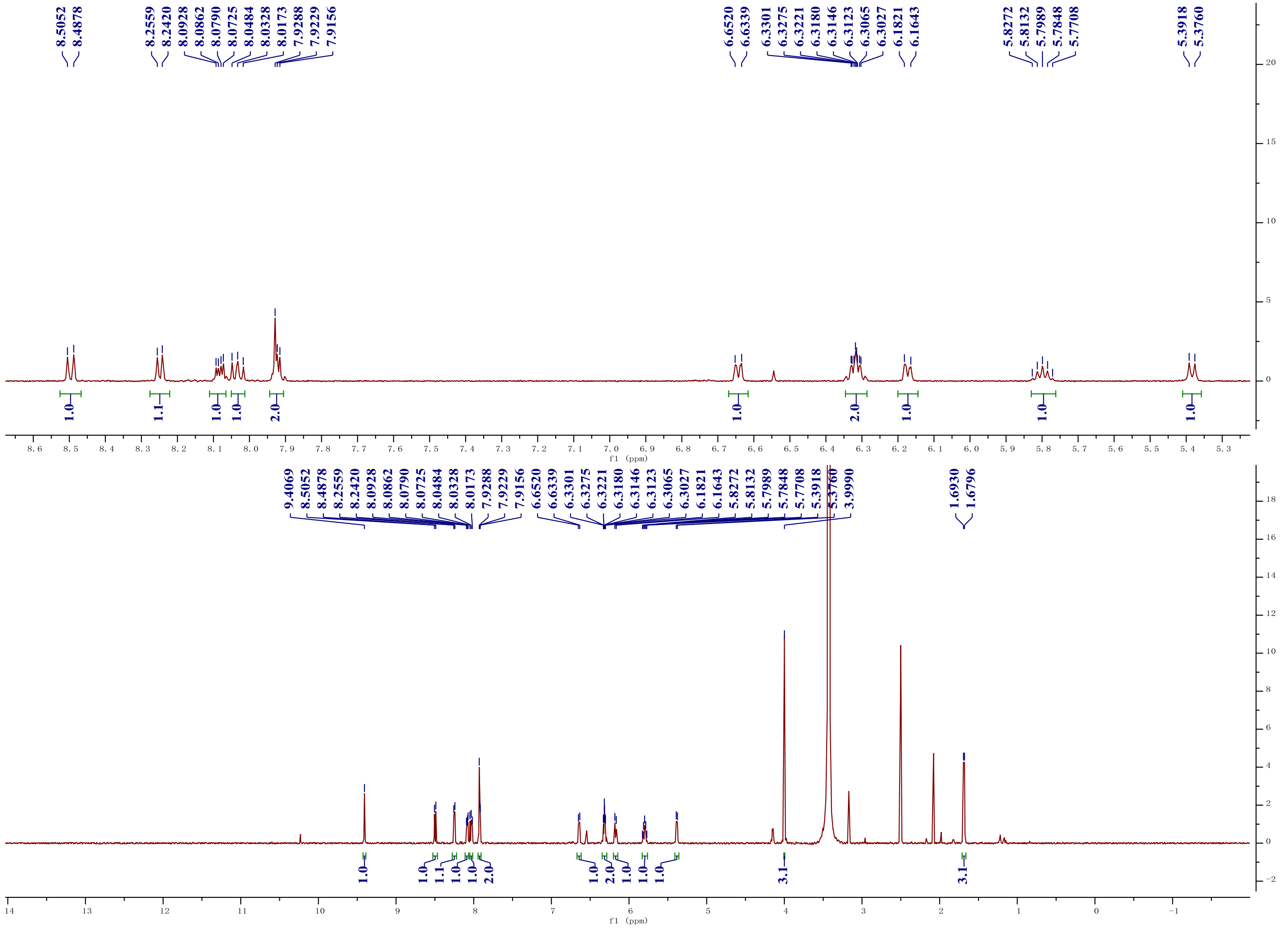

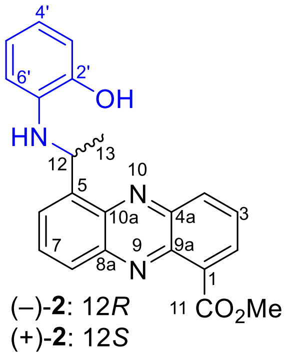

Figure S38. ^{1}H-NMR spectrum (600 MHz) of natural and synthetic **3** in pyridine-d_5

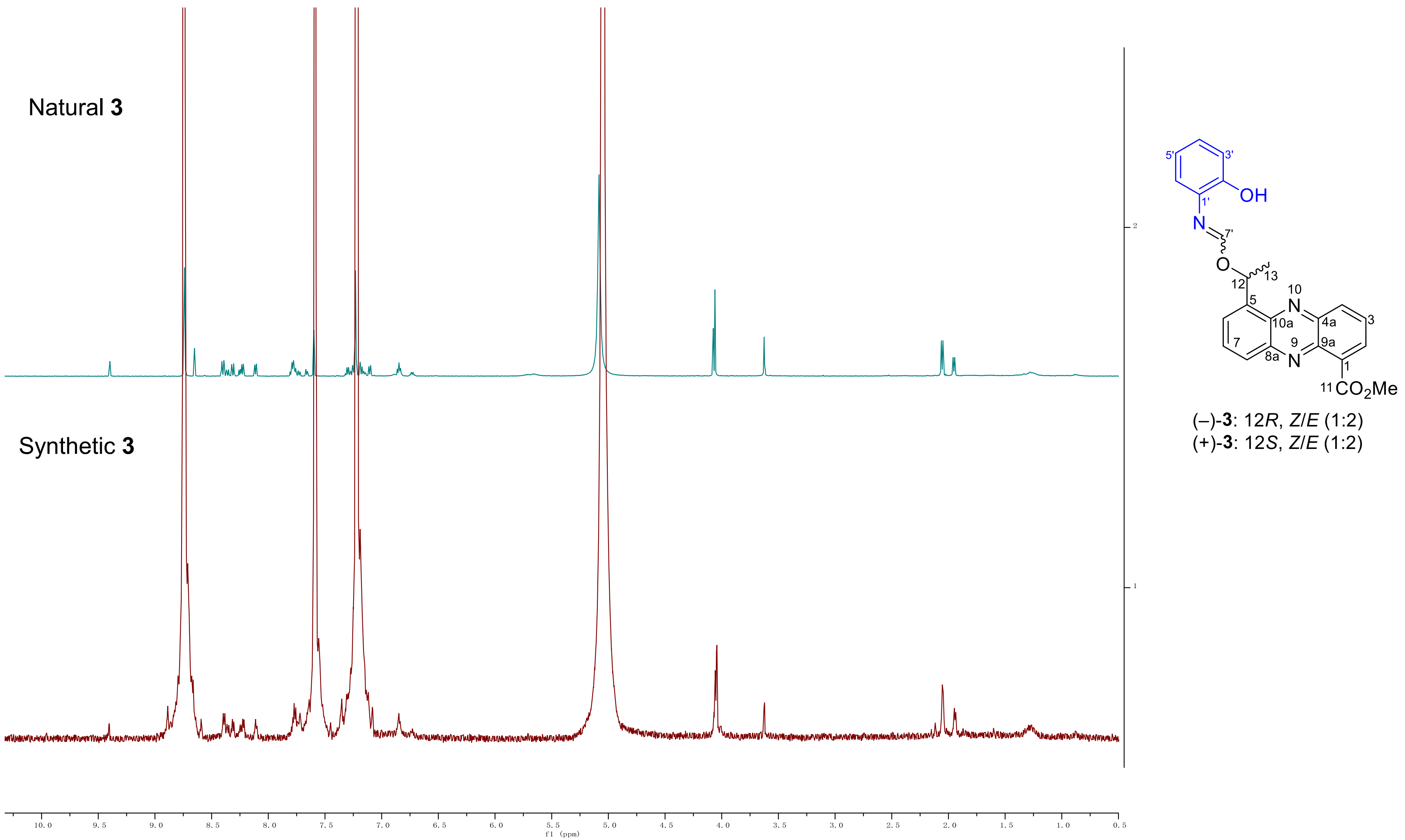

Supplement: Supplementary file 1 — Supplementary material 1. [file 13659_2026_597_MOESM1_ESM.pdf]
